# Evaluating the potential economic and health impact of rotavirus vaccination in 63 middle-income countries not eligible for Gavi funding: a modelling study

**DOI:** 10.1016/S2214-109X(21)00167-4

**Published:** 2021-04-20

**Authors:** Frédéric Debellut, Andrew Clark, Clint Pecenka, Jacqueline Tate, Ranju Baral, Colin Sanderson, Umesh Parashar, Deborah Atherly

**Affiliations:** a Center for Vaccine Innovation and Access, PATH, Geneva, Switzerland; b Department of Health Services Research and Policy, London School of Hygiene & Tropical Medicine, London, UK; c Center for Vaccine Innovation and Access, PATH, Seattle, WA, USA; d Division of Viral Diseases, US Centers for Disease Control and Prevention, Atlanta, GA, USA

## Abstract

**Background:**

Middle-income countries (MICs) that are not eligible for funding from Gavi, the Vaccine Alliance, have been slow to adopt rotavirus vaccines. Few studies have evaluated the cost-effectiveness and benefit–risk of rotavirus vaccination in these settings. We aimed to assess the potential economic and health impact of rotavirus vaccination in 63 MICs not eligible for funding from Gavi.

**Methods:**

In this modelling study, we estimated the cost-effectiveness and benefit–risk of rotavirus vaccination in 63 MICs not eligible to Gavi funding. We used an Excel-based proportionate outcomes model with a finely disaggregated age structure to estimate the number of rotavirus gastroenteritis cases, clinic visits, hospitalisations, and deaths averted by vaccination in children younger than 5 years over a 10-year period. We calculated cost-effectiveness ratios (costs per disability-adjusted life-years averted compared with no vaccination) and benefit–risk ratios (number of hospitalisations due to rotavirus gastroenteritis averted per excess hospitalisations due to intussusception). We evaluated three alternative vaccines available globally (Rotarix, Rotavac, and Rotasiil) and used information from vaccine manufacturers regarding anticipated vaccine prices. We ran deterministic and probabilistic uncertainty analyses.

**Findings:**

Over the period 2020–29, rotavirus vaccines could avert 77 million (95% uncertainty interval [UI] 51–103) cases of rotavirus gastroenteritis and 21 million (12–36) clinic visits, 3 million (1·4–5·6) hospitalisations, and 37 900 (25 900–55 900) deaths due to rotavirus gastroenteritis in 63 MICs not eligible for Gavi support. From a government perspective, rotavirus vaccination would be cost-effective in 48 (77%) of 62 MICs considered. The benefit–risk ratio for hospitalisations prevented versus those potentially caused by vaccination exceeded 250:1 in all countries.

**Interpretation:**

In most MICs not eligible for Gavi funding, rotavirus vaccination has high probability to be cost-effective with a favourable benefit–risk profile. Policy makers should consider this new evidence when making or revisiting decisions on the use of rotavirus vaccines in their respective countries.

**Funding:**

Bill & Melinda Gates Foundation.

## Introduction

Rotavirus infections were responsible for an estimated 151 514 deaths in children younger than 5 years in 2019, along with millions of cases and hospitalisations.^1^ Rotavirus vaccines have been available to low-income and middle-income countries since 2009, and they are cost-effective in most settings,^2^, ^3^, ^4^, ^5^ a finding supported by a 2019 analysis of countries currently and previously supported by Gavi, the Vaccine Alliance.^6^

As of July, 2020, more than 100 countries worldwide had introduced rotavirus vaccines in their national immunisation programmes.^7^ Compared with low-income countries benefiting from international support for vaccine purchase and introduction, adoption of new and underused vaccines has been slower in middle-income countries (MICs) that do not benefit from international support.^8^, ^9^ As of July, 2020, of the 63 MICs that are not currently eligible for financial support from Gavi, only 30 include rotavirus vaccines in their national immunisation programme, with most using Rotarix (manufactured by GlaxoSmithKline, Rixensart, Belgium).^7^, ^10^ Barriers to introducing new vaccines are numerous, with affordability being one of the main challenges reported by countries in the absence of negotiated prices or pooled procurement.

Another possible barrier for MICs has been the perception of the possible risk of intussusception, a rare but serious bowel disorder that can lead to gut perforation or even death in settings without access to timely treatment. A small elevated risk of intussusception has been associated with rotavirus vaccination in some countries, but not in others.^11^, ^12^

Studies exploring the benefits and risks of rotavirus vaccines in MICs estimate that the number of rotavirus deaths prevented by vaccination would greatly exceed any potential deaths caused by intussusception, and WHO maintains its recommendation that all countries introduce rotavirus vaccines in routine immunisation because of their substantial public health benefit.^13^ However, due to continued concerns about rotavirus vaccine-associated intussusception in MICs, and because mortality from both rotavirus and intussusception is rare in those settings,^14^, ^15^ providing benefit–risk ratios for hospitalisations (ie, the number of hospitalisations due to rotavirus prevented for every potential excess hospitalisation due to intussusception) could be useful for decision making.

**Research in context**

**Evidence before this study**

We searched PubMed for studies between Jan 1, 2008, and Feb 28, 2021, using the search terms “rotavirus vaccine”, “middle-income country”, “cost-effectiveness”, and “benefit-risk”, with no language restrictions. We identified four global systematic reviews on the cost-effectiveness of rotavirus vaccination and one global systematic review on benefit–risk analyses. In middle-income countries that are not eligible for support from Gavi, the Vaccine Alliance, we found 28 cost-effectiveness studies; however, none evaluated two new products that were prequalified in 2018. In terms of benefit–risk analyses, we found a modelling study done in 14 countries in Latin America that estimated benefit–risk ratios for deaths and hospital admissions, assuming age-restricted vaccination. Another modelling study covered 135 low-income and middle-income countries and estimated benefit–risk ratios for deaths with and without age restrictions, but did not estimate the benefit–risk of hospital admissions. Therefore, little up-to-date evidence exists examining the cost-effectiveness and benefit–risk of rotavirus vaccination in countries not eligible for Gavi funding.

**Added value of this study**

This study provides estimates of the economic and public health impact of three oral rotavirus vaccines available in the global market for 63 middle-income countries that are not eligible for funding from Gavi. We present comprehensive evidence for decision makers assessing or re-assessing their investment regarding rotavirus vaccination. The model used in this study is available to country teams wishing to scrutinise and improve study inputs, explore additional scenarios, and inform national decision making.

**Implications of all the available evidence**

We found a high probability that rotavirus vaccines represent a good investment for most middle-income countries not eligible for Gavi support, as newer products entering the market expand product choice and enhance market competition. The benefits of rotavirus vaccination are likely to greatly exceed the potential intussusception risk. Countries should consider this new evidence when making or revisiting decisions on the use of these vaccines.

Another important consideration for MICs is the availability of new rotavirus vaccines. For almost a decade, only two rotavirus vaccines, Rotarix and RotaTeq (manufactured by Merck, Kenilworth, NJ, USA), were available. In 2018, WHO prequalified two additional rotavirus vaccines: Rotavac (manufactured by Bharat Biotech, Hyderabad, India) and Rotasiil (manufactured by Serum Institute, Pune, India), both available at lower prices.^16^

Aiming to inform global and country policy makers, this study explores the cost-effectiveness and benefit–risk of three WHO-prequalified and globally available rotavirus vaccines (Rotarix, Rotavac, and Rotasiil) in 63 MICs that are not eligible for funding from Gavi. Although some MICs are already using rotavirus vaccines, many continue to face highly constrained budgets. An updated understanding of the cost-effectiveness of currently available products can help countries assess their previous choices and make comparisons with other vaccines for a potential introduction or switch.

## Methods

### Study design

For this modelling study, we estimated the cost-effectiveness and benefit–risk of rotavirus vaccination from government and societal perspectives in 63 MICs that are not eligible for funding from Gavi, over a 10-year period starting in 2020, using a comparator of no rotavirus vaccination. The study includes both countries already using rotavirus vaccines at the time of this analysis (n=30) and those that have not yet introduced them (panel).

Panel **Countries included in the analysis**

**Lower-middle-income countries**

*Africa region*

Cabo Verde, Eswatini*

*Americas region*

El Salvador*, Guatemala*

*Eastern Mediterranean region*

Egypt, Jordan*, Morocco*, Palestine*, Syria, Tunisia

*Western Pacific region*

Micronesia*, Philippines, Vanuatu

**Upper-middle-income countries**

*Africa region*

Algeria, Botswana*, Equatorial Guinea, Gabon, Mauritius*, Namibia*, South Africa*

*Americas region*

Argentina*, Belize, Brazil*, Colombia*, Costa Rica*, Dominican Republic*, Ecuador*, Grenada, Jamaica, Mexico*, Panama*, Paraguay*, Peru*, Saint Lucia, Saint Vincent and the Grenadines, Suriname, Venezuela*

*Eastern Mediterranean region*

Iran, Iraq*, Lebanon, Libya*

*Europe region*

Albania*, Belarus, Bosnia and Herzegovina, Bulgaria*, Croatia, Kazakhstan, Kosovo, Montenegro, Romania, Russia, Serbia, North Macedonia*, Turkey, Turkmenistan*

*Southeast Asia region*

Maldives, Thailand*

*Western Pacific region*

China, Fiji*, Malaysia, Samoa, Tonga, Tuvalu

We modelled the number of rotavirus gastroenteritis (RVGE) cases, clinic visits, hospitalisations, and deaths that would occur in children younger than 5 years in the absence of rotavirus vaccination in each country. We modelled the same series of outputs for each country assuming they would include rotavirus vaccines in their national immunisation programmes beginning in 2020 to generate the number of health outcomes averted by vaccination, health-care costs averted, and vaccination costs.

We used UNIVAC (version 1.4.09), an Excel-based, deterministic proportionate outcomes model that has been used extensively for country-specific and global analyses.^6^, ^14^, ^17^, ^18^, ^19^, ^20^, ^21^

### Rotavirus disease burden

We estimated the rate of non-severe RVGE cases, non-severe and severe RVGE clinic visits, severe RVGE cases, severe RVGE hospitalisations and RVGE deaths (appendix pp 2–3). We assumed that all non-severe cases were not fatal. Methods for estimating rotavirus disease burden have been described in detail elsewhere.^6^, ^14^ The full list of country-specific data inputs and uncertainty ranges used to model disease burden is available in the appendix (pp 2–5).

We distributed the number of disease events in children younger than 5 years into weeks of age, on the basis of data from more than 90 hospital datasets.^22^ To avoid overstating the potential benefits of rotavirus vaccination, we assumed that the rotavirus mortality rate would decrease in the absence of vaccination by assuming a trend consistent with the decreasing trend in overall under-5 mortality. We used disability-adjusted life-year (DALY) weights for moderate and severe diarrhoea reported by Salomon and colleagues^23^ as proxies for non-severe and severe RVGE.

### Vaccine coverage, coverage timeliness, and efficacy

We used country-specific coverage rates for the first, second, and third doses of diphtheria-tetanus-pertussis vaccine (DTP) as proxies for coverage of the first, second, and third doses of rotavirus vaccine.^24^ Rotarix only requires two doses; therefore, only coverage for the first and second doses of DTP were used for this vaccine. Data for coverage timeliness were taken from Clark and colleagues,^14^ based on methods used in a previous analysis.^25^ Following initial recommendations from WHO based on the need to avoid the peak age of naturally occurring intussusception, some countries implemented an age-restricted schedule for delivery of rotavirus vaccines (first dose before 15 weeks and last dose before 32 weeks). However, more recent work has shown that the positive benefit–risk profile of rotavirus vaccines supports the use of schedules without age restrictions, and WHO has adapted its recommendations.^13^ To further inform MICs on this topic, we undertook our analysis for schedules with and without age restrictions.

Vaccine efficacy input data are drawn from a 2019 pooled analysis of all published randomised controlled trials on oral rotavirus vaccines efficacy, which stratified data by under-5 mortality and dose and extrapolated for each country.^26^ We assumed efficacy against non-severe RVGE was 0·85 times the efficacy against severe RVGE on the basis of a study by Rogawski and colleagues.^27^

### Costs assumptions

We used country-specific estimates published in 2020 of outpatient and inpatient diarrhoea costs as a proxy for RVGE,^28^ accounting for government (health-care costs to the government alone) and societal perspectives (encompassing health-care costs to the government, costs supported by households as they seek care for their sick child, and loss of productivity). Direct medical costs were used as a proxy for health-care costs supported by the government, while direct medical costs plus direct non-medical and indirect costs were used as a proxy of health-care costs from the societal perspective. We modelled the direct medical costs of intussusception because we were unable to locate data on intussusception treatment costs for our list of countries. Detailed calculations and country-specific cost estimates are available in the appendix (pp 6–10). Because of the limited information on health-care costs, we did an uncertainty analysis with a wide range of −50% to +50% of base input.

To understand potential vaccine prices in settings where new vaccine products are not yet widely used, we asked respective manufacturers to provide an illustrative price or price range that might be available to the list of 63 MICs. Merck has reported an inability to continue supplying rotavirus vaccines to countries in west Africa due to supply constraints. Additionally, the company has received new approval to sell vaccines in other markets at prices that far exceed those examined in this analysis. Therefore, RotaTeq was excluded from this study due to uncertainties about product availability at prices comparable with those we used.^29^ Although vaccine prices and ranges have been informed by industry and are consistent with prices reported by UNICEF,^30^ the prices used in this analysis should not be interpreted as the prices that might be offered by any manufacturer to any single country. Rather, they are reflective of the ranges that might be available to MICs given varying prices by country. When making comparisons between products, countries are strongly encouraged to confirm the vaccine prices that will be available for their specific context. All vaccine parameters used in the study, along with details on how price and price ranges were calculated, are presented in the appendix (p 11).

The incremental cost of delivery represents all additional costs required to deliver vaccines other than the direct cost of vaccine procurement and commodities (eg, costs related to personnel, vaccine distribution, and storage). We searched the immunisation delivery cost catalogue for incremental economic delivery costs of vaccines for infants in lower-MICs and upper-MICs.^31^ The search returned eight datapoints from four countries spanning four WHO regions. Because of this scarce evidence, we used the average value across the eight datapoints and explored uncertainty with a wide range of inputs (appendix p 11).

### Cost-effectiveness

Our main outcome was the incremental cost-effectiveness ratio (ICER), expressed in US dollars per DALY averted. We used 0·5 times the per capita gross domestic product (GDP) of each country as a threshold for cost-effectiveness, following the studies by Woods and colleagues^32^ and Ochalek and colleagues.^33^ We used a 3% discount rate on future costs and DALYs when calculating the cost per DALY averted.^34^, ^35^, ^36^ All costs in the analysis are reported in 2018 US dollars. Country-specific undiscounted vaccination programme costs, undiscounted health-care costs averted, and number of fully immunised children were extracted to calculate the net cost of rotavirus vaccination and the net cost per fully immunised child in each country.

### Intussusception and benefit–risk

To account for the potential risk of intussusception after rotavirus vaccination, we calculated benefit–risk ratios for each country, defined as the number of hospital admissions due to rotavirus averted per excess hospital admission due to intussusception. Methods used to calculate the number of excess intussusception cases are described in detail elsewhere.^14^ The relative risk (RR) of intussusception in the periods following the first (days 1–7) and second (days 8–21) doses was taken from a pooled meta-analysis of studies using the self-controlled case series method.^14^ Estimates of the incidence of intussusception in children younger than 5 years and the age distribution of intussusception by week of age under 5 years were taken from a 2019 global review.^37^ In the absence of health-care utilisation data for intussusception, we assumed that the proportion of intussusception cases with access to a hospital would be the same as first DTP dose coverage in the country concerned, and we assumed that all cases without access to care would be fatal.

### Uncertainty analysis

We did a deterministic analysis with our base-case values and a probabilistic analysis with 1000 runs per country. Parameters included in the probabilistic analysis and the statistical distributions used are presented in the appendix (pp 12–13). We built a cost-effectiveness acceptability curve for each vaccine and report the probability that vaccination would be cost-effective at different possible thresholds, including our main reported threshold of 0·5 GDP per capita.

The model assumed equal dose-specific efficacy and duration of protection associated with each dose, irrespective of the product used. Therefore, vaccines with three doses were predicted to have slightly higher impact than products with two doses. As this has not been shown empirically, it should not be over-interpreted. Our estimates of vaccine impact reflect that of the three-dose vaccines (Rotavac and Rotasiil). Equivalent estimates for the two-dose Rotarix vaccine are shown in the appendix (pp 14–15, 18–19, 28–30). We also did the analysis assuming Rotarix would provide an impact equivalent to that of the three-dose vaccines (appendix pp 24–27).

### Role of the funding source

The funder of the study had no role in study design, data collection, data analysis, data interpretation, or writing of the report.

## Results

We present here the vaccine impact and cost-effectiveness results for age-restricted schedules. Equivalent results for age-unrestricted schedules are shown in the appendix (pp 16–19, 22–23). Over the period 2020–29, we estimated that the use of rotavirus vaccines in all MICs not eligible for funding from Gavi has the potential to avert 77 million (95% uncertainty interval [UI] 51–103) cases of RVGE and 21 million (12–36) clinic visits, 3 million (1·4–5·6) hospitalisations, and 37 900 (25 900–55 900) deaths due to RVGE (table 1). These findings would amount to 1·2 million (0·8–1·7) DALYs averted. Discounted costs averted from clinic visits and hospitalisations would be US$826 million (370–1664) from a government perspective and $1182 million (535–2361) from a societal perspective (table 1). MICs not yet using rotavirus vaccines where a substantial disease burden could be averted by their introduction included Egypt, the Philippines, China, and Iran.

**Table 1:** Vaccine impact results over the period 2020–29

		**Averted rotavirus burden, thousands**					Averted health-care costs,*US$ thousands	
		Cases	Visits	Hospitalisations	Deaths	DALYs*	Government perspective	Societal perspective
All 63 MICs		76 797·5 (51 118·2 to 102 829·4)	20 996·0 (12 242·8 to 35 924·7)	3022·8 (1440·0 to 5636·9)	37·9 (25·9 to 55·9)	1162·2 (786·3 to 1710·4)	826 256 (369 634 to 1 664 273)	1 182 397 (535 262 to 2 360 809)
Africa region		2945·8	817·4	126·0	6·8	178·9	45 285	60 807
	Algeria	597·9 (326·5 to 972·2)	211·5 (114·9 to 379·3)	33·8 (15·8 to 63·9)	0·3 (0·1 to 0·7)	8·5 (3·8 to 20·9)	5475 (2408 to 10 753)	7680 (3397 to 15 050)
	Botswana†	95·1 (67·2 to 121·7)	35·5 (22·8 to 53·6)	5·8 (2·9 to 10·0)	0·3 (0·1 to 0·4)	7·4 (4·8 to 10·6)	2805 (1380 to 5251)	3751 (1860 to 6974)
	Cabo Verde	26·9 (19·5 to 33·5)	9·5 (6·4 to 13·8)	1·5 (0·8 to 2·4)	<0·1 (<0·1 to <0·1)	0·5 (0·3 to 0·5)	203 (104 to 346)	288 (150 to 488)
	Equatorial Guinea	36·0 (27·6 to 46·6)	8·0 (5·3 to 12·6)	1·3 (0·6 to 2·5)	0·1 (<0·1 to 0·1)	3·0 (2·2 to 4·1)	1213 (598 to 2452)	1571 (780 to 3153)
	Gabon	87·4 (63·1 to 115·1)	27·2 (17·7 to 42·7)	4·5 (2·3 to 8·2)	0·2 (0·1 to 0·2)	4·6 (3·5 to 5·6)	1950 (989 to 3884)	2622 (1335 to 5194)
	Mauritius†	31·6 (23·5 to 40·8)	10·9 (7·8 to 15·8)	1·7 (1·0 to 2·6)	<0·1 (<0·1 to <0·1)	0·2 (0·1 to 0·2)	823 (443 to 1369)	1132 (617 to 1878)
	Namibia†	125·7 (92·7 to 169·0)	35·3 (22·4 to 60·2)	5·3 (2·7 to 10·4)	0·3 (0·2 to 0·3)	8·0 (6·2 to 9·5)	1295 (631 to 2676)	1813 (893 to 3718)
	South Africa†	1887·5 (1440·8 to 2466·4)	458·6 (279·4 to 825·2)	68·5 (32·2 to 149·5)	5·4 (3·9 to 7·4)	140·2 (102·7 to 193·9)	31 036 (14 439 to 69 501)	41 257 (19 302 to 91 693)
	Eswatini†	57·8 (46·1 to 74·8)	21·0 (15·0 to 31·9)	3·4 (1·8 to 5·8)	0·3 (0·2 to 0·3)	6·5 (5·1 to 8·7)	485 (267 to 880)	692 (384 to 1247)
Americas region		19 980·4	5185·5	727·1	10·0	308·9	216 807	313 957
	Argentina†	1859·5 (1370·0 to 2343·5)	495·3 (334·2 to 772·2)	70·8 (39·1 to 111·2)	0·2 (0·1 to 0·1)	8·7 (6·2 to 11·2)	46 331 (24 083 to 80 950)	62 643 (32 815 to 109 397)
	Belize	20·3 (15·0 to 26·0)	5·4 (3·6 to 8·8)	0·8 (0·4 to 1·2)	<0·1 (<0·1 to <0·1)	0·2 (0·1 to 0·2)	222 (114 to 399)	301 (156 to 541)
	Brazil†	6459·3 (4697·9 to 8250·7)	1840·2 (1214·3 to 2938·7)	267·4 (143·1 to 446·1)	3·8 (2·1 to 6·8)	114·8 (68·2 to 202·2)	27 964 (14 437 to 50 323)	50 450 (26 629 to 90 386)
	Colombia†	1750·9 (1333·7 to 2241·4)	460·8 (310·3 to 756·9)	65·8 (35·0 to 113·9)	0·5 (0·3 to 0·6)	16·6 (12·3 to 21·8)	22 472 (11 804 to 41 858)	31 148 (16 461 to 57 927)
	Costa Rica†	232·9 (182·1 to 297·1)	68·2 (49·0 to 106·9)	10·0 (5·7 to 16·0)	<0·1 (<0·1 to <0·1)	0·9 (0·6 to 1·2)	5346 (2928 to 9214)	7383 (4087 to 12 708)
	Dominican Republic†	522·3 (379·9 to 652·1)	135·9 (88·4 to 215·0)	19·4 (10·2 to 32·0)	0·5 (0·3 to 0·5)	13·0 (9·5 to 16·1)	5437 (2702 to 9755)	7785 (3934 to 13 932)
	Ecuador†	781·7 (573·4 to 989·6)	181·0 (110·0 to 315·3)	25·0 (12·0 to 49·5)	0·4 (0·2 to 0·5)	13·1 (9·1 to 17·9)	8395 (3974 to 17 347)	11 490 (5479 to 23 604)
	El Salvador†	284·7 (223·9 to 362·0)	82·1 (58·0 to 129·8)	12·4 (6·9 to 21·1)	0·2 (0·1 to 0·3)	6·2 (4·5 to 8·7)	610 (337 to 1083)	1096 (613 to 1941)
	Grenada	5·5 (4·1 to 6·8)	1·5 (1·0 to 2·4)	0·2 (0·1 to 0·3)	<0·1 (<0·1 to <0·1)	<0·1 (<0·1 to <0·1)	104 (55 to 183)	144 (76 to 253)
	Guatemala†	601·6 (446·7 to 797·0)	154·5 (98·5 to 268·4)	21·7 (11·3 to 40·8)	0·9 (0·6 to 1·1)	25·4 (19·1 to 32·5)	3934 (1988 to 7816)	5582 (2863 to 11 046)
	Jamaica	119·8 (90·2 to 150·9)	32·0 (21·5 to 50·5)	4·5 (2·4 to 7·2)	<0·1 (<0·1 to <0·1)	0·7 (0·4 to 0·8)	1331 (679 to 2329)	1817 (936 to 3178)
	Mexico†	4756·3 (3469·4 to 6239·6)	1058·9 (555·2 to 2225·5)	134·0 (52·6 to 376·2)	1·4 (0·7 to 2·6)	48·0 (28·6 to 86·6)	76 477 (29 922 to 213 190)	103 936 (41 112 to 285 824)
	Panama†	210·6 (154·1 to 263·4)	60·6 (40·7 to 92·8)	9·0 (4·8 to 14·2)	0·2 (0·1 to 0·2)	5·0 (3·4 to 6·5)	5182 (2631 to 9034)	7296 (3758 to 12 696)
	Paraguay†	229·1 (163·7 to 297·2)	55·9 (32·1 to 101·7)	7·5 (3·4 to 15·9)	0·1 (<0·1 to 0·2)	4·0 (2·3 to 7·2)	1393 (654 to 3124)	2044 (969 to 4543)
	Peru†	965·9 (723·3 to 1264·1)	230·3 (131·5 to 455·3)	30·4 (13·4 to 73·3)	0·4 (0·2 to 0·8)	13·9 (8·3 to 25·1)	10 084 (4585 to 25 638)	14 014 (6440 to 35 251)
	Saint Lucia	5·3 (3·7 to 6·8)	1·2 (0·6 to 2·3)	0·1 (<0·1 to 0·3)	0 (0 to 0)	<0·1 (<0·1 to <0·1)	80 (33 to 202)	111 (46 to 275)
	Saint Vincent and the Grenadines	4·0 (2·8 to 5·0)	0·9 (0·4 to 1·7)	0·1 (0 to 0·2)	0 (0 to <0·1)	<0·1 (<0·1 to <0·1)	47 (19 to 116)	64 (26 to 157)
	Suriname	16·2 (9·8 to 23·3)	4·0 (2·2 to 7·2)	0·6 (0·2 to 1)	<0·1 (<0·1 to <0·1)	0·1 (<0·1 to 0·2)	122 (54 to 244)	175 (79 to 349)
	Venezuela†	1154·5 (864·7 to 1475·5)	316·8 (215·6 to 501·4)	47·4 (26·0 to 79·5)	1·4 (0·9 to 2·1)	38·2 (25·3 to 59·2)	1275 (724 to 2215)	6479 (3656 to 11 289)
Eastern Mediterranean region		17 122·8	4860·7	720·4	11·4	338·3	115 795	162 900
	Egypt	7591·6 (6034·6 to 9659·4)	1998·1 (1285·5 to 3502·4)	281·7 (138·9 to 549·4)	6·5 (5·3 to 8·0)	186·8 (151·3 to 231·8)	29 853 (15 061 to 58 860)	42 330 (21 500 to 83 031)
	Iran	3735·8 (2719·6 to 4736·0)	1250·9 (742·3 to 2120·0)	196·0 (89·5 to 410·8)	1·0 (0·7 to 1·2)	36·4 (26·2 to 45·9)	46 975 (21 057 to 100 830)	65 034 (29 483 to 138 156)
	Iraq†	1995·6 (1468·5 to 2569·7)	526·3 (350·1 to 833·8)	79·5 (43·0 to 134·2)	2·0 (1·3 to 2·9)	57·2 (38·8 to 83·6)	7928 (4115 to 14 344)	12 928 (6830 to 23 280)
	Jordan†	285·9 (144·3 to 414·7)	91·3 (44·3 to 156·2)	14·1 (5·8 to 26·3)	0·1 (0 to 0)	2·2 (1·0 to 3·5)	2011 (812 to 3893)	2891 (1185 to 5568)
	Lebanon	314·6 (229·4 to 396·2)	96·8 (64·7 to 149·7)	14·5 (7·8 to 23·5)	<0·1 (0 to 0)	1·9 (1·4 to 2·3)	8608 (4371 to 15 057)	11 393 (5840 to 19 889)
	Libya†	212·2 (155·4 to 271·5)	58·1 (36·0 to 97·7)	8·3 (4·1 to 15·3)	<0·1 (<0·1 to <0·1)	1·2 (0·7 to 1·6)	3723 (1764 to 7296)	4999 (2393 to 9755)
	Morocco†	1136·5 (840·8 to 1445·0)	365·5 (239·5 to 570·2)	56·2 (29·6 to 96·3)	1·3 (0·9 to 1·8)	38·2 (26·8 to 51·9)	9414 (4739 to 17 024)	12 917 (6539 to 23 279)
	Palestine†	456·1 (361·5 to 566·3)	129·3 (90·5 to 204·0)	19·1 (10·4 to 31·8)	0·1 (<0·1 to 0·1)	3·3 (2·1 to 5·4)	2017 (1096 to 3525)	2931 (1608 to 5112)
	Syria	857·8 (652·1 to 1098·2)	191·7 (133·5 to 303·6)	28·8 (15·9 to 47·7)	0·2 (<0·1 to 1·4)	8·0 (3·1 to 39·8)	886 (506 to 1546)	1523 (872 to 2657)
	Tunisia	536·8 (418·6 to 679·4)	152·7 (104·1 to 246·4)	22·2 (12·0 to 37·9)	0·1 (<0·1 to 0·1)	3·2 (2·1 to 5·0)	4379 (2302 to 7888)	5954 (3148 to 10 706)
Europe region		8639·4	2203·1	299·6	0·8	40·6	146 924	202 147
	Albania†	79·8 (56·9 to 100·7)	19·6 (12·0 to 33·2)	2·6 (1·3 to 4·6)	<0·1 (<0·1 to <0·1)	0·4 (0·2 to 0·5)	652 (315 to 1257)	908 (444 to 1746)
	Belarus	171·4 (86·1 to 248·9)	44·4 (22·1 to 78·5)	6·1 (2·6 to 10·6)	<0·1 (<0·1 to <0·1)	0·5 (0·2 to 0·7)	1896 (776 to 3576)	2608 (1072 to 4921)
	Bosnia and Herzegovina	53·9 (35·9 to 74·8)	14·1 (8·8 to 24·0)	1·9 (1·0 to 3·2)	<0·1 (<0·1 to <0·1)	0·2 (<0·1 to 0·2)	500 (254 to 910)	701 (358 to 1278)
	Bulgaria†	184·8 (135·3 to 235·7)	49·7 (34·0 to 79·1)	6·9 (3·9 to 10·8)	<0·1 (<0·1 to <0·1)	0·8 (0·5 to 1)	2878 (1523 to 4938)	3987 (2125 to 6850)
	Croatia	108·6 (77·9 to 138·9)	22·1 (11·3 to 47·8)	2·5 (0·9 to 7·3)	<0·1 (<0·1 to <0·1)	0·3 (0·1 to 0·4)	2051 (761 to 5807)	2825 (1075 to 7868)
	Kazakhstan	793·7 (550·1 to 1059·5)	185·2 (115·9 to 329·5)	23·8 (12·4 to 42·1)	0·2 (<0·1 to 0·3)	6·0 (3·4 to 11·2)	7584 (3748 to 14 417)	11 069 (5542 to 21 050)
	Kosovo	27·6 (19·5 to 35·5)	5·4 (2·7 to 11·4)	0·6 (0·2 to 1·7)	0 (0 to 0)	0·1 (<0·1 to 0·1)	74 (30 to 199)	114 (47 to 302)
	Montenegro	20·3 (14·4 to 26·9)	4·2 (2·1 to 9·1)	0·5 (0·1 to 1·4)	0 (0 to 0)	<0·1 (<0·1 to <0·1)	212 (82 to 618)	295 (116 to 848)
	Romania	551·5 (402·9 to 703·9)	157·3 (101·8 to 258·1)	22·7 (11·8 to 39·9)	<0·1 (<0·1 to <0·1)	2·4 (1·5 to 3·7)	11 060 (5460 to 21 010)	15 475 (7747 to 29 265)
	Russia	2779·1 (1404·4 to 4032·2)	777·3 (384·4 to 1376·5)	111·0 (48·1 to 202·5)	0·1 (<0·1 to 0·1)	9·7 (4·8 to 14·7)	72 289 (29 254 to 140 056)	97 345 (39 933 to 188 281)
	Serbia	238·1 (173·7 to 311·4)	48·6 (25·3 to 106·5)	5·5 (2·0 to 16·3)	<0·1 (<0·1 to <0·1	0·6 (0·3 to 0·9)	1891 (728 to 5382)	2660 (1049 to 7447)
	North Macedonia†	32·3 (18·9 to 49·7)	8·5 (4·8 to 15·4)	1·2 (0·5 to 2·0)	<0·1 (<0·1 to <0·1)	0·2 (<0·1 to 0·2)	360 (166 to 694)	494 (229 to 954)
	Turkey	3386·1 (2453·3 to 4252·4)	821·0 (506·2 to 1399·8)	108·9 (54·5 to 195·8)	0·2 (0·1 to 0·2)	13·5 (9·3 to 18·0)	43 539 (20 750 to 84 587)	60 967 (29 433 to 117 978)
	Turkmenistan	212·2 (149·7 to 272·2)	45·7 (26·3 to 85·1)	5·5 (2·5 to 11·1)	0·2 (<0·1 to 0·7)	6·2 (2·4 to 20·3)	1939 (913 to 4249)	2699 (1282 to 5881)
Southeast Asia region		2233·0	730·2	112·7	0·4	16·6	26 481	37 983
	Maldives	20·6 (14·8 to 25·8)	5·8 (3·7 to 9·1)	0·8 (0·4 to 1·3)	<0·1 (<0·1 to <0·1)	0·1 (<0·1 to 0·1)	301 (151 to 540)	429 (218 to 768)
	Thailand†	2212·4 (1588·5 to 2810·1)	724·5 (447·6 to 1177·8)	111·8 (54·2 to 211·8)	0·4 (0·3 to 0·5)	16·6 (11·7 to 21·3)	26 180 (12 250 to 51 711)	37 553 (17 765 to 73 524)
Western Pacific region		25 876·1	7199·1	1037·1	8·4	278·8	274 964	404 603
	China	21 482·2 (10 898·5 to 31 197·0)	6055·3 (2986·2 to 10 339·6)	873·0 (378·1 to 1524·7)	3·6 (1·8 to 5·2)	146·5 (73·9 to 212·9)	238 115 (96 566 to 444 850)	351 769 (143 719 to 656 210)
	Fiji†	48·0 (36·3 to 59·6)	13·2 (8·7 to 20·9)	1·9 (1·0 to 3·0)	<0·1 (<0·1 to <0·1)	1·1 (0·8 to 1·3)	346 (178 to 606)	511 (265 to 893)
	Malaysia	1761·7 (1276·1 to 2222·7)	477·7 (278·7 to 842·6)	67·0 (30·5 to 137·6)	0·1 (<0·1 to 0·1)	7·5 (5·1 to 10·3)	26 225 (11 669 to 55 756)	37 460 (16 945 to 78 843)
	Micronesia†	4·4 (3·1 to 5·6)	1·1 (0·7 to 1·8)	0·2 (0 to 0·2)	<0·1 (<0·1 to <0·1)	0·1 (<0·1 to 0·3)	32 (16 to 58)	43 (22 to 80)
	Philippines	2551·3 (1857·9 to 3358·5)	645·5 (403·3 to 1094·0)	94·1 (47·1 to 176·1)	4·5 (3·2 to 6·0)	122·9 (88·4 to 165·8)	10 038 (5132 to 20 275)	14 538 (7478 to 29 180)
	Samoa	7·1 (5·4 to 8·8)	1·7 (1·1 to 2·8)	0·2 (0·1 to 0·4)	<0·1 (<0·1 to <0·1)	<0·1 (<0·1 to <0·1)	45 (23 to 80)	63 (33 to 113)
	Tonga	5·9 (4·5 to 7·4)	1·4 (0·8 to 2·2)	0·2 (0·1 to 0·3)	<0·1 (<0·1 to <0·1)	<0·1 (<0·1 to <0·1)	39 (20 to 71)	55 (29 to 99)
	Tuvalu	0·5 (0·3 to 0·5)	0·1 (0 to 0·2)	0·0 (0 to 0)	0 (0 to <0·1)	<0·1 (<0·1 to <0·1)	6 (3 to 11)	8 (4 to 14)
	Vanuatu	14·9 (10·8 to 19·4)	3·1 (1·9 to 5·0)	0·4 (0·2 to 0·7)	<0·1 (<0·1 to <0·1	0·6 (0·3 to 1·1)	118 (61 to 220)	156 (81 to 291)

Data are estimates (95% uncertainty interval) compared with no vaccination and assuming a three-dose, age-restricted schedule in all countries. DALYs=disability-adjusted life-years. MIC=middle-income country.

* Discounted values at 3% per year.

† Countries using rotavirus vaccine as part of their national immunisation programme as of July 9, 2020.^7^, ^10^

In all countries, vaccination costs were estimated to be systematically lower with Rotavac and Rotasiil than with Rotarix, with a slight cost advantage to Rotasiil (table 2). The estimated net cost per fully immunised child, accounting for the difference in undiscounted vaccination programme costs and undiscounted averted treatment costs, ranged from −$5 with Rotasiil in Lebanon to $33 with Rotarix in Venezuela (appendix pp 33–42).

**Table 2:** Cost-effectiveness results per country in the period 2020–29

	Vaccination costs, US$ millions*			Cost per DALY averted–government perspective,*US$			**Country GDP per capita, US$**	Probability for cost per DALY averted to be <0·5 GDP per capita, government perspective*		
	Rotarix	Rotavac	Rotasiil	Rotarix	Rotavac	Rotasiil		Rotarix	Rotavac	Rotasiil
**Africa region**										
Algeria	69·99 (39·92–105·48)	28·98 (16·11–43·10)	24·74 (13·71–36·88)	8332 (3520–15 992)	2781 (1024–5418)	2278 (789–4530)	4115	0%	41%	58%
Botswana†	11·01 (8·15–13·01)	4·37 (3·21–5·04)	3·73 (2·73–4·27)	1240 (572–2052)	211 (CS–549)	125 (CS–436)	8259	100%	100%	100%
Cabo Verde	2·03 (1·50–2·41)	0·76 (0·54–0·88)	0·65 (0·46–0·75)	3992 (2765–5488)	1186 (624–1723)	949 (434–1414)	3635	0%	99%	100%
Equatorial Guinea	4·00 (3·15–4·84)	1·44 (1·11–1·65)	1·23 (0·95–1·41)	1017 (427–1660)	74 (CS–347)	4 (CS–258)	10 262	100%	100%	100%
Gabon	10·36 (7·91–12·61)	4·15 (3·15–4·82)	3·54 (2·67–4·10)	2007 (1253–2880)	476 (15–823)	345 (CS–662)	7953	100%	100%	100%
Mauritius†	2·59 (1·91–3·15)	1·06 (0·79–1·25)	0·91 (0·66–1·06)	7615 (4222–10 909)	1009 (CS–2836)	358 (CS–2095)	11 239	15%	100%	100%
Namibia†	15·32 (12·34–18·39)	6·26 (4·98–7·17)	5·34 (4·23–6·09)	1913 (1377–2571)	622 (349–861)	507 (256–709)	5931	100%	100%	100%
South Africa†	194·22 (148·26–237·72)	77·76 (58·90–90·81)	66·37 (49·85–77·44)	1281 (703–1892)	333 (22–566)	252 (CS–464)	6374	100%	100%	100%
Eswatini†	6·50 (5·23–7·78)	2·65 (2·11–3·02)	2·26 (1·78–2·58)	1007 (678–1386)	333 (191–468)	273 (146–391)	4146	100%	100%	100%
**Americas region**										
Argentina†	141·74 (104·80–172·55)	52·51 (37·65–61·91)	44·81 (32·07–52·94)	11 413 (5871–17 481)	712 (CS–3672)	CS (CS–2599)	11 684	3%	100%	100%
Belize	1·55 (1·16–1·88)	0·58 (0·42–0·70)	0·50 (0·35–0·60)	6837 (4814–8991)	1804 (653–2659)	1378 (304–2148)	4885	0%	94%	100%
Brazil†	491·55 (360·34–603·76)	182·53 (129·76–217·45)	155·78 (110·76–185·19)	4147 (2173–6884)	1347 (633–2253)	1114 (493–1881)	8921	68%	100%	100%
Colombia†	133·86 (102·05–164·96)	50·00 (36·85–59·71)	42·67 (31·32–50·83)	6903 (4322–9862)	1656 (267–2835)	1215 (CS–2288)	6668	0%	100%	100%
Costa Rica†	14·38 (11·29–17·40)	5·62 (4·35–6·52)	4·79 (3·68–5·56)	10 151 (3982–17 291)	299 (CS–3634)	CS (CS–2532)	12 027	12%	100%	100%
Dominican Republic†	40·04 (29·58–47·40)	14·85 (10·55–17·21)	12·67 (8·94–14·68)	2733 (1812–3793)	722 (257–1123)	555 (127–913)	8051	99%	100%	100%
Ecuador†	59·78 (44·40–72·67)	22·40 (16·10–26·43)	19·12 (13·68–22·56)	4047 (2320–6029)	1071 (190–1810)	820 (31–1497)	6345	23%	100%	100%
El Salvador†	21·66 (17·27–26·50)	8·48 (6·66–9·83)	7·24 (5·65–8·41)	3482 (2310–4990)	1262 (784–1751)	1063 (642–1492)	4058	0%	100%	100%
Grenada	0·34 (0·26–0·41)	0·13 (0·10–0·15)	0·11 (0·08–0·13)	6738 (2596–11 968)	800 (CS–3037)	251 (CS–2199)	10 640	37%	100%	100%
Guatemala†	77·04 (57·13–94·05)	27·18 (19·41–33·04)	23·19 (16·41–28·19)	3087 (2214–4017)	917 (560–1210)	760 (438–1007)	4549	4%	100%	100%
Jamaica	9·04 (6·82–10·83)	3·38 (2·45–3·93)	2·88 (2·08–3·36)	11 978 (7808–17 211)	3063 (1012–4966)	2322 (392–3996)	5354	0%	46%	71%
Mexico†	366·85 (270·40–459·18)	135·57 (96·25–165·67)	115·70 (81·71–140·71)	6212 (1786–11 464)	1230 (CS–3333)	816 (0–2674)	9673	42%	100%	100%
Panama†	16·06 (11·88–19·12)	5·90 (4·22–6·89)	5·04 (3·57–5·87)	2267 (1012–3708)	145 (CS–783)	CS (CS–567)	15 575	100%	100%	100%
Paraguay†	26·84 (19·95–32·61)	10·00 (7·19–11·79)	8·54 (6·11–10·06)	6887 (3427–11 877)	2129 (890–3729)	1767 (694–3137)	5822	1%	88%	96%
Peru†	115·40 (90·99–140·37)	41·41 (31·53–48·53)	35·34 (26·84–41·52)	8221 (3786–14 425)	2256 (615–4260)	1819 (372–3552)	6941	2%	91%	97%
Saint Lucia	0·41 (0·30–0·49)	0·15 (0·11–0·18)	0·13 (0·09–0·15)	24 522 (7893–42 403)	5206 (CS–12 000)	3595 (CS–9598)	10 566	1%	68%	80%
Saint Vincent and the Grenadines	0·31 (0·22–0·36)	0·11 (0·08–0·13)	0·10 (0·07–0·11)	25 454 (7226–43 123)	6452 (CS–12 896)	4854 (CS–10 604)	7361	0%	44%	58%
Suriname	1·23 (0·77–1·66)	0·50 (0·30–0·66)	0·42 (0·26–0·57)	8457 (4499–13 663)	2758 (1132–4817)	2222 (782–4022)	6234	0%	73%	88%
Venezuela†	101·89 (78·14–121·99)	48·88 (37·69–56·37)	44·41 (34·27–51·14)	2684 (1679–3973)	1245 (780–1774)	1128 (705–1620)	16 708	100%	100%	100%
**Eastern Mediterranean region**										
Egypt	560·15 (446·91–664·26)	225·75 (178·36–255·36)	192·67 (151·04–217·31)	2936 (2081–3872)	1048 (657–1370)	871 (511–1160)	2549	0%	92%	100%
Iran	282·12 (208·33–334·02)	106·06 (75·04–122·86)	90·51 (63·7–104·60)	6669 (3839–9417)	1625 (0–2767)	1198 (0–2257)	5550	0%	98%	100%
Iraq†	225·26 (167·66–270·74)	82·72 (59·51–97·06)	70·60 (50·37–82·49)	4087 (2597–5907)	1308 (759–1839)	1096 (621–1552)	5834	9%	100%	100%
Jordan†	22·88 (11·73–31·20)	9·48 (4·75–12·75)	8·09 (4·07–10·90)	9497 (5799–14 016)	3318 (1671–5003)	2701 (1223–4195)	4242	0%	14%	34%
Lebanon	20·54 (15·13–24·60)	7·44 (5·31–8·68)	6·35 (4·50–7·40)	6374 (2052–10 352)	CS (CS–1690)	CS (CS–1029)	8270	18%	100%	100%
Libya†	23·80 (17·55–28·29)	8·92 (6·32–10·35)	7·62 (5·37–8·82)	19 151 (10 969–28 858)	4497 (611–8079)	3366 (CS–6454)	7242	0%	46%	66%
Morocco†	132·18 (97·66–156·45)	52·95 (38·61–60·82)	45·19 (32·79–51·78)	3502 (2271–4970)	1140 (643–1637)	937 (491–1361)	3273	0%	97%	100%
Palestine†	34·32 (27·58–40·33)	13·88 (11·04–15·58)	11·85 (9·27–13·32)	10 171 (5609–16 294)	3604 (1803–5835)	2986 (1430–4927)	3199	0%	2%	7%
Syria	59·98 (45·96–73·08)	21·63 (16·25–25·22)	18·46 (13·76–21·48)	7601 (1463–18 920)	2605 (485–6523)	2207 (404–5455)	No data	NA	NA	NA
Tunisia	39·15 (30·58–46·46)	15·00 (11·40–17·25)	12·80 (9·71–14·63)	11 335 (6271–17 834)	3324 (1298–5587)	2636 (841–4626)	3448	0%	10%	27%
**Europe region**										
Albania†	6·13 (4·45–7·31)	2·31 (1·62–2·70)	1·97 (1·37–2·30)	15 424 (8155–24 957)	4520 (1651–7776)	3597 (1055–6440)	5269	0%	18%	37%
Belarus	10·64 (5·48–14·56)	4·41 (2·20–5·96)	3·76 (1·88–5·07)	19 188 (10 755–30 263)	5471 (1260–9848)	4063 (229–7852)	6290	0%	20%	43%
Bosnia and Herzegovina	3·42 (2·28–4·58)	1·28 (0·84–1·67)	1·09 (0·71–1·42)	19 010 (10 995–29 237)	5025 (1616–8740)	3816 (654–7055)	6066	0%	20%	42%
Bulgaria†	11·72 (8·60–14·21)	4·66 (3·40–5·48)	3·98 (2·89–4·69)	11 781 (6589–17 662)	2355 (CS–4931)	1453 (CS–3795)	9273	0%	95%	100%
Croatia	6·90 (4·99–8·29)	2·56 (1·81–3·02)	2·18 (1·53–2·58)	18 343 (1772–33 455)	1901 (CS–8680)	498 (CS–6675)	14 910	14%	95%	99%
Kazakhstan	57·84 (40·70–71·31)	21·31 (14·48–26·24)	18·19 (12·25–22·39)	9050 (4115–15 623)	2297 (577–4445)	1775 (277–3640)	9813	9%	99%	100%
Kosovo	3·28 (2·41–3·81)	1·23 (0·88–1·42)	1·05 (0·75–1·21)	52 482 (31 281–84 736)	17 319 (9418–27 410)	14 619 (7633–23 297)	4302	0%	0%	0%
Montenegro	1·30 (0·93–1·61)	0·52 (0·37–0·62)	0·44 (0·32–0·53)	22 385 (9263–36 621)	6276 (CS–12 338)	4722 (CS–10 031)	8844	0%	48%	64%
Romania	35·14 (25·82–42·56)	9·70 (7·05–11·26)	8·28 (6·00–9·61)	9977 (3556–17 579)	CS (CS–2207)	CS (CS–1385)	12 301	18%	100%	100%
Russia	172·16 (89·40–234·06)	71·29 (35·54–96·41)	60·84 (30·51–81·75)	10 418 (2850–17 914)	CS (CS–3829)	CS (CS–2627)	11 473	14%	100%	100%
Serbia	15·16 (11·16–18·50)	6·13 (4·44–7·26)	5·23 (3·77–6·20)	23 145 (10 789–37 110)	7316 (435–13 037)	5765 (CS–10 696)	7247	0%	23%	39%
North Macedonia†	2·01 (1·18–2·90)	0·83 (0·48–1·18)	0·71 (0·41–1·00)	10 590 (6169–15 940)	2998 (737–5282)	2223 (172–4190)	6084	0%	61%	83%
Turkey	258·15 (189·89–306·24)	96·79 (68·48–112·01)	82·60 (58·15–95·64)	16 402 (10 045–23 684)	3946 (352–6831)	2895 (CS–5389)	9370	0%	74%	92%
Turkmenistan†	24·89 (18·42–29·38)	9·97 (7·32–11·42)	8·51 (6·20–9·73)	4068 (1063–10 388)	1297 (270–3417)	1061 (196–2788)	6967	63%	98%	99%
**Southeast Asia region**										
Maldives	1·28 (0·94–1·52)	0·48 (0·34–0·56)	0·41 (0·29–0·48)	11 972 (6782–17 844)	2185 (CS–4667)	1333 (CS–3578)	10 331	1%	99%	100%
Thailand†	136·64 (98·95–163·34)	51·09 (35·86–60·08)	43·60 (30·43–51·21)	6738 (3847–9748)	1505 (CS–2771)	1052 (CS–2252)	7274	2%	100%	100%
**Western Pacific region**										
China	1664·03 (856·58–2261·45)	689·09 (343·55–931·53)	588·10 (294·61–785·43)	9944 (6450–13 834)	3078 (1282–4699)	2389 (691–3811)	9771	0%	98%	100%
Fiji†	3·64 (2·77–4·35)	1·50 (1·13–1·71)	1·28 (0·96–1·45)	3077 (2167–4164)	1048 (595–1467)	848 (437–1227)	6267	57%	100%	100%
Malaysia	108·85 (80·07–129·11)	44·39 (32·29–50·84)	37·88 (27·44–43·45)	11 203 (5005–17 617)	2432 (CS–5487)	1561 (CS–4344)	11 373	5%	98%	100%
Micronesia†	0·52 (0·39–0·62)	0·19 (0·13–0·21)	0·16 (0·11–0·18)	5396 (1259–14 515)	1562 (322–4353)	1286 (249–3557)	3568	13%	78%	83%
Philippines	305·02 (232·92–372·77)	125·08 (94·72–146·12)	106·75 (80·22–124·51)	2640 (1787–3723)	936 (594–1305)	787 (479–1106)	3103	1%	100%	100%
Samoa	0·52 (0·41–0·63)	0·19 (0·15–0·22)	0·16 (0·13–0·19)	9877 (5079–16 349)	2934 (1212–5218)	2372 (869–4357)	4183	0%	32%	53%
Tonga	0·45 (0·35–0·55)	0·18 (0·14–0·21)	0·16 (0·12–0·18)	10 007 (5264–16 341)	3380 (1496–5858)	2748 (1099–4915)	4364	0%	22%	41%
Tuvalu	0·06 (0·04–0·07)	0·02 (0·02–0·03)	0·02 (0·01–0·02)	22 826 (1545–55 082)	6820 (372–17 918)	5470 (265–14 563)	3701	5%	47%	55%
Vanuatu	2·10 (1·63–2·49)	1·15 (0·94–1·30)	1·05 (0·85–1·18)	3475 (1723–6001)	1655 (794–2837)	1487 (704–2572)	3124	1%	56%	66%

Data are estimates (95% uncertainty interval), unless otherwise specified. Estimates assume a three-dose age-restricted schedule in all countries. Each vaccine is compared with no vaccination. All data are in 2018 US dollars. CS=cost saving. DALYs=disability-adjusted life-years. GDP=gross domestic product. NA=not applicable.

* Countries using rotavirus vaccine as part of their national immunisation programme, as of July 9, 2020.^7^, ^10^

† Values discounted at 3% per year.

We estimated that, from the government perspective, 48 (77%) of 62 MICs (excluding Syria, as no GDP data was available at the time of this study) have a deterministic ICER lower than 0·5 times their GDP per capita with at least one of the rotavirus products under consideration (table 2). This includes 21 of the 33 countries not yet using rotavirus vaccines. When evaluating from the societal perspective, the number of countries with an ICER lower than the threshold rose to 54 (87%) of 62 countries. Deterministic ICERs from the government and societal perspectives and a comparison with the threshold are available in the appendix (pp 20–23).

Both Rotavac and Rotasiil were estimated to have a 90% or higher probability of being cost-effective at the 0·5 GDP per capita threshold in 39 (Rotavac) and 41 (Rotasiil) countries (table 2). Rotarix has a 90% or higher chance of being cost-effective at the same threshold in nine countries. We estimated that, for 17 countries not yet using rotavirus vaccines nationally, at least one product had a 90% or higher chance of being cost-effective at the 0·5 GDP per capita threshold. If these 17 countries were to use rotavirus vaccines, they would prevent 16 759 (95% UI 11 920–22 086) RVGE deaths over 10 years (appendix p 43).

Overall, we estimated Rotasiil to be the least costly and most cost-effective choice for all countries, followed closely by Rotavac. The cost-effectiveness acceptability curve for this dominant option (figure) highlights countries where rotavirus vaccination is less likely to be cost-effective at a 0·5 GDP per capita threshold. Countries in which the probability of rotavirus vaccines being cost-effective was lower than 50% (using 0·5 GDP per capita as the threshold) include two in the eastern Mediterranean region (Palestine and Jordan), five in the European region (Albania, Belarus, Bosnia and Herzegovina, Kosovo, and Serbia), and one in the western Pacific region (Tonga). Acceptability curves for Rotarix and Rotavac are available in the appendix (pp 31–32). Although Rotavac showed similar probabilities of being cost-effective to Rotasiil, Rotarix probabilities were lower than both products.

**Figure:**
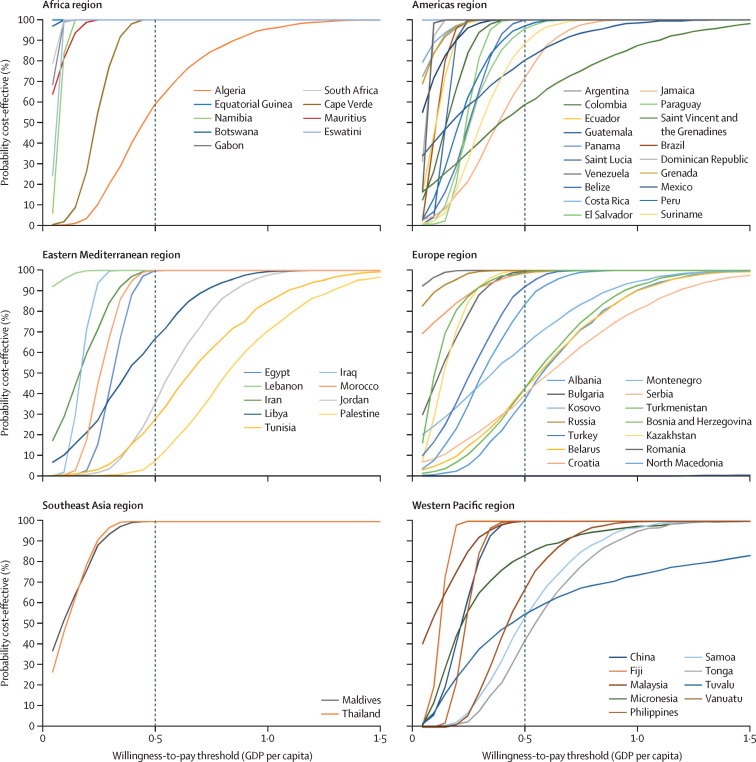
Cost-effectiveness acceptability curves for the dominant vaccine (Rotasiil) compared with no vaccination GDP=gross domestic product.

With strict adherence to age restrictions, we estimated that rotavirus vaccines could prevent about 3 million RVGE hospital admissions across all 63 countries over a 10-year period, increasing to 4·5 million without age restrictions (table 3). Over the same period, hospital admissions caused by excess cases of intussusception would be approximately 2900 with age restrictions or 6700 without age restrictions. The benefit–risk ratio was approximately 1000:1 with age restrictions and 670:1 without age restrictions. The incremental benefit–risk among children who would receive the vaccine outside the recommended age windows was approximately 383:1 and was not lower than 262:1 in any of the countries assessed (table 3).

**Table 3:** Risk–benefit of age-restricted and unrestricted schedules of vaccination for the period 2020–29

		**Averted RVGE hospitalisations**			**Excess intussusception hospitalisations**			**RVGE hospitalisations averted per excess intussusception hospitalisation**		
		Age-restricted schedule	Age-unrestricted schedule	Unrestricted *vs* restricted	Age-restricted schedule	Age- unrestricted schedule	Unrestricted *vs* restricted	Age-restricted schedule	Age-unrestricted schedule	Unrestricted *vs* restricted
All 63 MICs		3 022 783	4 475 501	1 452 717	2892	6687	3795	1045	669	383
Africa region		125 956	199 652	73 696	93	193	100	1357	1035	737
	Algeria	33 837	98 579	64 742	25	100	76	1378	985	857
	Botswana*	5818	6760	942	3	4	1	2098	1862	1100
	Cabo Verde	1524	1752	228	1	1	0	2770	2471	1436
	Equatorial Guinea	1327	1466	140	1	1	0	1733	1417	517
	Gabon	4467	5156	689	2	3	1	2356	1877	810
	Mauritius*	1724	1979	256	0	1	0	3568	2903	1286
	Namibia*	5320	5355	35	2	2	0	2712	2643	548
	South Africa*	68 535	75 199	6664	59	81	22	1159	926	302
	Eswatini*	3404	3405	1	1	1	0	4828	4828	2834
Americas region		727 106	841 785	114 679	390	541	150	1862	1557	764
	Argentina*	70 802	80 813	10 011	26	32	7	2769	2514	1522
	Belize	769	936	168	0	0	0	2597	2285	1475
	Brazil*	267 410	305 654	38 244	21	26	5	12 562	11 582	7495
	Colombia*	65 831	75 356	9525	33	42	9	1989	1806	1102
	Costa Rica*	9979	10 300	321	2	2	0	5756	5627	3320
	Dominican Republic*	19 417	22 178	2761	10	13	3	1905	1728	1047
	Ecuador*	24 965	28 687	3722	14	18	4	1778	1608	979
	El Salvador*	12 365	12 529	164	5	5	0	2502	2502	2441
	Grenada	214	243	29	0	0	0	3185	2747	1368
	Guatemala*	21 713	27 781	6068	19	27	8	1132	1029	777
	Jamaica	4537	5179	642	2	2	1	2651	2273	1131
	Mexico*	134 045	165 614	31 569	203	303	100	661	546	314
	Panama*	8954	10 173	1219	3	3	1	3579	3257	1961
	Paraguay*	7541	8879	1337	6	8	2	1195	1113	803
	Peru*	30 355	33 427	3073	20	22	2	1510	1509	1501
	Saint Lucia	141	161	20	0	0	0	1464	1327	805
	Saint Vincent and the Grenadines	104	119	15	0	0	0	1442	1305	794
	Suriname	553	962	409	0	1	0	2141	1560	1141
	Venezuela*	47 412	52 794	5381	26	36	10	1819	1483	564
Eastern Mediterranean region		720 361	796 815	76 454	704	865	161	1023	922	476
	Egypt	281 721	286 688	4967	196	202	6	1439	1420	810
	Iran	195 956	225 669	29 713	171	218	47	1147	1034	628
	Iraq*	79 537	90 621	11 084	138	173	35	575	523	318
	Jordan*	14 057	28 286	14 229	17	48	31	806	583	458
	Lebanon	14 520	16 300	1780	12	15	3	1163	1054	595
	Libya*	8279	9679	1400	15	18	4	571	523	352
	Morocco*	56 221	64 536	8316	75	98	23	749	660	366
	Palestine*	19 106	19 248	143	22	23	0	857	855	606
	Syria	28 766	31 970	3204	37	46	9	775	694	359
	Tunisia	22 198	23 817	1619	20	22	2	1101	1061	708
Europe region		299 645	464 244	164 600	193	327	134	1553	1419	1226
	Albania*	2599	2991	392	2	2	0	1398	1307	912
	Belarus	6066	12 876	6811	2	6	3	2732	2292	2005
	Bosnia and Herzegovina	1938	2847	909	1	1	0	3017	2542	1903
	Bulgaria*	6932	7921	989	3	4	1	2032	1878	1229
	Croatia	2509	2857	348	2	3	0	1197	1118	758
	Kazakhstan	23 793	32 480	8686	17	27	11	1418	1188	824
	Kosovo	624	738	114	1	1	0	629	604	497
	Montenegro	472	580	108	0	1	0	1117	1022	745
	Romania	22 702	26 273	3571	11	13	2	2132	2038	1591
	Russia	110 965	233 854	122 889	63	156	93	1775	1504	1321
	Serbia	5500	6604	1105	5	6	1	1127	1043	761
	North Macedonia*	1178	2674	1497	1	2	1	1648	1356	1189
	Turkey	108 855	125 114	16 259	79	96	18	1387	1297	904
	Turkmenistan*	5513	6434	921	7	9	2	756	711	523
Southeast Asia region		112 655	128 943	16 288	46	57	11	2470	2279	1486
	Maldives	822	944	122	1	1	0	1373	1211	674
	Thailand*	111 833	127 999	16 167	45	56	11	2485	2294	1499
Western Pacific region		1 037 062	2 044 061	1 007 000	1466	4705	3239	707	434	311
	China	873 014	1 856 187	983 173	1337	4530	3194	653	410	308
	Fiji*	1877	2127	250	1	2	0	1371	1146	513
	Malaysia	67 013	76 718	9704	11	15	4	5990	5137	2589
	Micronesia*	160	182	23	0	0	0	615	582	424
	Philippines	94 124	107 866	13 743	115	156	40	816	693	341
	Samoa	240	264	24	0	0	0	1174	1013	432
	Tonga	189	212	23	0	0	0	1100	929	410
	Tuvalu	18	20	2	0	0	0	807	697	343
	Vanuatu	428	485	57	1	1	0	624	537	262

Data are estimates. Results shown for three-dose vaccines. MIC=middle-income country. RVGE=rotavirus gastroenteritis.

* Countries using rotavirus vaccine as part of their national immunisation programme, as of July 9, 2020.^7^, ^10^

## Discussion

Rotavirus vaccination has the potential to avert substantial disease burden and is likely to be cost-effective in 77% of MICs not eligible for support from Gavi, on the basis of a willingness-to-pay threshold set at 0·5 times the national GDP per capita. Recently prequalified vaccines might provide good value for money if offered at the prices indicated by manufacturers. Rotasiil was estimated to be the most cost-effective product in all countries, followed closely by Rotavac. This pattern remains sensitive to assumptions about inputs, particularly costs of delivery and vaccine prices.^38^

An early rotavirus vaccine, RotaShield (Wyeth-Ayerst, Philadelphia, PA, USA), was withdrawn from the market in the USA after its association with one excess case of intussusception per fewer than 10 000 fully vaccinated individuals.^39^ However, evidence from self-controlled case-series studies in the past few years suggests that the current generation of rotavirus vaccines are associated with a much lower level of risk and, in some settings, no elevated risk. For example, studies of Rotarix and Rotavac in low-income settings have reported no elevated risk.^40^, ^41^ In MICs, where the potential risk of intussusception might have been an obstacle to introduction, our analysis of hospital admissions suggests that the benefits of vaccination would greatly outweigh the risks. Our benefit–risk estimates with age restrictions (1000:1) are consistent with a ratio of 841:1 estimated by Desai and colleagues, in 14 Latin American countries.^42^ It will be important to continue to monitor the benefits and risks of rotavirus vaccination as more post-licensure surveillance data emerge, particularly from MICs with a similar epidemiological profile to the countries included in our analysis.

This analysis has several limitations. We did not consider indirect (herd immunity) benefits of rotavirus vaccination. Post-licensure data from LMICs have provided mixed evidence on the scale of these possible effects.^14^ Therefore, our results are probably conservative because they might not capture the full extent of benefits from rotavirus vaccines. We assumed that most model parameters related to efficacy and intussusception were the same for all three vaccines due to no head-to-head data on these factors. If subtle or important differences in the effect, safety, or both of the products emerge from post-licensure studies, these differences will need to be considered when comparing products. We did not undertake a head-to-head comparison of the three products because of a scarcity of product-specific data that would fully account for differences in product characteristics and their impact on cost. For example, data on the cost of delivery and international transportation remain scarce and did not allow differentiation between products. However, one can reasonably assume that a product with a larger volume per dose might lead to higher transportation costs and potentially higher costs of delivery because of the larger cold chain space required for storage and transportation. More data are needed to account fully for the cost implications of different product characteristics, and only a few studies provide such comparisons.^43^ We included indirect costs as a measure of loss of income for caregivers in the societal perspective. Although this has the potential to overstate societal health-care costs, we explored wide ranges for this parameter in the probabilistic analysis (−50% to +50% of base input). Although our analysis assumed a small increased risk of intussusception with all three vaccines, we should note that studies in the past few years of Rotarix and Rotavac in low-income settings have reported no elevated risk,^40^, ^41^ thus our benefit–risk ratio might be conservative.

As a multi-country analysis, results might differ slightly from country-focused analyses drawing on specific local data. Countries should interpret the results with caution and are encouraged to develop their own country-specific studies. Similarly, the vaccine prices in this analysis were estimates, so countries will need to confirm vaccine prices directly with manufacturers of products of interest. The results of this study should not dictate product choice decisions in a particular country, but they can inform pricing discussions. In some cases, in which third-party funding has been provided to a manufacturer to offset the cost of vaccine development, existing agreements related to that funding can provide countries access to better prices than they might be able to negotiate individually. In the regions of the Americas, the Pan American Health Organization Revolving Fund has successfully negotiated lower prices with manufacturers through pooled procurement for a group of countries. This kind of pooled procurement mechanism does not exist in many regions in which MICs are found.

On the basis of research on cost-effectiveness thresholds in the past decade,^32^, ^33^ we elected to use the relatively stringent threshold of 0·5 GDP per capita in this study. Individual countries should interpret our results in light of their own benchmarks and policies. Additionally, decisions on rotavirus vaccine introduction and product selection should involve careful weighing of costs against various other considerations (eg, cold chain capacity, transport systems, and non-economic factors such as local epidemiology, ease of use, feasibility, and acceptability).

Many MICs have not yet introduced rotavirus vaccines, at least partly due to concerns about cost and cost-effectiveness in the absence of international vaccine support. However, recent developments in product availability and market prices have improved the accessibility and value for money of rotavirus vaccines. This analysis provides strong evidence for the cost-effectiveness and public health benefits of rotavirus vaccines in most MICs not eligible for funding from Gavi. Use of rotavirus vaccines in these countries has a high probability to be both beneficial and cost-effective if introduced in routine immunisation. In countries already implementing rotavirus vaccination, more recently prequalified products might be considered because they typically improve affordability and value for money.

## Data sharing

All data used in this study are available either in the manuscript, in publications referenced in the study, or through the model that is available online.

## Declaration of interests

FD and RB report grants from the Bill & Melinda Gates Foundation during the course of the study. All other authors declare no competing interests.

## Footnotes

* Countries using rotavirus vaccine as part of their national immunisation programme as of July 9, 2020.^7^, ^10^
