# Supplementary material for: Evaluating the potential economic and health impact of rotavirus vaccination in 63 middle-income countries not eligible for Gavi funding: a modelling study
Source: Lancet Glob Health. 2021 Apr 20;9(7):e942–56. doi: 10.1016/S2214-109X(21)00167-4 (PMC8205857; doi:10.1016/S2214-109X(21)00167-4)
Supplement: Supplementary appendix [file mmc1.pdf]

THE LANCET
Global Health

Supplementary appendix

This appendix formed part of the original submission and has been peer reviewed. We post it as supplied by the authors.

Supplement to: Debellut F, Clark A, Pecenka C, et al. Evaluating the potential economic and health impact of rotavirus vaccination in 63 middle-income countries not eligible for Gavi funding: a modelling study. *Lancet Glob Health* 2021; published online April 20. http://dx.doi.org/10.1016/S2214-109X(21)00167-4.

Supplementary materials

Table S1: RVGE disease burden data inputs.

The table shows pre-vaccine disease event rate per 100,000 under five children per year. Low and high bounds are used for the probabilistic sensitivity analysis.

	RVGE deaths			Non-severe RVGE cases			Severe RVGE cases			Non-severe RVGE clinic visits			Severe RVGE clinic visits			Severe RVGE hospital admissions		
	Base	Low	High	Base	Low	High	Base	Low	High	Base	Low	High	Base	Low	High	Base	Low	High
Africa Region																		
Algeria	5.16	1.62	16.42	7,462	5,256	10,784	2,538	1,744	3,216	739	260	2,669	2,513	1,727	3,184	503	173	955
Botswana	33.07	19.62	55.73	7,216	5,338	9,939	2,784	1,662	4,061	707	262	2,435	2,729	1,629	3,979	546	163	1,194
Cabo Verde	7.27	5.19	10.17	7,419	5,332	10,523	2,581	1,668	3,477	724	260	2,568	2,519	1,628	3,393	504	163	1,018
Equatorial Guinea	38.04	24.67	58.65	7,078	5,413	9,317	2,922	1,587	4,683	399	153	1,314	1,648	895	2,641	330	90	792
Gabon	22.89	19.57	26.78	7,199	5,414	9,694	2,801	1,586	4,306	586	220	1,973	2,280	1,291	3,505	456	129	1,052
Mauritius	2.27	2.10	2.45	7,545	5,249	11,002	2,455	1,751	2,998	744	259	2,712	2,421	1,726	2,956	484	173	887
Namibia	29.63	26.68	32.90	7,929	5,902	10,599	2,071	1,098	3,401	714	266	2,385	1,864	988	3,061	373	99	918
South Africa	32.22	20.80	49.92	7,993	6,087	10,156	2,007	913	3,844	636	242	2,021	1,598	726	3,060	320	73	918
Eswatini	54.28	36.55	80.61	7,314	5,334	10,228	2,686	1,666	3,772	714	260	2,496	2,622	1,626	3,682	524	163	1,104
Americas Region																		
Argentina	1.00	0.93	1.07	8,244	5,793	11,725	1,756	1,207	2,275	783	275	2,785	1,668	1,147	2,162	334	115	649
Belize	3.20	2.86	3.58	8,306	5,897	11,682	1,694	1,103	2,318	811	288	2,850	1,653	1,076	2,262	331	108	679
Brazil	6.91	2.94	16.25	8,158	5,813	11,453	1,842	1,187	2,547	804	287	2,823	1,817	1,171	2,511	363	117	753
Colombia	3.03	2.15	4.27	8,247	5,908	11,496	1,753	1,092	2,504	775	278	2,702	1,647	1,027	2,354	329	103	706
Costa Rica	0.70	0.48	1.01	8,119	5,706	11,564	1,881	1,294	2,436	812	285	2,891	1,881	1,294	2,436	376	129	731
Dominican Republic	9.26	6.77	12.67	8,249	5,883	11,555	1,751	1,117	2,445	767	274	2,686	1,629	1,039	2,274	326	104	682
Ecuador	6.18	3.84	9.96	8,411	6,190	11,224	1,589	810	2,776	738	272	2,464	1,395	711	2,437	279	71	731
El Salvador	9.32	5.56	15.60	7,943	5,691	11,123	2,057	1,309	2,877	739	265	2,586	1,913	1,218	2,676	383	122	803
Grenada	1.50	0.66	3.41	8,283	5,846	11,723	1,717	1,154	2,277	828	292	2,931	1,717	1,154	2,277	343	115	683
Guatemala	18.86	14.20	25.04	8,327	6,046	11,387	1,673	954	2,613	786	285	2,687	1,579	901	2,466	316	90	740
Jamaica	1.33	0.96	1.85	8,287	5,860	11,707	1,713	1,140	2,293	802	284	2,833	1,658	1,104	2,220	332	110	666
Mexico	3.13	1.27	7.72	8,722	6,567	10,641	1,278	433	3,359	836	315	2,549	1,225	415	3,218	245	42	965
Panama	8.76	5.68	13.51	8,061	5,706	11,414	1,939	1,294	2,586	777	275	2,751	1,869	1,248	2,493	374	125	748
Paraguay	6.12	2.54	14.75	8,501	6,296	11,157	1,499	704	2,843	814	302	2,672	1,436	674	2,723	287	67	817
Peru	5.18	2.12	12.61	8,589	6,425	10,917	1,411	575	3,083	833	312	2,647	1,369	558	2,991	274	56	897
Saint Lucia	0.11	0.01	1.13	8,843	6,567	11,246	1,157	433	2,754	874	324	2,778	1,143	428	2,721	229	43	816
Saint Vincent and the Grenadines	0.14	0.01	2.32	8,849	6,567	11,274	1,151	433	2,726	873	324	2,779	1,135	427	2,688	227	43	806
Suriname	2.59	1.14	5.88	8,398	6,069	11,545	1,602	931	2,455	781	282	2,684	1,489	866	2,283	298	87	685
Venezuela	9.26	4.90	17.50	7,983	5,686	11,243	2,017	1,314	2,757	715	255	2,518	1,807	1,178	2,471	361	118	741
Eastern Mediterranean Region																		
Egypt	10.26	7.54	13.96	8,312	6,119	11,201	1,688	881	2,799	800	294	2,694	1,624	848	2,692	325	85	808
Iran	3.42	2.52	4.64	7,650	5,922	9,566	2,350	1,078	4,434	756	293	2,363	2,322	1,065	4,381	464	106	1,314

Iraq	12.00	7.01	20.57	7,919	5,697	11,126	2,081	1,303	2,874	668	240	2,348	1,756	1,100	2,426	351	110	728
Jordan	2.37	1.31	4.30	7,789	5,692	10,765	2,211	1,308	3,235	765	279	2,643	2,171	1,284	3,177	434	128	953
Lebanon	1.34	1.16	1.55	7,968	5,682	11,289	2,032	1,318	2,711	797	284	2,822	2,032	1,318	2,711	406	132	813
Libya	1.39	0.81	2.38	8,256	6,048	11,235	1,744	952	2,765	809	296	2,753	1,709	933	2,710	342	93	813
Morocco	15.19	9.57	24.11	7,794	5,674	10,823	2,206	1,326	3,177	772	281	2,679	2,184	1,313	3,145	437	131	943
State of Palestine	2.03	0.84	4.88	8,062	5,778	11,340	1,938	1,222	2,660	766	274	2,693	1,841	1,161	2,527	368	116	758
Syrian Arab Republic	4.28	0.47	39.36	7,966	5,683	11,282	2,034	1,317	2,718	578	206	2,048	1,476	956	1,973	295	96	592
Tunisia	1.59	0.73	3.48	8,156	5,869	11,398	1,844	1,131	2,602	803	289	2,804	1,815	1,113	2,561	363	111	768
Europe Region																		
Albania	0.89	0.35	2.29	8,566	6,150	11,793	1,434	850	2,207	848	304	2,919	1,420	841	2,185	284	84	655
Belarus	0.15	0.09	0.25	8,455	5,934	11,957	1,545	1,066	2,043	845	297	2,989	1,545	1,066	2,043	309	107	613
Bosnia and Herzegovina	0.24	0.12	0.48	8,430	5,887	11,979	1,570	1,113	2,021	843	294	2,995	1,570	1,113	2,021	314	111	606
Bulgaria	0.84	0.65	1.08	8,359	5,810	11,936	1,641	1,190	2,064	836	291	2,984	1,641	1,190	2,064	328	119	619
Croatia	0.07	0.02	0.32	9,001	6,676	11,186	999	324	2,814	900	334	2,796	999	324	2,814	200	32	844
Kazakhstan	2.86	1.02	8.04	8,675	6,149	12,117	1,325	851	1,883	854	303	2,981	1,304	837	1,853	261	84	556
Kosovo	0.06	0.02	0.15	8,956	6,678	11,141	1,044	322	2,859	838	313	2,607	977	302	2,676	195	30	803
Montenegro	0.06	0.02	0.15	8,997	6,676	11,167	1,003	324	2,833	900	334	2,792	1,003	324	2,833	201	32	850
Romania	0.90	0.42	1.96	8,195	5,891	11,319	1,805	1,109	2,681	819	295	2,830	1,805	1,109	2,681	361	111	804
Russian Federation	0.59	0.53	0.65	8,251	5,861	11,550	1,749	1,139	2,450	825	293	2,888	1,749	1,139	2,450	350	114	735
Serbia	0.06	0.02	0.23	9,001	6,676	11,188	999	324	2,812	900	334	2,797	999	324	2,812	200	32	843
North Macedonia	0.94	0.56	1.58	8,407	5,857	11,974	1,593	1,143	2,026	841	293	2,993	1,593	1,143	2,026	319	114	608
Turkey	0.80	0.71	0.92	8,568	6,168	11,751	1,432	832	2,249	838	302	2,873	1,401	813	2,200	280	81	660
Turkmenistan	10.67	2.05	55.52	8,858	6,421	11,943	1,142	579	2,057	873	317	2,944	1,126	570	2,028	225	57	608
South East Asia Region																		
Maldives	0.84	0.70	1.01	8,250	5,899	11,581	1,750	1,101	2,419	825	295	2,895	1,750	1,101	2,419	350	110	726
Thailand	2.38	1.74	3.25	7,767	5,832	10,288	2,233	1,168	3,712	777	292	2,572	2,233	1,168	3,712	447	117	1,114
Western Pacific Region																		
China	2.00	1.47	2.71	8,200	5,847	11,622	1,800	1,153	2,378	812	289	2,876	1,782	1,141	2,354	356	114	706
Fiji	8.28	6.14	11.17	8,271	5,914	11,669	1,729	1,086	2,331	819	293	2,888	1,712	1,075	2,308	342	107	692
Malaysia	0.80	0.56	1.15	8,336	6,243	10,906	1,664	757	3,094	834	312	2,726	1,664	757	3,094	333	76	928
Micronesia	8.60	1.34	55.36	8,336	5,985	11,692	1,664	1,015	2,308	797	286	2,794	1,591	971	2,207	318	97	662
Philippines	18.91	12.88	27.75	8,139	6,008	11,043	1,861	992	2,957	708	261	2,402	1,619	863	2,572	324	86	772
Samoa	2.03	0.77	5.30	8,337	5,986	11,693	1,663	1,014	2,307	745	268	2,613	1,486	906	2,063	297	91	619
Tonga	2.10	0.84	5.28	8,361	6,008	11,708	1,639	992	2,292	711	255	2,488	1,393	843	1,948	279	84	584
Tuvalu	1.30	0.04	47.46	8,290	5,947	11,650	1,710	1,053	2,350	821	294	2,883	1,693	1,042	2,326	339	104	698
Vanuatu	17.56	7.35	41.95	8,337	5,982	11,702	1,663	1,018	2,298	629	226	2,206	1,254	767	1,733	251	77	520

Table S2: Intussusception disease burden data inputs.

The table shows rate per 100,000 under five children per year in absence of vaccination. Low and high bounds are used for the probabilistic sensitivity analysis.

	Intussusception cases			Intussusception hospital admissions		
	Base	Low	High	Base	Low	High
Africa Region						
Algeria	8.5	3.3	13.7	8.4	3.3	13.5
Botswana	8.6	3.3	13.8	8.4	3.3	13.5
Cabo Verde	8.6	3.3	13.9	8.4	3.3	13.5
Equatorial Guinea	14.9	5.8	24.0	8.4	3.3	13.5
Gabon	10.3	4.0	16.6	8.4	3.3	13.5
Mauritius	8.5	3.3	13.7	8.4	3.3	13.5
Namibia	9.3	3.6	15.0	8.4	3.3	13.5
South Africa	17.0	4.1	17.0	13.5	3.3	13.5
Eswatini	8.6	3.3	13.9	8.4	3.3	13.5
Americas Region						
Argentina	8.8	3.4	14.3	8.4	3.3	13.5
Belize	8.6	3.3	13.9	8.4	3.3	13.5
Brazil	1.2	1.2	34.2	1.2	1.2	33.7
Colombia	12.4	1.3	35.9	11.7	1.2	33.7
Costa Rica	5.7	1.2	33.7	5.7	1.2	33.7
Dominican Republic	12.8	1.3	36.3	11.9	1.2	33.7
Ecuador	12.5	1.4	38.4	11.0	1.2	33.7
El Salvador	11.8	1.3	36.3	11.0	1.2	33.7
Grenada	11.0	1.2	33.7	11.0	1.2	33.7
Guatemala	11.6	1.3	35.7	11.0	1.2	33.7
Jamaica	11.3	1.3	34.8	11.0	1.2	33.7
Mexico	29.5	1.3	35.2	28.3	1.2	33.7
Panama	7.5	1.3	23.2	7.2	1.2	22.3
Paraguay	11.5	1.3	35.2	11.0	1.2	33.7
Peru	8.3	1.3	34.8	8.1	1.2	33.7
Saint Lucia	11.1	1.2	34.1	11.0	1.2	33.7
Saint Vincent and the Grenadines	11.1	1.2	34.2	11.0	1.2	33.7
Suriname	11.8	1.3	36.3	11.0	1.2	33.7
Venezuela	12.3	1.4	37.6	11.0	1.2	33.7
Eastern Mediterranean Region						
Egypt	19.6	13.5	24.1	18.9	13.0	23.2
Iran	19.1	13.2	23.5	18.9	13.0	23.2
Iraq	22.4	15.4	27.5	18.9	13.0	23.2
Jordan	19.2	13.2	23.6	18.9	13.0	23.2
Lebanon	18.9	13.0	23.2	18.9	13.0	23.2
Libya	19.3	13.3	23.7	18.9	13.0	23.2
Morocco	19.1	13.1	23.4	18.9	13.0	23.2
State of Palestine	19.9	13.7	24.4	18.9	13.0	23.2
Syrian Arab Republic	26.0	17.9	32.0	18.9	13.0	23.2

Tunisia	13.2	13.2	23.6	13.0	13.0	23.2
Europe Region						
Albania	14.4	4.3	49.8	14.3	4.2	49.3
Belarus	8.4	3.3	13.5	8.4	3.3	13.5
Bosnia and Herzegovina	8.4	3.3	13.5	8.4	3.3	13.5
Bulgaria	14.3	4.2	49.3	14.3	4.2	49.3
Croatia	14.3	4.2	49.3	14.3	4.2	49.3
Kazakhstan	14.5	4.3	50.1	14.3	4.2	49.3
Kosovo	15.3	4.5	52.7	14.3	4.2	49.3
Montenegro	14.3	4.2	49.3	14.3	4.2	49.3
Romania	14.3	4.2	49.3	14.3	4.2	49.3
Russian Federation	14.3	4.2	49.3	14.3	4.2	49.3
Serbia	14.3	4.2	49.3	14.3	4.2	49.3
North Macedonia	14.3	4.2	49.3	14.3	4.2	49.3
Turkey	14.6	4.3	50.4	14.3	4.2	49.3
Turkmenistan	14.5	4.3	50.0	14.3	4.2	49.3
South East Asia Region						
Maldives	18.5	4.3	61.0	18.5	4.3	61.0
Thailand	8.1	4.3	61.0	8.1	4.3	61.0
Western Pacific Region						
China	51.5	4.8	198.1	50.9	4.8	196.1
Fiji	52.3	4.8	198.1	51.8	4.8	196.1
Malaysia	4.8	4.8	196.1	4.8	4.8	196.1
Micronesia	54.2	5.0	205.1	51.8	4.8	196.1
Philippines	59.6	5.5	225.4	51.8	4.8	196.1
Samoa	58.0	5.4	219.4	51.8	4.8	196.1
Tonga	61.0	5.6	230.7	51.8	4.8	196.1
Tuvalu	52.3	4.8	198.1	51.8	4.8	196.1
Vanuatu	68.7	6.4	260.1	51.8	4.8	196.1

Table S3: Healthcare unit costs, 2018 US$.

	Unit cost per RVGE hospitalization Government perspective	Unit cost per RVGE hospitalization Societal perspective	Unit cost per RVGE outpatient visit Government perspective	Unit cost per RVGE outpatient visit Societal perspective	Unit cost per intussusception case Government perspective	Unit cost per intussusception case Societal perspective
Albania	203	259	11	19	105	175
Algeria	131	169	9	14	269	342
Argentina	595	749	23	39	215	281
Belarus	272	345	13	21	1291	1619
Belize	239	302	13	21	545	672
Bosnia and Herzegovina	214	275	12	20	231	334
Botswana	441	555	18	30	490	620
Brazil	81	122	6	14	384	482
Bulgaria	369	470	16	28	121	229
Cabo Verde	101	132	8	14	255	323
China	226	299	13	24	69	177
Colombia	246	314	21	33	285	359
Costa Rica	481	613	21	36	348	442
Croatia	723	913	26	45	549	796
Dominican Republic	235	306	13	22	352	442
Ecuador	286	362	14	23	284	357
Egypt	76	98	7	11	243	316
El Salvador	28	45	4	9	254	318
Equatorial Guinea	885	1096	28	44	490	625
Fiji	142	188	10	18	199	302
Gabon	398	502	17	28	368	471
Grenada	430	547	20	34	545	679
Guatemala	138	179	10	16	283	354
Iran	210	269	10	18	560	705
Iraq	70	100	7	13	272	377
Jamaica	243	307	14	22	545	672
Jordan	111	145	8	14	182	256
Kazakhstan	267	349	13	24	231	367
Kosovo	75	102	7	12	120	184
Lebanon	542	676	22	35	248	367
Libya	401	503	16	27	302	424
Malaysia	350	453	15	28	199	351
Maldives	313	405	16	29	199	341
Mauritius	445	567	18	32	490	627
Mexico	501	631	20	33	361	454
Micronesia	148	189	11	17	104	160
Montenegro	377	479	17	28	231	359
Morocco	130	166	9	15	87	133
Namibia	200	258	12	20	490	614
State of Palestine	75	99	7	12	141	198
Panama	523	674	22	40	365	466
Paraguay	141	186	10	17	297	372
Peru	274	350	14	24	247	314
Philippines	75	99	7	12	70	115

	Unit cost per RVGE hospitalization Government perspective	Unit cost per RVGE hospitalization Societal perspective	Unit cost per RVGE outpatient visit Government perspective	Unit cost per RVGE outpatient visit Societal perspective	Unit cost per intussusception case Government perspective	Unit cost per intussusception case Societal perspective
Romania	441	566	18	33	128	264
Russian Federation	603	759	22	37	557	775
Saint Lucia	481	610	21	36	545	678
Saint Vincent and the Grenadines	375	472	17	29	545	675
Samoa	142	182	10	17	199	282
Serbia	280	357	14	23	359	499
South Africa	394	493	18	29	281	362
Suriname	178	232	11	19	545	673
Eswatini	111	145	8	14	340	427
Syrian Arab Republic	11	19	4	6	141	198
North Macedonia	262	332	13	22	231	335
Thailand	200	261	11	20	65	149
Tonga	161	207	11	18	199	284
Tunisia	161	204	10	15	250	331
Turkey	349	447	15	26	194	318
Turkmenistan	286	364	14	24	70	147
Tuvalu	286	355	14	22	199	278
Vanuatu	217	270	14	21	104	156
Venezuela	8	54	3	16	545	674

Intussusception treatment costs calculations

Building on a recent systematic review (https://www.ncbi.nlm.nih.gov/pubmed/30879038) we searched for available data on intussusception management and cost for our list of focus countries. We were able to find information on treatment patterns for a limited number of countries (N=9), and on length of hospitalization for an even more limited number of countries (N=4). We were not able to locate any study with data on intussusception costs for our list of countries but one study with cost information from Nigeria (https://www.ncbi.nlm.nih.gov/pmc/articles/PMC4955470/).

Two main patterns of intussusception management were identified: surgical procedures and non-operative management. We were able to find region specific proportions of different types of treatment used in managing intussusception from another literature review (https://www.ncbi.nlm.nih.gov/pmc/articles/PMC3718796/). Those proportions mostly aligned with information we collected from the 9 countries for which published studies are available. Table 1 shows the proportion of surgical and non-operative procedures applied in our calculations.

Table 1. Regional proportion of different intussusception treatment patterns

	Proportion of surgery procedures	Proportion of non-operative management
Africa Region	77%	23%
Americas Region*	89%	11%
Eastern Mediterranean Region	29%	71%
Europe Region	20%	80%
South East Asia Region**	13%	87%
Western Pacific Region**	13%	87%

*using Jiang reported data for "Central and South America"
**using Jiang reported data for "Asia"

Similarly to what has been done by Verguet et al 2015 (https://www.thelancet.com/journals/langlo/article/PIIS2214-109X(15)70086-0/fulltext#seccestitle160) we used available data on caesarean unit cost from Gibbons et al. 2010 https://www.who.int/healthsystems/topics/financing/healthreport/30C-sectioncosts.pdf as a proxy for surgical unit cost, covering costs related to human resources, supplies, equipment, and drugs for the procedure itself, along with the pre and post hospitalization.

We estimated the cost of non-operative management by applying the cost differential between surgical procedures and non-operative management as reported in the only study we were able to identify reporting unit cost for these interventions (https://www.ncbi.nlm.nih.gov/pmc/articles/PMC4955470/).

Table 2 shows unit cost per surgery and non-operative management per country (2018 US$).

Table 2. Country surgery and non-operative management unit costs, 2018 US$.

	Surgery unit cost	Non-operative management unit cost
Albania	270	64
Algeria	327	77
Argentina	235	55
Belarus	3,322	783
Belize	595	140
Bosnia and Herzegovina	595	140
Botswana	595	140
Brazil	420	99
Bulgaria	311	73
Cabo Verde	309	73
China	206	49
Colombia	311	73
Costa Rica	380	90
Croatia	1,413	333
Dominican Republic	384	91
Ecuador	310	73
Egypt	532	125
El Salvador	277	65
Equatorial Guinea	595	140
Fiji	595	140
Gabon	447	105
Grenada	595	140
Guatemala	309	73
Iran	1,225	289
Iraq	595	140
Jamaica	595	140
Jordan	399	94
Kazakhstan	595	140
Kosovo	309	73
Lebanon	543	128
Libya	660	156
Malaysia	595	140
Maldives	595	140
Mauritius	595	140
Mexico	394	93
Micronesia	309	73
Montenegro	595	140
Morocco	191	45
Namibia	595	140
State of Palestine	309	73
Panama	398	94
Paraguay	324	76
Peru	270	64
Philippines	208	49
Romania	330	78
Russian Federation	1,433	338
Saint Lucia	595	140
Saint Vincent and the Grenadines	595	140
Samoa	595	140
Serbia	923	218
South Africa	341	81
Suriname	595	140
Eswatini	412	97
Syrian Arab Republic	309	73
North Macedonia	595	140
Thailand	194	46
Tonga	595	140
Tunisia	546	129
Turkey	498	118
Turkmenistan	180	42
Tuvalu	595	140
Vanuatu	309	73
Venezuela	595	140

Finally, we calculated a weighted direct medical cost of intussusception treatment by multiplying the regional proportions for surgical procedures and non-operative management with their respective unit cost. This direct medical cost is used for our government perspective.

To estimate intussusception treatment cost from the societal perspective, we added direct non-medical costs and indirect costs. Direct non-medical costs, covering for households' expenses in terms of travel to the health facility, food and other incidentals, are estimated as a percentage of direct medical costs. We assumed 21% based on cost of diarrhea data (Baral et al. 2020).

Indirect costs are estimated by multiplying a length of stay of 7 days for surgery and 4 days for non-operative management by their respective regional proportions and by each country GDP per capita divided by 365. We added indirect costs to our direct medical costs to estimate our societal perspective costs.

Table 3 below summarizes modelled intussusception costs for our focus countries (in 2018 USD).

Table 3. Modelled costs per intussusception case for 63 middle-income countries, 2018 USD.

	Total costs Societal perspective	Direct medical costs Government perspective	Direct non-medical costs	Indirect costs
Albania	175	105	22	48
Algeria	342	269	57	16
Argentina	281	215	45	20
Belarus	1,619	1,291	271	57
Belize	672	545	114	12
Bosnia and Herzegovina	334	231	49	55
Botswana	620	490	103	26
Brazil	482	384	81	17
Bulgaria	229	121	25	83
Cabo Verde	323	255	53	15
China	177	69	14	94
Colombia	359	285	60	14
Costa Rica	442	348	73	21
Croatia	796	549	115	132
Dominican Republic	442	352	74	16
Ecuador	357	284	60	14
Egypt	316	243	51	22
El Salvador	318	254	53	11
Equatorial Guinea	625	490	103	31
Fiji	302	199	42	61
Gabon	471	368	77	25
Grenada	679	545	114	19
Guatemala	354	283	59	12
Iran	705	560	118	27
Iraq	377	272	57	47
Jamaica	672	545	114	13
Jordan	256	182	38	35
Kazakhstan	367	231	49	87
Kosovo	184	120	25	39
Lebanon	367	248	52	66
Libya	424	302	63	58
Malaysia	351	199	42	109
Maldives	341	199	42	99
Mauritius	627	490	103	34
Mexico	454	361	76	18
Micronesia	160	104	22	35
Montenegro	359	231	49	79
Morocco	133	87	18	27
Namibia	614	490	103	20
State of Palestine	198	141	30	27
Panama	466	365	77	25
Paraguay	372	297	62	13
Peru	314	247	52	15
Philippines	115	70	15	30
Romania	264	128	27	109
Russian Federation	775	557	117	100
Saint Lucia	678	545	114	19
Saint Vincent and the Grenadines	675	545	114	15
Samoa	282	199	42	41
Serbia	499	359	75	65
South Africa	362	281	59	21
Suriname	673	545	114	14
Eswatini	427	340	71	16
Syrian Arab Republic	198	141	30	27
North Macedonia	335	231	49	55
Thailand	149	65	14	70
Tonga	284	199	42	43
Tunisia	331	250	52	29
Turkey	318	194	41	84
Turkmenistan	147	70	15	62
Tuvalu	278	199	42	36
Vanuatu	156	104	22	31
Venezuela	674	545	114	15

Table S4. Vaccine parameters

Parameter	Base case input	Low input	High input	Source
Number of doses for a full course				
ROTARIX	2	-	-	
ROTAVAC	3	-	-	
ROTASIIL	3	-	-	
Vaccine price per dose*				
ROTARIX	$10.25	$7.18	$13.33	Informed by manufacturers, cross checked with WHO vaccine purchase database
ROTAVAC	$1.25	$0.88	$1.63	
ROTASIIL	$1	$0.70	$1.30	
Number of doses per vial				
ROTARIX	1	-	-	Gavi the Vaccine Alliance. Detailed product profiles. Available from: https://www.gavi.org/about/market-shaping/detailed-product-profiles/ Accessed July 14, 2020.
ROTAVAC	5	-	-	
ROTASIIL	2	-	-	
Vaccine wastage				
ROTARIX	5%	2%	10%	Gavi the Vaccine Alliance. Detailed product profiles. Available from: https://www.gavi.org/about/market-shaping/detailed-product-profiles/ Accessed July 14, 2020.
ROTAVAC	23%	15%	30%	
ROTASIIL	10%	5%	15%	Assumption
International handling and transportation				
Handling cost as a percentage of vaccine price	3.5%	1.4%	5%	UNICEF. Handling Fees. Supplies and Logistics. Available from: https://www.unicef.org/supply/index_62330.html Accessed July 14, 2020.
International freight/transport cost as a percentage of vaccine price	6%	2%	15%	Assumption
Commodities				
Safety box/bag (100 doses volume)	$0.42	-	-	UNICEF. Auto-disable AD syringes and Safety Boxes price data. Available from: https://www.unicef.org/supply/files/Auto-disable_AD_syringes_and_Safety_Boxes_price_data.pdf Accessed July 14, 2020.
Vaccine administration				
Incremental cost of delivery per dose	$2.05	$1	$2.5	[31]

*Each of the manufacturers (GSK, Bharat Biotech, and Serum Institute) provided information with varying levels of detail which was used to calculate a base price and upper and lower bounds for each product. If a price range was provided, we identified a midpoint price and rounded to the nearest 25 cents, rounding down in the event the midpoint was nearly equidistant between two potential price points. Upper and lower bounds for uncertainty analysis were calculated as +/- 30% of the base price, roughly aligning with the potential price ranges provided by manufacturers. If a single price was provided, we rounded to the nearest 25 cents and created a range using the methods described above.

Table S5: parameters included in the probabilistic analysis, base values, ranges and distributions.

Parameter	Base case (95% CI)	Probability distribution	Source
**Population projections for the 2015 birth cohort**<br>Population by single age/year between birth and 5·0 years	Country-specific	Beta-PERT (mid = UNPOP medium variant, range = UNPOP low/high variant)	United Nations Population Division. World Population Prospects - 2017 revision. Available from: https://population.un.org/wpp/ Accessed July 14, 2020.
**Disease burden estimates**			
RVGE incidence rate <5 years<br>Non-severe RVGE visits<br>Severe RVGE visits<br>Severe RVGE hospitalizations	Regional-specific	Beta-PERT (mid = regional value, range = reported uncertainty range for RVGE incidence, proportion range for treatment seeking rate:<br>10%-30% for hospitalizations, 5%-25% for clinic visits)	[14]
Severe RVGE mortality rate <5 years	Country-specific	Beta-PERT (mid = Log transformed mean of 3 sources of country estimates, range = 95% CI)	
**Age distribution of RVGE deaths**			
Log Logistic scale parameter	Country-specific	Beta-PERT (mid = best fit for country/U5MR stratum, range = 95% CI for country/U5MR stratum)	[22]
**Age distribution of background intussusception cases**	Country-specific	Beta-PERT (mid = best fit for country/region, range = 95% CI for country/region)	[14]
**Disability weights for calcualting DALYs**<br>Percentage of healthy time lost whilst living with the disease			
Non Severe RVGE	18·8% (12·5%-26·4%)	Beta-PERT (mid = Salomon, GBD 2013, moderate diarhea, range = Salomon, GBD 2013, moderate diarrhea, 95% uncetainty interval)	
Severe RVGE	24·7% (16·4%-34·8%)	Beta-PERT (mid = Salomon, GBD 2013, severe diarhea, range = Salomon, GBD 2013, severe diarhea, 95% uncetainty interval)	[23]
Non Severe RVGE	5 days (3-7)	Beta-PERT (mid = CDC, range = CDC)	U.S CDC. The Pink Book, Epidemiology and Prevention of Vaccine-Preventable Diseases, Rotavirus. Available from: https://www.cdc.gov/vaccines/pubs/pinkbook/rota.html Accessed July 14, 2020.
Severe RVGE	5 days (3-7))	Beta-PERT (mid = CDC, range = CDC)	
**Vaccine coverage**			
Doses with DTP1, DTP2, DTP3	Country-specific	Beta-PERT (mid = WUENIC 2015, range = WUENIC 2015 +/-10%)	[24]
**Vaccine timeliness**			
Log Logistic scale parameter	Country-specific	Beta-PERT (mid = best fit for country or schedule stratum, range = median age +/-10%)	[25]
**Initial efficacy against RVGE mortality (2wks after dose administered)**			
Low mortality	99·6% (99·4%-99·7%)	Beta-PERT	
Medium mortality	91·4% (89·8%-92·7%)	Beta-PERT	[26]

High mortality	78·9% (75·5%-82·3%)	Beta-PERT	
**Mean duration of vaccine efficacy in months**			
Low mortality	176·8 (114·7%-268·0%)	Beta-PERT	
Medium mortality	121·9 (81·3%-182·4%)		
High mortality	13·2 (9·2%-20·5%)		[26]
**Relative efficacy of 1 dose versus 2/3 doses**			
Low- and middle-income countries (LMICs)	0·63 (0·51%-0·79%)	Beta-PERT	[14]
**Relative risk of vaccine-related intussusception vs background rate**			
Dose 1, 1-7 days	6.26 (4.25-9.22)	Lognormal (m = ln(6.26), sd = 0.198)	[14]
Dose 1, 8-21 days	1.69 (1.05-2.72)	Lognormal (m = ln(1.69), sd = 0.243)	
Dose 2, 1-7 days	1.82 (1.41-2.32)	Lognormal (m = ln(1.82), sd = 0.129)	
Dose 2, 8-21 days	1.37 (1.03-1.84)	Lognormal (m = ln(1.37), sd = 0.149)	
**Vaccine price**			
ROTARIX	$10.25 ($7.18-$13.33)	Beta-PERT	Informed by vaccine manufacturers
ROTAVAC	$1.25 ($0.88-$1.63)		
ROTASIIL	$1 ($0.70-$1.30)		
**Incremental health system costs**	$2.05 ($1-$2.5)	Beta-PERT (mid = ICAN data, range based on authors assumptions)	[31]
**International handling**	3·5% (1·4%-5%)	Beta-PERT (mid = UNICEF, range based on min and max from UNICEF)	UNICEF. Handling Fees. Supplies and Logistics. Available from: https://www.unicef.org/supply/index_62330.html Accessed July 14, 2020.
**International delivery**	6% (2%-15%)	Beta-PERT (mid = literature data, range based on assumptions)	Assumption
**Vaccine wastage**			
ROTARIX	5% (2%-10%)	Beta-PERT	Gavi the Vaccine Alliance. Detailed product profiles. Available from: https://www.gavi.org/about/market-shaping/detailed-product-profiles/ Accessed July 14, 2020.
ROTAVAC	25% (15%-30%)	Beta-PERT (mid = India MoH, low range based on assumtpion, high range based on Gavi recommendations)	
ROTASIIL	10% (5%-15%)	Beta-PERT	
**Treatment costs**			
Cost per clinic visit	Country-specific (+/-50%)	Beta-PERT (mid = modeled data from Baral et al., range = modeled data from Baral et al. +/- 50%)	[28]
Cost per hospitalization	Country-specific (+/-50%)		

Table S6: Vaccine impact results. 10 cohorts vaccinated, 2020-2029.

Table shows vaccine impact results compared to no vaccination, assuming a 2-dose age-restricted schedule in all countries.

	Averted rotavirus burden					Averted healthcare costs	
	Cases	Visits	Hospitalizations	Deaths	Disability Adjusted Life Years**	Government perspective**	Societal perspective**
All 63 MICs	74,554,428	20,390,938	2,935,230	36,051	1,109,885	$804,933,891	$1,152,021,893
Africa Region	2,720,675	755,143	116,372	6,277	165,014	$41,829,707	$56,169,960
Algeria	551,563	195,075	31,216	245	7,794	5,049,413	7,083,558
Botswana*	87,392	32,630	5,348	257	6,797	2,577,700	3,447,644
Cabo Verde	26,208	9,245	1,486	15	460	197,953	280,904
Equatorial Guinea	33,600	7,471	1,239	112	2,819	1,133,002	1,467,300
Gabon	80,814	25,167	4,131	161	4,264	1,802,998	2,423,994
Mauritius*	30,922	10,646	1,688	6	234	805,648	1,108,855
Namibia*	116,270	32,600	4,919	282	7,380	1,197,570	1,676,477
South Africa*	1,740,464	422,929	63,197	4,962	129,253	28,617,198	38,041,447
Eswatini*	53,442	19,381	3,148	237	6,013	448,225	639,781
Americas Region	19,432,531	5,044,143	707,371	9,717	299,808	$211,104,938	$305,725,756
Argentina*	1,814,394	483,253	69,086	160	8,458	45,203,880	61,118,518
Belize	19,839	5,309	749	6	196	216,214	293,327
Brazil*	6,301,518	1,795,283	260,879	3,691	111,964	27,278,497	49,212,925
Colombia*	1,708,440	449,656	64,236	463	16,216	21,925,641	30,390,319
Costa Rica*	230,711	67,548	9,884	13	895	5,295,406	7,312,347
Dominican Republic*	509,575	132,639	18,944	442	12,710	5,304,467	7,594,394
Ecuador*	762,099	176,432	24,338	418	12,750	8,183,376	11,199,929
El Salvador*	276,249	79,682	11,998	207	6,051	591,303	1,063,249
Grenada	5,402	1,494	212	1	35	103,389	142,858
Guatemala*	564,033	144,847	20,356	857	23,766	3,687,254	5,232,881
Jamaica	116,082	31,023	4,397	14	647	1,289,898	1,760,997
Mexico*	4,654,322	1,036,154	131,171	1,393	47,007	74,829,301	101,696,788
Panama*	205,585	59,158	8,742	166	4,854	5,058,497	7,122,517
Paraguay*	210,357	51,314	6,925	123	3,712	1,278,490	1,876,331
Peru*	897,299	213,934	28,199	407	12,897	9,367,459	13,017,802
Saint Lucia	5,212	1,133	137	0	14	78,345	107,975
Saint Vincent and the Grenadines	3,865	836	101	0	10	45,495	62,054
Suriname	15,729	3,875	536	4	131	118,076	169,616
Venezuela*	1,131,820	310,574	46,481	1,352	37,494	1,249,949	6,350,929
Eastern Mediterranean Region	16,488,865	4,681,427	693,739	10,957	324,131	$111,758,254	$157,163,774
Egypt	7,352,100	1,935,020	272,833	6,304	180,936	28,908,134	40,990,401
Iran	3,641,717	1,219,392	191,020	1,009	35,437	45,784,670	63,386,536
Iraq*	1,861,019	490,846	74,172	1,911	53,308	7,391,322	12,053,014
Jordan*	279,879	89,417	13,763	58	2,202	1,969,122	2,829,881
Lebanon	312,309	96,096	14,415	44	1,881	8,546,033	11,309,922
Libya*	195,357	53,440	7,621	23	1,064	3,426,566	4,601,342
Morocco*	1,049,557	337,549	51,921	1,231	35,263	8,693,382	11,928,096
State of Palestine*	441,066	125,046	18,477	83	3,182	1,950,732	2,834,383
Syrian Arab Republic	838,165	187,340	28,109	223	7,777	865,398	1,488,181
Tunisia	517,696	147,280	21,409	70	3,081	4,222,896	5,742,016
Europe Region	8,455,011	2,158,601	293,792	752	39,315	$144,519,344	$198,784,479
Albania*	77,751	19,096	2,533	7	356	635,734	884,729
Belarus	170,444	44,191	6,033	2	456	1,885,508	2,593,358
Bosnia and Herzegovina	53,475	14,000	1,923	1	154	495,859	695,177
Bulgaria*	183,346	49,290	6,879	13	752	2,855,619	3,957,051

	Averted rotavirus burden					Averted healthcare costs	
	Cases	Visits	Hospitalizations	Deaths	Disability Adjusted Life Years**	Government perspective**	Societal perspective**
Croatia	107,713	21,972	2,489	1	265	2,035,270	2,802,664
Kazakhstan	744,609	173,719	22,322	149	5,605	7,114,325	10,383,149
Kosovo	25,319	4,941	571	0	61	67,690	104,464
Montenegro	20,189	4,125	468	0	49	209,997	292,547
Romania	551,527	157,312	22,702	43	2,413	11,059,876	15,475,206
Russian Federation	2,756,773	771,014	110,075	122	9,642	71,709,156	96,564,366
Serbia	236,167	48,163	5,455	1	574	1,875,249	2,638,432
North Macedonia*	32,005	8,452	1,167	3	156	356,445	489,888
Turkey	3,300,942	800,361	106,117	206	13,151	42,439,420	59,427,144
Turkmenistan*	194,750	41,966	5,059	205	5,681	1,779,197	2,476,305
South East Asia Region	2,215,139	724,401	111,753	432	16,508	$26,269,047	$37,678,917
Maldives	20,413	5,711	815	1	82	298,611	425,753
Thailand*	2,194,726	718,690	110,937	430	16,426	25,970,436	37,253,164
Western Pacific Region	25,242,207	7,027,223	1,012,204	7,915	265,108	$269,452,600	$396,499,007
China	21,091,497	5,945,170	857,136	3,580	143,827	233,754,250	345,325,838
Fiji*	46,787	12,861	1,829	38	1,075	337,443	497,697
Malaysia	1,745,170	473,242	66,383	129	7,397	25,979,073	37,108,077
Micronesia*	4,088	1,060	149	3	92	29,423	40,424
Philippines	2,327,877	588,985	85,880	4,142	112,053	9,156,791	13,261,963
Samoa	6,912	1,675	235	1	48	43,842	61,453
Tonga	5,787	1,322	184	1	41	38,452	53,460
Tuvalu	425	116	16	0	2	5,507	7,240
Vanuatu	13,664	2,792	392	21	572	107,819	142,854

*Countries using rotavirus vaccine as part of their national immunization program, as of July 2020. [7,10] ** discounted values

Table S7: Vaccine impact results. 10 cohorts vaccinated, 2020-2029.

Table shows vaccine impact results compared to no vaccination, assuming a 3-dose age-unrestricted schedule in all countries.

	Averted rotavirus burden					Averted healthcare costs	
	Cases	Visits	Hospitalizations	Deaths	Disability Adjusted Life Years**	Government perspective**	Societal perspective**
All 63 MICs	112,598,716	31,054,423	4,475,501	46,594	1,473,278	$1,264,648,110	$1,813,784,376
Africa Region	4,315,471	1,280,630	199,652	7,924	210,820	$59,782,436	$80,861,479
Algeria	1,741,846	616,049	98,579	772	24,531	15,926,165	22,342,711
Botswana*	110,468	41,247	6,760	324	8,589	3,257,408	4,356,750
Cabo Verde	30,900	10,900	1,752	18	542	233,339	331,120
Equatorial Guinea	39,765	8,842	1,466	133	3,333	1,340,571	1,736,114
Gabon	100,869	31,412	5,156	201	5,318	2,249,551	3,024,359
Mauritius*	36,253	12,482	1,979	7	275	944,469	1,299,928
Namibia*	126,573	35,489	5,355	307	8,036	1,303,936	1,825,372
South Africa*	2,070,985	503,244	75,199	5,905	153,691	34,042,051	45,252,935
Eswatini*	57,812	20,966	3,405	257	6,505	484,945	692,191
Americas Region	23,244,368	6,014,087	841,785	11,624	358,459	$254,273,110	$367,475,879
Argentina*	2,122,382	565,284	80,813	187	9,890	52,868,929	71,482,173
Belize	24,782	6,631	936	7	244	270,000	366,298
Brazil*	7,383,070	2,103,414	305,654	4,325	131,159	31,955,127	57,650,269
Colombia*	2,004,191	527,497	75,356	543	19,017	25,716,228	35,644,405
Costa Rica*	240,418	70,390	10,300	14	933	5,517,842	7,619,505
Dominican Republic*	596,551	155,279	22,178	518	14,876	6,208,652	8,888,961
Ecuador*	898,294	207,962	28,687	493	15,022	9,643,967	13,198,957
El Salvador*	288,477	83,209	12,529	216	6,320	617,575	1,110,471
Grenada	6,193	1,712	243	1	40	118,484	163,716
Guatemala*	769,765	197,681	27,781	1,170	32,410	5,028,459	7,136,328
Jamaica	136,731	36,541	5,179	17	762	1,518,931	2,073,695
Mexico*	5,876,459	1,308,228	165,614	1,759	59,269	94,408,772	128,307,766
Panama*	239,237	68,842	10,173	193	5,647	5,885,671	8,287,212
Paraguay*	269,714	65,793	8,879	158	4,758	1,638,770	2,405,078
Peru*	1,063,667	253,599	33,427	482	15,286	11,102,091	15,428,346
Saint Lucia	6,112	1,329	161	0	16	91,865	126,608
Saint Vincent and the Grenadines	4,549	984	119	0	12	53,535	73,021
Suriname	28,226	6,954	962	7	235	211,489	303,818
Venezuela*	1,285,549	352,757	52,794	1,535	42,562	1,416,723	7,209,251
Eastern Mediterranean Region	18,771,266	5,360,629	796,815	12,299	364,453	$130,046,570	$182,946,295
Egypt	7,725,462	2,033,286	286,688	6,624	190,133	30,377,879	43,074,336
Iran	4,302,280	1,440,575	225,669	1,192	41,852	54,072,765	74,862,003
Iraq*	2,273,730	599,699	90,621	2,335	65,089	9,026,105	14,719,103
Jordan*	575,219	183,774	28,286	120	4,518	4,042,365	5,809,471
Lebanon	353,156	108,665	16,300	49	2,126	9,661,346	12,785,894
Libya*	248,111	67,871	9,679	29	1,351	4,350,383	5,841,882
Morocco*	1,304,577	419,566	64,536	1,530	43,814	10,801,552	14,820,643
State of Palestine*	459,486	130,268	19,248	87	3,317	2,032,523	2,953,221
Syrian Arab Republic	953,300	213,074	31,970	254	8,825	983,716	1,691,798
Tunisia	575,945	163,851	23,817	78	3,428	4,697,936	6,387,944
Europe Region	13,004,623	3,378,618	464,244	1,067	58,113	$242,200,787	$331,356,177
Albania*	91,820	22,551	2,991	8	421	750,638	1,044,635
Belarus	363,814	94,326	12,876	5	973	4,020,870	5,530,489
Bosnia and Herzegovina	79,169	20,726	2,847	2	227	733,549	1,028,410
Bulgaria*	211,129	56,759	7,921	14	866	3,287,403	4,555,360
Croatia	123,622	25,217	2,857	1	304	2,335,181	3,215,651
Kazakhstan	1,083,438	252,769	32,480	217	8,150	10,344,400	15,097,281
Kosovo	32,695	6,381	738	0	79	87,391	134,868
Montenegro	25,023	5,113	580	0	60	260,189	362,469
Romania	638,280	182,057	26,273	50	2,791	12,791,838	17,898,559

	Averted rotavirus burden					Averted healthcare costs	
	Cases	Visits	Hospitalizations	Deaths	Disability Adjusted Life Years**	Government perspective**	Societal perspective**
Russian Federation	5,856,777	1,638,022	233,854	259	20,466	152,213,160	204,971,486
Serbia	285,950	58,315	6,604	2	695	2,269,762	3,193,506
North Macedonia*	73,339	19,368	2,674	7	357	815,978	1,121,449
Turkey	3,891,860	943,637	125,114	242	15,501	50,028,028	70,053,171
Turkmenistan*	247,705	53,377	6,434	260	7,223	2,262,400	3,148,842
South East Asia Region	2,555,907	835,836	128,943	498	19,041	$30,301,487	$43,462,629
Maldives	23,632	6,611	944	1	95	345,581	492,717
Thailand	2,532,275	829,225	127,999	497	18,946	29,955,906	42,969,912
Western Pacific Region	50,707,082	14,184,623	2,044,061	13,181	462,390	$548,043,721	$807,681,918
China	45,675,082	12,874,673	1,856,187	7,754	310,995	505,868,966	747,212,515
Fiji*	54,407	14,956	2,127	44	1,250	392,282	578,577
Malaysia	2,016,856	546,915	76,718	149	8,546	30,014,842	42,872,586
Micronesia*	5,012	1,299	182	4	112	36,060	49,543
Philippines	2,923,845	739,774	107,866	5,202	140,675	11,497,759	16,652,303
Samoa	7,770	1,883	264	1	54	49,278	69,073
Tonga	6,668	1,523	212	1	48	44,288	61,575
Tuvalu	525	143	20	0	3	6,798	8,936
Vanuatu	16,917	3,457	485	26	708	133,449	176,811

*Countries using rotavirus vaccine as part of their national immunization program, as of July 2020. [7,10] ** discounted values

Table S8: Vaccine impact results. 10 cohorts vaccinated, 2020-2029.

Table shows vaccine impact results compared to no vaccination, assuming a 2-dose age-unrestricted schedule in all countries.

	Averted rotavirus burden					Averted healthcare costs	
	Cases	Visits	Hospitalizations	Deaths	Disability Adjusted Life Years**	Government perspective**	Societal perspective**
All 63 MICs	108,778,112	30,011,083	4,324,461	44,077	1,399,012	$1,226,374,394	$1,759,003,775
Africa Region	3,956,606	1,173,256	182,888	7,281	193,632	$54,872,879	$74,216,536
Algeria	1,586,514	561,112	89,788	704	22,323	14,505,064	20,349,344
Botswana*	99,886	37,296	6,113	293	7,766	2,945,417	3,939,476
Cabo Verde	29,814	10,517	1,690	17	523	225,112	319,445
Equatorial Guinea	36,872	8,198	1,360	123	3,090	1,243,036	1,609,801
Gabon	92,492	28,803	4,728	184	4,875	2,062,692	2,773,145
Mauritius*	35,397	12,187	1,933	7	268	922,006	1,269,011
Namibia*	117,017	32,809	4,951	283	7,427	1,205,234	1,687,209
South Africa*	1,905,170	462,952	69,178	5,432	141,347	31,316,067	41,629,286
Eswatini*	53,445	19,382	3,148	237	6,013	448,251	639,818
Americas Region	22,293,021	5,770,203	807,844	11,096	342,393	$244,254,100	$353,068,271
Argentina*	2,048,868	545,704	78,014	181	9,546	51,033,815	69,000,991
Belize	23,926	6,402	904	7	236	260,651	353,616
Brazil*	7,126,410	2,030,293	295,029	4,175	126,589	30,841,704	55,641,712
Colombia*	1,933,683	508,940	72,705	524	18,344	24,809,422	34,387,557
Costa Rica*	237,550	69,551	10,177	14	922	5,452,190	7,528,848
Dominican Republic*	575,850	149,890	21,408	500	14,358	5,992,636	8,579,714
Ecuador*	866,721	200,652	27,679	475	14,491	9,304,107	12,733,835
El Salvador*	278,173	80,237	12,081	208	6,093	595,411	1,070,639
Grenada	6,124	1,693	240	1	40	117,165	161,894
Guatemala*	688,110	176,711	24,834	1,045	28,978	4,495,605	6,380,227
Jamaica	130,951	34,997	4,960	16	730	1,454,559	1,985,818
Mexico*	5,674,892	1,263,355	159,933	1,698	57,227	91,168,612	123,904,503
Panama*	231,057	66,488	9,825	186	5,454	5,683,988	8,003,240
Paraguay*	239,773	58,490	7,893	141	4,230	1,456,763	2,138,012
Peru*	945,379	225,397	29,710	428	13,587	9,868,414	13,713,996
Saint Lucia	5,899	1,283	156	0	15	88,656	122,186
Saint Vincent and the Grenadines	4,390	950	115	0	12	51,652	70,452
Suriname	27,216	6,705	928	6	227	203,891	292,906
Venezuela*	1,248,049	342,467	51,254	1,491	41,316	1,374,860	6,998,124
Eastern Mediterranean Region	17,929,457	5,120,456	760,950	11,671	346,040	$124,385,652	$174,899,255
Egypt	7,465,054	1,964,749	277,025	6,401	183,710	29,351,091	41,618,573
Iran	4,151,256	1,390,006	217,747	1,150	40,377	52,166,214	72,222,923
Iraq*	2,069,458	545,822	82,480	2,125	59,201	8,212,975	13,394,022
Jordan*	561,861	179,506	27,629	117	4,412	3,948,107	5,674,014
Lebanon	349,144	107,430	16,115	49	2,102	9,551,989	12,641,165
Libya*	222,133	60,764	8,666	26	1,209	3,894,860	5,230,155
Morocco*	1,190,532	382,888	58,895	1,396	39,986	9,857,572	13,525,325
State of Palestine*	443,488	125,733	18,578	84	3,200	1,961,425	2,849,922
Syrian Arab Republic	926,582	207,103	31,074	247	8,571	955,969	1,644,129
Tunisia	549,949	156,455	22,742	74	3,272	4,485,450	6,099,027
Europe Region	12,681,277	3,299,087	453,649	1,014	55,990	$237,636,915	$324,999,077
Albania*	88,588	21,757	2,886	7	406	724,152	1,007,773
Belarus	361,323	93,680	12,788	5	966	3,993,340	5,492,628
Bosnia and Herzegovina	78,254	20,487	2,814	2	225	725,115	1,016,585
Bulgaria*	209,034	56,196	7,843	14	857	3,254,881	4,510,293
Croatia	122,172	24,921	2,823	1	301	2,307,867	3,178,037
Kazakhstan	1,007,719	235,103	30,210	202	7,581	9,622,788	14,044,058
Kosovo	29,033	5,666	655	0	70	77,595	119,750
Montenegro	24,801	5,068	575	0	60	257,888	359,262

	Averted rotavirus burden					Averted healthcare costs	
	Cases	Visits	Hospitalizations	Deaths	Disability Adjusted Life Years**	Government perspective**	Societal perspective**
Romania	621,920	177,391	25,600	49	2,720	12,468,799	17,446,532
Russian Federation	5,803,780	1,623,200	231,738	257	20,281	150,839,651	203,121,878
Serbia	283,175	57,749	6,540	2	688	2,247,778	3,162,574
North Macedonia*	72,602	19,173	2,647	7	353	807,804	1,110,215
Turkey	3,755,023	910,459	120,715	234	14,954	48,264,664	67,583,830
Turkmenistan*	223,853	48,238	5,815	235	6,528	2,044,595	2,845,660
South East Asia Region	2,525,751	825,974	127,422	492	18,817	$29,945,216	$42,951,582
Maldives	23,354	6,534	933	1	94	341,526	486,935
Thailand*	2,502,397	819,441	126,489	491	18,723	29,603,690	42,464,648
Western Pacific Region	49,392,000	13,822,106	1,991,708	12,522	442,139	$535,279,632	$788,869,053
China	44,654,297	12,586,939	1,814,703	7,580	303,987	494,498,285	730,410,234
Fiji*	52,926	14,549	2,069	43	1,215	381,557	562,759
Malaysia	1,996,641	541,434	75,949	147	8,461	29,714,573	42,443,679
Micronesia*	4,511	1,169	164	4	101	32,457	44,592
Philippines	2,653,709	671,425	97,901	4,721	127,631	10,434,204	15,111,772
Samoa	7,590	1,839	258	1	53	48,131	67,466
Tonga	6,485	1,481	207	1	46	43,066	59,876
Tuvalu	477	130	18	0	3	6,177	8,121
Vanuatu	15,363	3,140	440	24	642	121,182	160,556

*Countries using rotavirus vaccine as part of their national immunization program, as of July 2020. [7,10] ** discounted values

Table S9: Cost per DALY averted. 10 cohorts vaccinated, 2020-2029.

Results for age-restricted schedules. Each vaccine compared to no vaccination.

Note: the incremental cost-effectiveness of the second and third ranked options would be less favorable if vaccines were compared to each other rather than each being compared to ‘no vaccination’

	Cost per DALY averted Government perspective**			Cost per DALY averted as a share of GDP per capita Government perspective**			Cost per DALY averted Societal perspective**			Cost per DALY averted as a share of GDP per capita Societal perspective**		
	ROTARIX	ROTAVAC	ROTASIIL	ROTARIX	ROTAVAC	ROTASIIL	ROTARIX	ROTAVAC	ROTASIIL	ROTARIX	ROTAVAC	ROTASIIL
Africa Region												
Algeria	$8,332	$2,781	$2,278	2.03	0.68	0.55	$8,071	$2,520	$2,017	1.96	0.61	0.49
Botswana*	$1,240	$211	$125	0.15	0.03	0.02	$1,112	$83	Cost saving	0.13	0.01	Cost saving
Cabo Verde	$3,992	$1,186	$949	1.10	0.33	0.26	$3,812	$1,005	$768	1.05	0.28	0.21
Equatorial Guinea	$1,017	$74	$4	0.10	0.01	0.00	$899	Cost saving	Cost saving	0.09	Cost saving	Cost saving
Gabon	$2,007	$476	$345	0.25	0.06	0.04	$1,862	$331	$199	0.23	0.04	0.03
Mauritius*	$7,615	$1,009	$358	0.68	0.09	0.03	$6,322	Cost saving	Cost saving	0.56	Cost saving	Cost saving
Namibia*	$1,913	$622	$507	0.32	0.10	0.09	$1,849	$557	$442	0.31	0.09	0.07
South Africa*	$1,281	$333	$252	0.20	0.05	0.04	$1,208	$260	$179	0.19	0.04	0.03
Eswatini*	$1,007	$333	$273	0.24	0.08	0.07	$975	$301	$241	0.24	0.07	0.06
Americas Region												
Argentina*	$11,413	$712	Cost saving	0.98	0.06	Cost saving	$9,531	Cost saving	Cost saving	0.82	Cost saving	Cost saving
Belize	$6,837	$1,804	$1,378	1.40	0.37	0.28	$6,443	$1,410	$984	1.32	0.29	0.20
Brazil*	$4,147	$1,347	$1,114	0.46	0.15	0.12	$3,951	$1,151	$918	0.44	0.13	0.10
Colombia*	$6,903	$1,656	$1,215	1.04	0.25	0.18	$6,381	$1,134	$693	0.96	0.17	0.10
Costa Rica*	$10,151	$299	Cost saving	0.84	0.02	Cost saving	$7,899	Cost saving	Cost saving	0.66	Cost saving	Cost saving
Dominican Republic*	$2,733	$722	$555	0.34	0.09	0.07	$2,553	$542	$375	0.32	0.07	0.05
Ecuador*	$4,047	$1,071	$820	0.64	0.17	0.13	$3,810	$834	$583	0.60	0.13	0.09
El Salvador*	$3,482	$1,262	$1,063	0.86	0.31	0.26	$3,404	$1,184	$985	0.84	0.29	0.24
Grenada	$6,738	$800	$251	0.63	0.08	0.02	$5,614	Cost saving	Cost saving	0.53	Cost saving	Cost saving
Guatemala*	$3,087	$917	$760	0.68	0.20	0.17	$3,022	$852	$695	0.66	0.19	0.15
Jamaica	$11,978	$3,063	$2,322	2.24	0.57	0.43	$11,250	$2,335	$1,594	2.10	0.44	0.30
Mexico*	$6,212	$1,230	$816	0.64	0.13	0.08	$5,641	$658	$245	0.58	0.07	0.03
Panama*	$2,267	$145	Cost saving	0.15	0.01	Cost saving	$1,841	Cost saving	Cost saving	0.12	Cost saving	Cost saving
Paraguay*	$6,887	$2,129	$1,767	1.18	0.37	0.30	$6,726	$1,968	$1,606	1.16	0.34	0.28
Peru*	$8,221	$2,256	$1,819	1.18	0.32	0.26	$7,938	$1,973	$1,536	1.14	0.28	0.22
Saint Lucia	$24,522	$5,206	$3,595	2.32	0.49	0.34	$22,333	$3,017	$1,406	2.11	0.29	0.13
Saint Vincent and the Grenadines	$25,454	$6,452	$4,854	3.46	0.88	0.66	$23,835	$4,833	$3,235	3.24	0.66	0.44
Suriname	$8,457	$2,758	$2,222	1.36	0.44	0.36	$8,064	$2,366	$1,830	1.29	0.38	0.29
Venezuela*	$2,684	$1,245	$1,128	0.16	0.07	0.07	$2,548	$1,109	$992	0.15	0.07	0.06
Eastern Mediterranean Region												
Egypt	$2,936	$1,048	$871	1.15	0.41	0.34	$2,869	$982	$805	1.13	0.39	0.32
Iran	$6,669	$1,625	$1,198	1.20	0.29	0.22	$6,172	$1,128	$701	1.11	0.20	0.13
Iraq*	$4,087	$1,308	$1,096	0.70	0.22	0.19	$4,000	$1,220	$1,008	0.69	0.21	0.17
Jordan*	$9,497	$3,318	$2,701	2.24	0.78	0.64	$9,106	$2,927	$2,310	2.15	0.69	0.54
Lebanon	$6,374	Cost saving	Cost saving	0.77	Cost saving	Cost saving	$4,905	Cost saving	Cost saving	0.59	Cost saving	Cost saving

	Cost per DALY averted Government perspective**			Cost per DALY averted as a share of GDP per capita Government perspective**			Cost per DALY averted Societal perspective**			Cost per DALY averted as a share of GDP per capita Societal perspective**		
	ROTARIX	ROTAVAC	ROTASIIL	ROTARIX	ROTAVAC	ROTASIIL	ROTARIX	ROTAVAC	ROTASIIL	ROTARIX	ROTAVAC	ROTASIIL
Libya*	$19,151	$4,497	$3,366	2.64	0.62	0.46	$18,047	$3,394	$2,263	2.49	0.47	0.31
Morocco*	$3,502	$1,140	$937	1.07	0.35	0.29	$3,410	$1,048	$845	1.04	0.32	0.26
State of Palestine*	$10,171	$3,604	$2,986	3.18	1.13	0.93	$9,893	$3,326	$2,708	3.09	1.04	0.85
Syrian Arab Republic	$7,601	$2,605	$2,207	N/A	N/A	N/A	$7,521	$2,525	$2,127	N/A	N/A	N/A
Tunisia	$11,335	$3,324	$2,636	3.29	0.96	0.76	$10,842	$2,831	$2,143	3.14	0.82	0.62
Europe Region												
Albania*	$15,424	$4,520	$3,597	2.93	0.86	0.68	$14,726	$3,822	$2,898	2.79	0.73	0.55
Belarus	$19,188	$5,471	$4,063	3.05	0.87	0.65	$17,636	$3,919	$2,512	2.80	0.62	0.40
Bosnia and Herzegovina	$19,010	$5,025	$3,816	3.13	0.83	0.63	$17,714	$3,729	$2,520	2.92	0.61	0.42
Bulgaria*	$11,781	$2,355	$1,453	1.27	0.25	0.16	$10,316	$890	Cost saving	1.11	0.10	Cost saving
Croatia	$18,343	$1,901	$498	1.23	0.13	0.03	$15,451	Cost saving	Cost saving	1.04	Cost saving	Cost saving
Kazakhstan	$9,050	$2,297	$1,775	0.92	0.23	0.18	$8,466	$1,714	$1,192	0.86	0.17	0.12
Kosovo	$52,482	$17,319	$14,619	12.20	4.03	3.40	$51,881	$16,719	$14,018	12.06	3.89	3.26
Montenegro	$22,385	$6,276	$4,722	2.53	0.71	0.53	$20,683	$4,574	$3,020	2.34	0.52	0.34
Romania	$9,977	Cost saving	Cost saving	0.81	Cost saving	Cost saving	$8,147	Cost saving	Cost saving	0.66	Cost saving	Cost saving
Russian Federation	$10,418	Cost saving	Cost saving	0.91	Cost saving	Cost saving	$7,840	Cost saving	Cost saving	0.68	Cost saving	Cost saving
Serbia	$23,145	$7,316	$5,765	3.19	1.01	0.80	$21,816	$5,986	$4,435	3.01	0.83	0.61
North Macedonia*	$10,590	$2,998	$2,223	1.74	0.49	0.37	$9,734	$2,141	$1,367	1.60	0.35	0.22
Turkey	$16,402	$3,946	$2,895	1.75	0.42	0.31	$15,111	$2,655	$1,603	1.61	0.28	0.17
Turkmenistan*	$4,068	$1,297	$1,061	0.58	0.19	0.15	$3,945	$1,174	$938	0.57	0.17	0.13
South East Asia Region												
Maldives	$11,972	$2,185	$1,333	1.16	0.21	0.13	$10,425	$638	Cost saving	1.01	0.06	Cost saving
Thailand*	$6,738	$1,505	$1,052	0.93	0.21	0.14	$6,051	$818	$365	0.83	0.11	0.05
Western Pacific Region												
China	$9,944	$3,078	$2,389	1.02	0.32	0.24	$9,169	$2,302	$1,613	0.94	0.24	0.17
Fiji*	$3,077	$1,048	$848	0.49	0.17	0.14	$2,928	$898	$699	0.47	0.14	0.11
Malaysia	$11,203	$2,432	$1,561	0.98	0.21	0.14	$9,698	$928	$57	0.85	0.08	0.00
Micronesia*	$5,396	$1,562	$1,286	1.51	0.44	0.36	$5,276	$1,442	$1,166	1.48	0.40	0.33
Philippines	$2,640	$936	$787	0.85	0.30	0.25	$2,604	$900	$751	0.84	0.29	0.24
Samoa	$9,877	$2,934	$2,372	2.36	0.70	0.57	$9,513	$2,570	$2,008	2.27	0.61	0.48
Tonga	$10,007	$3,380	$2,748	2.29	0.77	0.63	$9,644	$3,017	$2,385	2.21	0.69	0.55
Tuvalu	$22,826	$6,820	$5,470	6.17	1.84	1.48	$22,073	$6,067	$4,717	5.96	1.64	1.27
Vanuatu	$3,475	$1,655	$1,487	1.11	0.53	0.48	$3,414	$1,594	$1,426	1.09	0.51	0.46

*Countries using rotavirus vaccine as part of their national immunization program, as of July 2020. [7,10] ** discounted values

Table S10: Cost per DALY averted. 10 cohorts vaccinated, 2020-2029.

Results for age-unrestricted schedules. Each vaccine compared to no vaccination.

Note: the incremental cost-effectiveness of the second and third ranked options would be less favorable if vaccines were compared to each other rather than each being compared to 'no vaccination'

	Cost per DALY averted Government perspective**			Cost per DALY averted as a share of GDP per capita Government perspective**			Cost per DALY averted Societal perspective**			Cost per DALY averted as a share of GDP per capita Societal perspective**		
	ROTARIX	ROTAVAC	ROTASIIL	ROTARIX	ROTAVAC	ROTASIIL	ROTARIX	ROTAVAC	ROTASIIL	ROTARIX	ROTAVAC	ROTASIIL
Africa Region												
Algeria	$8,482	$2,746	$2,248	2.06	0.67	0.55	$8,220	$2,485	$1,987	2.00	0.60	0.48
Botswana*	$1,242	$223	$135	0.15	0.03	0.02	$1,114	$95	$7	0.13	0.01	0.00
Cabo Verde	$4,042	$1,351	$1,090	1.11	0.37	0.30	$3,861	$1,171	$910	1.06	0.32	0.25
Equatorial Guinea	$1,016	$76	$6	0.10	0.01	0.00	$898	Cost saving	Cost saving	0.09	Cost saving	Cost saving
Gabon	$2,008	$475	$344	0.25	0.06	0.04	$1,862	$330	$198	0.23	0.04	0.02
Mauritius*	$7,625	$1,021	$367	0.68	0.09	0.03	$6,330	Cost saving	Cost saving	0.56	Cost saving	Cost saving
Namibia*	$1,911	$621	$506	0.32	0.10	0.09	$1,846	$556	$441	0.31	0.09	0.07
South Africa*	$1,321	$353	$269	0.21	0.06	0.04	$1,248	$280	$196	0.20	0.04	0.03
Eswatini*	$1,007	$333	$273	0.24	0.08	0.07	$975	$301	$241	0.24	0.07	0.06
Americas Region												
Argentina*	$11,531	$1,305	$331	0.99	0.11	0.03	$9,648	Cost saving	Cost saving	0.83	Cost saving	Cost saving
Belize	$6,956	$2,109	$1,638	1.42	0.43	0.34	$6,562	$1,715	$1,244	1.34	0.35	0.25
Brazil*	$4,180	$1,504	$1,248	0.47	0.17	0.14	$3,984	$1,308	$1,052	0.45	0.15	0.12
Colombia*	$6,998	$1,983	$1,494	1.05	0.30	0.22	$6,476	$1,461	$972	0.97	0.22	0.15
Costa Rica*	$10,156	$644	Cost saving	0.84	0.05	Cost saving	$7,903	Cost saving	Cost saving	0.66	Cost saving	Cost saving
Dominican Republic*	$2,756	$835	$651	0.34	0.10	0.08	$2,576	$654	$471	0.32	0.08	0.06
Ecuador*	$4,098	$1,246	$969	0.65	0.20	0.15	$3,862	$1,009	$733	0.61	0.16	0.12
El Salvador*	$3,480	$1,326	$1,118	0.86	0.33	0.28	$3,402	$1,248	$1,040	0.84	0.31	0.26
Grenada	$6,809	$1,029	$446	0.64	0.10	0.04	$5,685	Cost saving	Cost saving	0.53	Cost saving	Cost saving
Guatemala*	$3,108	$1,027	$853	0.68	0.23	0.19	$3,043	$962	$788	0.67	0.21	0.17
Jamaica	$12,154	$3,588	$2,770	2.27	0.67	0.52	$11,426	$2,859	$2,042	2.13	0.53	0.38
Mexico*	$6,420	$1,590	$1,124	0.66	0.16	0.12	$5,847	$1,018	$552	0.60	0.11	0.06
Panama*	$2,281	$258	$68	0.15	0.02	0.00	$1,856	Cost saving	Cost saving	0.12	Cost saving	Cost saving
Paraguay*	$6,909	$2,303	$1,915	1.19	0.40	0.33	$6,748	$2,142	$1,754	1.16	0.37	0.30
Peru*	$8,208	$2,506	$2,032	1.18	0.36	0.29	$7,925	$2,223	$1,749	1.14	0.32	0.25
Saint Lucia	$24,773	$6,299	$4,528	2.34	0.60	0.43	$22,584	$4,110	$2,339	2.14	0.39	0.22
Saint Vincent and the Grenadines	$25,757	$7,556	$5,797	3.50	1.03	0.79	$24,138	$5,937	$4,177	3.28	0.81	0.57
Suriname	$9,481	$3,244	$2,637	1.52	0.52	0.42	$9,088	$2,852	$2,244	1.46	0.46	0.36
Venezuela*	$2,673	$1,347	$1,220	0.16	0.08	0.07	$2,537	$1,211	$1,084	0.15	0.07	0.06
Eastern Mediterranean Region												
Egypt	$2,939	$1,077	$896	1.15	0.42	0.35	$2,872	$1,010	$829	1.13	0.40	0.33
Iran	$6,760	$1,925	$1,453	1.22	0.35	0.26	$6,264	$1,428	$957	1.13	0.26	0.17
Iraq*	$4,151	$1,441	$1,209	0.71	0.25	0.21	$4,063	$1,354	$1,122	0.70	0.23	0.19
Jordan*	$10,256	$3,591	$2,934	2.42	0.85	0.69	$9,864	$3,200	$2,543	2.33	0.75	0.60
Lebanon	$6,333	Cost saving	Cost saving	0.77	Cost saving	Cost saving	$4,863	Cost saving	Cost saving	0.59	Cost saving	Cost saving
Libya*	$19,416	$5,146	$3,920	2.68	0.71	0.54	$18,311	$4,042	$2,815	2.53	0.56	0.39
Morocco*	$3,563	$1,193	$982	1.09	0.36	0.30	$3,471	$1,101	$890	1.06	0.34	0.27

	Cost per DALY averted Government perspective**			Cost per DALY averted as a share of GDP per capita Government perspective**			Cost per DALY averted Societal perspective**			Cost per DALY averted as a share of GDP per capita Societal perspective**		
	ROTARIX	ROTAVAC	ROTASIIL	ROTARIX	ROTAVAC	ROTASIIL	ROTARIX	ROTAVAC	ROTASIIL	ROTARIX	ROTAVAC	ROTASIIL
State of Palestine*	$10,160	$3,669	$3,042	3.18	1.15	0.95	$9,883	$3,392	$2,764	3.09	1.06	0.86
Syrian Arab Republic	$7,580	$2,733	$2,316	N/A	N/A	N/A	$7,500	$2,652	$2,236	N/A	N/A	N/A
Tunisia	$11,389	$3,661	$2,924	3.30	1.06	0.85	$10,896	$3,168	$2,431	3.16	0.92	0.71
Europe Region												
Albania*	$15,628	$5,171	$4,152	2.97	0.98	0.79	$14,929	$4,473	$3,453	2.83	0.85	0.66
Belarus	$19,658	$5,651	$4,218	3.13	0.90	0.67	$18,106	$4,100	$2,666	2.88	0.65	0.42
Bosnia and Herzegovina	$19,173	$5,507	$4,227	3.16	0.91	0.70	$17,877	$4,211	$2,931	2.95	0.69	0.48
Bulgaria*	$11,870	$2,590	$1,654	1.28	0.28	0.18	$10,405	$1,125	$189	1.12	0.12	0.02
Croatia	$18,430	$2,872	$1,327	1.24	0.19	0.09	$15,538	Cost saving	Cost saving	1.04	Cost saving	Cost saving
Kazakhstan	$9,288	$2,781	$2,187	0.95	0.28	0.22	$8,705	$2,197	$1,604	0.89	0.22	0.16
Kosovo	$52,839	$18,684	$15,784	12.28	4.34	3.67	$52,237	$18,084	$15,183	12.14	4.20	3.53
Montenegro	$22,465	$6,431	$4,854	2.54	0.73	0.55	$20,763	$4,730	$3,153	2.35	0.53	0.36
Romania	$9,984	$1,133	$295	0.81	0.09	0.02	$8,154	Cost saving	Cost saving	0.66	Cost saving	Cost saving
Russian Federation	$10,905	$90	Cost saving	0.95	0.01	Cost saving	$8,328	Cost saving	Cost saving	0.73	Cost saving	Cost saving
Serbia	$23,307	$7,574	$5,986	3.22	1.05	0.83	$21,978	$6,245	$4,656	3.03	0.86	0.64
North Macedonia*	$10,868	$3,021	$2,243	1.79	0.50	0.37	$10,012	$2,164	$1,386	1.65	0.36	0.23
Turkey	$16,617	$4,680	$3,521	1.77	0.50	0.38	$15,326	$3,388	$2,229	1.64	0.36	0.24
Turkmenistan*	$4,080	$1,331	$1,090	0.59	0.19	0.16	$3,958	$1,208	$967	0.57	0.17	0.14
South East Asia Region												
Maldives	$12,091	$2,801	$1,858	1.17	0.27	0.18	$10,544	$1,254	$311	1.02	0.12	0.03
Thailand*	$6,787	$1,826	$1,326	0.93	0.25	0.18	$6,100	$1,139	$640	0.84	0.16	0.09
Western Pacific Region												
China	$10,202	$3,161	$2,460	1.04	0.32	0.25	$9,426	$2,385	$1,684	0.96	0.24	0.17
Fiji*	$3,126	$1,072	$869	0.50	0.17	0.14	$2,977	$922	$719	0.48	0.15	0.11
Malaysia	$11,314	$2,566	$1,675	0.99	0.23	0.15	$9,809	$1,061	$171	0.86	0.09	0.02
Micronesia*	$5,363	$1,669	$1,378	1.50	0.47	0.39	$5,243	$1,549	$1,257	1.47	0.43	0.35
Philippines	$2,650	$941	$791	0.85	0.30	0.25	$2,613	$904	$754	0.84	0.29	0.24
Samoa	$9,864	$2,974	$2,405	2.36	0.71	0.57	$9,500	$2,610	$2,041	2.27	0.62	0.49
Tonga	$10,136	$3,457	$2,814	2.32	0.79	0.64	$9,773	$3,093	$2,450	2.24	0.71	0.56
Tuvalu	$22,759	$6,823	$5,473	6.15	1.84	1.48	$22,006	$6,070	$4,720	5.95	1.64	1.28
Vanuatu	$3,475	$1,662	$1,492	1.11	0.53	0.48	$3,414	$1,601	$1,431	1.09	0.51	0.46

* Countries using rotavirus vaccine as part of their national immunization program, as of July 2020. [7,10] ** discounted values

Table S11: Cost per DALY averted. 10 cohorts vaccinated, 2020 – 2029.

Results for ROTARIX associated with similar vaccine impact than 3-dose vaccines and age-restricted schedules. Each vaccine compared to no vaccination.

Note: the incremental cost-effectiveness of the second and third ranked options would be less favorable if vaccines were compared to each other rather than each being compared to ‘no vaccination’

	Cost per DALY averted Government perspective**			Cost per DALY averted as a share of GDP per capita Government perspective**			Cost per DALY averted Societal perspective**			Cost per DALY averted as a share of GDP per capita Societal perspective**		
	ROTARIX	ROTAVAC	ROTASIIL	ROTARIX	ROTAVAC	ROTASIIL	ROTARIX	ROTAVAC	ROTASIIL	ROTARIX	ROTAVAC	ROTASIIL
**Africa Region**												
Algeria	$7,681	$2,781	$2,278	1.87	0.68	0.55	$7,440	$2,520	$2,017	1.81	0.61	0.49
Botswana*	$1,140	$211	$125	0.14	0.03	0.02	$1,022	$83	Cost saving	0.12	0.01	Cost saving
Cabo Verde	$3,891	$1,186	$949	1.07	0.33	0.26	$3,715	$1,005	$768	1.02	0.28	0.21
Equatorial Guinea	$950	$74	$4	0.09	0.01	0.00	$839	Cost saving	Cost saving	0.08	Cost saving	Cost saving
Gabon	$1,856	$476	$345	0.23	0.06	0.04	$1,721	$331	$199	0.22	0.04	0.03
Mauritius*	$7,456	$1,009	$358	0.66	0.09	0.03	$6,190	Cost saving	Cost saving	0.55	Cost saving	Cost saving
Namibia*	$1,769	$622	$507	0.30	0.10	0.09	$1,709	$557	$442	0.29	0.09	0.07
South Africa*	$1,181	$333	$252	0.19	0.05	0.04	$1,114	$260	$179	0.17	0.04	0.03
Eswatini*	$931	$333	$273	0.22	0.08	0.07	$901	$301	$241	0.22	0.07	0.06
**Americas Region**												
Argentina*	$11,134	$712	Cost saving	0.95	0.06	Cost saving	$9,299	Cost saving	Cost saving	0.80	Cost saving	Cost saving
Belize	$6,666	$1,804	$1,378	1.36	0.37	0.28	$6,282	$1,410	$984	1.29	0.29	0.20
Brazil*	$4,045	$1,347	$1,114	0.45	0.15	0.12	$3,854	$1,151	$918	0.43	0.13	0.10
Colombia*	$6,734	$1,656	$1,215	1.01	0.25	0.18	$6,225	$1,134	$693	0.93	0.17	0.10
Costa Rica*	$10,055	$299	Cost saving	0.84	0.02	Cost saving	$7,824	Cost saving	Cost saving	0.65	Cost saving	Cost saving
Dominican Republic*	$2,666	$722	$555	0.33	0.09	0.07	$2,490	$542	$375	0.31	0.07	0.05
Ecuador*	$3,945	$1,071	$820	0.62	0.17	0.13	$3,714	$834	$583	0.59	0.13	0.09
El Salvador*	$3,378	$1,262	$1,063	0.83	0.31	0.26	$3,302	$1,184	$985	0.81	0.29	0.24
Grenada	$6,678	$800	$251	0.63	0.08	0.02	$5,564	Cost saving	Cost saving	0.52	Cost saving	Cost saving
Guatemala*	$2,893	$917	$760	0.64	0.20	0.17	$2,832	$852	$695	0.62	0.19	0.15
Jamaica	$11,606	$3,063	$2,322	2.17	0.57	0.43	$10,900	$2,335	$1,594	2.04	0.44	0.30
Mexico*	$6,078	$1,230	$816	0.63	0.13	0.08	$5,519	$658	$245	0.57	0.07	0.03
Panama*	$2,213	$145	Cost saving	0.14	0.01	Cost saving	$1,798	Cost saving	Cost saving	0.12	Cost saving	Cost saving
Paraguay*	$6,322	$2,129	$1,767	1.09	0.37	0.30	$6,175	$1,968	$1,606	1.06	0.34	0.28
Peru*	$7,636	$2,256	$1,819	1.10	0.32	0.26	$7,373	$1,973	$1,536	1.06	0.28	0.22
Saint Lucia	$23,916	$5,206	$3,595	2.26	0.49	0.34	$21,781	$3,017	$1,406	2.06	0.29	0.13
Saint Vincent and the Grenadines	$24,815	$6,452	$4,854	3.37	0.88	0.66	$23,236	$4,833	$3,235	3.16	0.66	0.44
Suriname	$8,199	$2,758	$2,222	1.32	0.44	0.36	$7,819	$2,366	$1,830	1.25	0.38	0.29
Venezuela*	$2,631	$1,245	$1,128	0.16	0.07	0.07	$2,498	$1,109	$992	0.15	0.07	0.06
**Eastern Mediterranean Region**												
Egypt	$2,843	$1,048	$871	1.12	0.41	0.34	$2,779	$982	$805	1.09	0.39	0.32
Iran	$6,500	$1,625	$1,198	1.17	0.29	0.22	$6,016	$1,128	$701	1.08	0.20	0.13
Iraq*	$3,809	$1,308	$1,096	0.65	0.22	0.19	$3,728	$1,220	$1,008	0.64	0.21	0.17
Jordan*	$9,297	$3,318	$2,701	2.19	0.78	0.64	$8,914	$2,927	$2,310	2.10	0.69	0.54
Lebanon	$6,328	Cost saving	Cost saving	0.77	Cost saving	Cost saving	$4,869	Cost saving	Cost saving	0.59	Cost saving	Cost saving
Libya*	$17,619	$4,497	$3,366	2.43	0.62	0.46	$16,603	$3,394	$2,263	2.29	0.47	0.31
Morocco*	$3,234	$1,140	$937	0.99	0.35	0.29	$3,149	$1,048	$845	0.96	0.32	0.26
State of Palestine*	$9,832	$3,604	$2,986	3.07	1.13	0.93	$9,564	$3,326	$2,708	2.99	1.04	0.85

	Cost per DALY averted Government perspective**			Cost per DALY averted as a share of GDP per capita Government perspective**			Cost per DALY averted Societal perspective**			Cost per DALY averted as a share of GDP per capita Societal perspective**		
	ROTARIX	ROTAVAC	ROTASIIL	ROTARIX	ROTAVAC	ROTASIIL	ROTARIX	ROTAVAC	ROTASIIL	ROTARIX	ROTAVAC	ROTASIIL
Syrian Arab Republic	$7,423	$2,605	$2,207	N/A	N/A	N/A	$7,345	$2,525	$2,127	N/A	N/A	N/A
Tunisia	$10,930	$3,324	$2,636	3.17	0.96	0.76	$10,455	$2,831	$2,143	3.03	0.82	0.62
Europe Region												
Albania*	$15,032	$4,520	$3,597	2.85	0.86	0.68	$14,351	$3,822	$2,898	2.72	0.73	0.55
Belarus	$19,082	$5,471	$4,063	3.03	0.87	0.65	$17,539	$3,919	$2,512	2.79	0.62	0.40
Bosnia and Herzegovina	$18,861	$5,025	$3,816	3.11	0.83	0.63	$17,575	$3,729	$2,520	2.90	0.61	0.42
Bulgaria*	$11,691	$2,355	$1,453	1.26	0.25	0.16	$10,238	$890	Cost saving	1.10	0.10	Cost saving
Croatia	$18,200	$1,901	$498	1.22	0.13	0.03	$15,330	Cost saving	Cost saving	1.03	Cost saving	Cost saving
Kazakhstan	$8,489	$2,297	$1,775	0.87	0.23	0.18	$7,942	$1,714	$1,192	0.81	0.17	0.12
Kosovo	$47,998	$17,319	$14,619	11.16	4.03	3.40	$47,448	$16,719	$14,018	11.03	3.89	3.26
Montenegro	$22,212	$6,276	$4,722	2.51	0.71	0.53	$20,524	$4,574	$3,020	2.32	0.52	0.34
Romania	$9,977	Cost saving	Cost saving	0.81	Cost saving	Cost saving	$8,147	Cost saving	Cost saving	0.66	Cost saving	Cost saving
Russian Federation	$10,335	Cost saving	Cost saving	0.90	Cost saving	Cost saving	$7,777	Cost saving	Cost saving	0.68	Cost saving	Cost saving
Serbia	$22,955	$7,316	$5,765	3.17	1.01	0.80	$21,637	$5,986	$4,435	2.99	0.83	0.61
North Macedonia*	$10,495	$2,998	$2,223	1.73	0.49	0.37	$9,647	$2,141	$1,367	1.59	0.35	0.22
Turkey	$15,987	$3,946	$2,895	1.71	0.42	0.31	$14,728	$2,655	$1,603	1.57	0.28	0.17
Turkmenistan*	$3,732	$1,297	$1,061	0.54	0.19	0.15	$3,620	$1,174	$938	0.52	0.17	0.13
South East Asia Region												
Maldives	$11,875	$2,185	$1,333	1.15	0.21	0.13	$10,340	$638	Cost saving	1.00	0.06	Cost saving
Thailand*	$6,684	$1,505	$1,052	0.92	0.21	0.14	$6,003	$818	$365	0.83	0.11	0.05
Western Pacific Region												
China	$9,762	$3,078	$2,389	1.00	0.32	0.24	$9,001	$2,302	$1,613	0.92	0.24	0.17
Fiji*	$2,998	$1,048	$848	0.48	0.17	0.14	$2,852	$898	$699	0.46	0.14	0.11
Malaysia	$11,097	$2,432	$1,561	0.98	0.21	0.14	$9,607	$928	$57	0.84	0.08	0.00
Micronesia*	$5,022	$1,562	$1,286	1.41	0.44	0.36	$4,910	$1,442	$1,166	1.38	0.40	0.33
Philippines	$2,408	$936	$787	0.78	0.30	0.25	$2,375	$900	$751	0.77	0.29	0.24
Samoa	$9,676	$2,934	$2,372	2.31	0.70	0.57	$9,320	$2,570	$2,008	2.23	0.61	0.48
Tonga	$9,754	$3,380	$2,748	2.24	0.77	0.63	$9,400	$3,017	$2,385	2.15	0.69	0.55
Tuvalu	$20,931	$6,820	$5,470	5.66	1.84	1.48	$20,241	$6,067	$4,717	5.47	1.64	1.27
Vanuatu	$3,179	$1,655	$1,487	1.02	0.53	0.48	$3,123	$1,594	$1,426	1.00	0.51	0.46

Results highlighted in light grey remain unchanged
* Countries using rotavirus vaccine as part of their national immunization program, as of July 2020. [7,10]
** discounted values

Table S12: Cost per DALY averted. 10 cohorts vaccinated, 2020 – 2029.

Results for ROTARIX associated with similar vaccine impact than 3-dose vaccines and age-unrestricted schedules. Each vaccine compared to no vaccination.

Note: the incremental cost-effectiveness of the second and third ranked options would be less favorable if vaccines were compared to each other rather than each being compared to ‘no vaccination’

	Cost per DALY averted Government perspective**			Cost per DALY averted as a share of GDP per capita Government perspective**			Cost per DALY averted Societal perspective**			Cost per DALY averted as a share of GDP per capita Societal perspective**		
	ROTARIX	ROTAVAC	ROTASIIL	ROTARIX	ROTAVAC	ROTASIIL	ROTARIX	ROTAVAC	ROTASIIL	ROTARIX	ROTAVAC	ROTASIIL
Africa Region												
Algeria	$7,681	$2,781	$2,278	1.87	0.68	0.55	$7,440	$2,520	$2,017	1.81	0.61	0.49
Botswana*	$1,140	$211	$125	0.14	0.03	0.02	$1,022	$83	Cost saving	0.12	0.01	Cost saving
Cabo Verde	$3,891	$1,186	$949	1.07	0.33	0.26	$3,715	$1,005	$768	1.02	0.28	0.21
Equatorial Guinea	$950	$74	$4	0.09	0.01	0.00	$839	Cost saving	Cost saving	0.08	Cost saving	Cost saving
Gabon	$1,856	$476	$345	0.23	0.06	0.04	$1,721	$331	$199	0.22	0.04	0.03
Mauritius*	$7,456	$1,009	$358	0.66	0.09	0.03	$6,190	Cost saving	Cost saving	0.55	Cost saving	Cost saving
Namibia*	$1,769	$622	$507	0.30	0.10	0.09	$1,709	$557	$442	0.29	0.09	0.07
South Africa*	$1,181	$333	$252	0.19	0.05	0.04	$1,114	$260	$179	0.17	0.04	0.03
Eswatini*	$931	$333	$273	0.22	0.08	0.07	$901	$301	$241	0.22	0.07	0.06
Americas Region												
Argentina*	$11,134	$712	Cost saving	0.95	0.06	Cost saving	$9,299	Cost saving	Cost saving	0.80	Cost saving	Cost saving
Belize	$6,666	$1,804	$1,378	1.36	0.37	0.28	$6,282	$1,410	$984	1.29	0.29	0.20
Brazil*	$4,045	$1,347	$1,114	0.45	0.15	0.12	$3,854	$1,151	$918	0.43	0.13	0.10
Colombia*	$6,734	$1,656	$1,215	1.01	0.25	0.18	$6,225	$1,134	$693	0.93	0.17	0.10
Costa Rica*	$10,055	$299	Cost saving	0.84	0.02	Cost saving	$7,824	Cost saving	Cost saving	0.65	Cost saving	Cost saving
Dominican Republic*	$2,666	$722	$555	0.33	0.09	0.07	$2,490	$542	$375	0.31	0.07	0.05
Ecuador*	$3,945	$1,071	$820	0.62	0.17	0.13	$3,714	$834	$583	0.59	0.13	0.09
El Salvador*	$3,378	$1,262	$1,063	0.83	0.31	0.26	$3,302	$1,184	$985	0.81	0.29	0.24
Grenada	$6,678	$800	$251	0.63	0.08	0.02	$5,564	Cost saving	Cost saving	0.52	Cost saving	Cost saving
Guatemala*	$2,893	$917	$760	0.64	0.20	0.17	$2,832	$852	$695	0.62	0.19	0.15
Jamaica	$11,606	$3,063	$2,322	2.17	0.57	0.43	$10,900	$2,335	$1,594	2.04	0.44	0.30
Mexico*	$6,078	$1,230	$816	0.63	0.13	0.08	$5,519	$658	$245	0.57	0.07	0.03
Panama*	$2,213	$145	Cost saving	0.14	0.01	Cost saving	$1,798	Cost saving	Cost saving	0.12	Cost saving	Cost saving
Paraguay*	$6,322	$2,129	$1,767	1.09	0.37	0.30	$6,175	$1,968	$1,606	1.06	0.34	0.28
Peru*	$7,636	$2,256	$1,819	1.10	0.32	0.26	$7,373	$1,973	$1,536	1.06	0.28	0.22
Saint Lucia	$23,916	$5,206	$3,595	2.26	0.49	0.34	$21,781	$3,017	$1,406	2.06	0.29	0.13
Saint Vincent and the Grenadines	$24,815	$6,452	$4,854	3.37	0.88	0.66	$23,236	$4,833	$3,235	3.16	0.66	0.44
Suriname	$8,199	$2,758	$2,222	1.32	0.44	0.36	$7,819	$2,366	$1,830	1.25	0.38	0.29
Venezuela*	$2,631	$1,245	$1,128	0.16	0.07	0.07	$2,498	$1,109	$992	0.15	0.07	0.06
Eastern Mediterranean Region												
Egypt	$2,843	$1,048	$871	1.12	0.41	0.34	$2,779	$982	$805	1.09	0.39	0.32
Iran	$6,500	$1,625	$1,198	1.17	0.29	0.22	$6,016	$1,128	$701	1.08	0.20	0.13
Iraq*	$3,809	$1,308	$1,096	0.65	0.22	0.19	$3,728	$1,220	$1,008	0.64	0.21	0.17
Jordan*	$9,297	$3,318	$2,701	2.19	0.78	0.64	$8,914	$2,927	$2,310	2.10	0.69	0.54
Lebanon	$6,328	Cost saving	Cost saving	0.77	Cost saving	Cost saving	$4,869	Cost saving	Cost saving	0.59	Cost saving	Cost saving
Libya*	$17,619	$4,497	$3,366	2.43	0.62	0.46	$16,603	$3,394	$2,263	2.29	0.47	0.31
Morocco*	$3,234	$1,140	$937	0.99	0.35	0.29	$3,149	$1,048	$845	0.96	0.32	0.26
State of Palestine*	$9,832	$3,604	$2,986	3.07	1.13	0.93	$9,564	$3,326	$2,708	2.99	1.04	0.85

	Cost per DALY averted Government perspective**			Cost per DALY averted as a share of GDP per capita Government perspective**			Cost per DALY averted Societal perspective**			Cost per DALY averted as a share of GDP per capita Societal perspective**		
	ROTARIX	ROTAVAC	ROTASIIL	ROTARIX	ROTAVAC	ROTASIIL	ROTARIX	ROTAVAC	ROTASIIL	ROTARIX	ROTAVAC	ROTASIIL
Syrian Arab Republic	$7,423	$2,605	$2,207	N/A	N/A	N/A	$7,345	$2,525	$2,127	N/A	N/A	N/A
Tunisia	$10,930	$3,324	$2,636	3.17	0.96	0.76	$10,455	$2,831	$2,143	3.03	0.82	0.62
Europe Region												
Albania*	$15,032	$4,520	$3,597	2.85	0.86	0.68	$14,351	$3,822	$2,898	2.72	0.73	0.55
Belarus	$19,082	$5,471	$4,063	3.03	0.87	0.65	$17,539	$3,919	$2,512	2.79	0.62	0.40
Bosnia and Herzegovina	$18,861	$5,025	$3,816	3.11	0.83	0.63	$17,575	$3,729	$2,520	2.90	0.61	0.42
Bulgaria*	$11,691	$2,355	$1,453	1.26	0.25	0.16	$10,238	$890	Cost saving	1.10	0.10	Cost saving
Croatia	$18,200	$1,901	$498	1.22	0.13	0.03	$15,330	Cost saving	Cost saving	1.03	Cost saving	Cost saving
Kazakhstan	$8,489	$2,297	$1,775	0.87	0.23	0.18	$7,942	$1,714	$1,192	0.81	0.17	0.12
Kosovo	$47,998	$17,319	$14,619	11.16	4.03	3.40	$47,448	$16,719	$14,018	11.03	3.89	3.26
Montenegro	$22,212	$6,276	$4,722	2.51	0.71	0.53	$20,524	$4,574	$3,020	2.32	0.52	0.34
Romania	$9,977	Cost saving	Cost saving	0.81	Cost saving	Cost saving	$8,147	Cost saving	Cost saving	0.66	Cost saving	Cost saving
Russian Federation	$10,335	Cost saving	Cost saving	0.90	Cost saving	Cost saving	$7,777	Cost saving	Cost saving	0.68	Cost saving	Cost saving
Serbia	$22,955	$7,316	$5,765	3.17	1.01	0.80	$21,637	$5,986	$4,435	2.99	0.83	0.61
North Macedonia*	$10,495	$2,998	$2,223	1.73	0.49	0.37	$9,647	$2,141	$1,367	1.59	0.35	0.22
Turkey	$15,987	$3,946	$2,895	1.71	0.42	0.31	$14,728	$2,655	$1,603	1.57	0.28	0.17
Turkmenistan*	$3,732	$1,297	$1,061	0.54	0.19	0.15	$3,620	$1,174	$938	0.52	0.17	0.13
South East Asia Region												
Maldives	$11,875	$2,185	$1,333	1.15	0.21	0.13	$10,340	$638	Cost saving	1.00	0.06	Cost saving
Thailand*	$6,684	$1,505	$1,052	0.92	0.21	0.14	$6,003	$818	$365	0.83	0.11	0.05
Western Pacific Region												
China	$9,762	$3,078	$2,389	1.00	0.32	0.24	$9,001	$2,302	$1,613	0.92	0.24	0.17
Fiji*	$2,998	$1,048	$848	0.48	0.17	0.14	$2,852	$898	$699	0.46	0.14	0.11
Malaysia	$11,097	$2,432	$1,561	0.98	0.21	0.14	$9,607	$928	$57	0.84	0.08	0.00
Micronesia*	$5,022	$1,562	$1,286	1.41	0.44	0.36	$4,910	$1,442	$1,166	1.38	0.40	0.33
Philippines	$2,408	$936	$787	0.78	0.30	0.25	$2,375	$900	$751	0.77	0.29	0.24
Samoa	$9,676	$2,934	$2,372	2.31	0.70	0.57	$9,320	$2,570	$2,008	2.23	0.61	0.48
Tonga	$9,754	$3,380	$2,748	2.24	0.77	0.63	$9,400	$3,017	$2,385	2.15	0.69	0.55
Tuvalu	$20,931	$6,820	$5,470	5.66	1.84	1.48	$20,241	$6,067	$4,717	5.47	1.64	1.27
Vanuatu	$3,179	$1,655	$1,487	1.02	0.53	0.48	$3,123	$1,594	$1,426	1.00	0.51	0.46

Results highlighted in light grey remain unchanged
*Countries using rotavirus vaccine as part of their national immunization program, as of July 2020. [7,10]
** discounted values

Table S13: Risk benefit of age-restricted and age-unrestricted schedules. 10 cohorts vaccinated, 2020-2029.

Results for 2-dose vaccines.

	Averted RVGE hospitalizations			Excess intussusception hospitalizations			RVGE hospitalizations averted per excess intussusception hospitalization		
	Age-restricted schedule	Age unrestricted schedule	Age unrestricted vs age-restricted	Age-restricted schedule	Age unrestricted schedule	Age unrestricted vs age-restricted	Age-restricted schedule	Age unrestricted schedule	Age unrestricted vs age-restricted
All 63 MICs	2,935,229.8	4,324,461.4	1,389,231.6	2,892.1	6,686.9	3,794.7	1,014.9	646.7	366.1
Africa Region	116,371.8	182,887.7	66,515.9	92.8	192.9	100.0	1,253.5	948.2	664.9
Algeria	31,215.5	89,788.2	58,572.7	24.6	100.1	75.6	1,271.4	896.9	775.2
Botswana*	5,348.0	6,112.6	764.6	2.8	3.6	0.9	1,928.0	1,683.7	892.6
Cabo Verde	1,486.0	1,690.4	204.4	0.6	0.7	0.2	2,699.9	2,384.5	1,289.6
Equatorial Guinea	1,239.1	1,359.8	120.7	0.8	1.0	0.3	1,618.5	1,313.4	447.5
Gabon	4,130.8	4,727.7	596.9	1.9	2.7	0.9	2,179.2	1,721.2	701.2
Mauritius*	1,688.3	1,932.6	244.3	0.5	0.7	0.2	3,495.1	2,834.4	1,229.1
Namibia*	4,919.0	4,950.6	31.6	2.0	2.0	0.1	2,507.4	2,443.1	489.2
South Africa*	63,197.2	69,177.8	5,980.5	59.2	81.2	22.1	1,068.4	851.6	270.8
Eswatini*	3,147.8	3,148.0	0.2	0.7	0.7	0.0	4,464.2	4,463.0	758.7
Americas Region	707,370.6	807,844.3	100,473.7	390.4	540.6	150.2	1,811.8	1,494.3	668.9
Argentina*	69,085.8	78,013.7	8,927.9	25.6	32.1	6.6	2,702.2	2,427.0	1,357.2
Belize	749.4	903.8	154.4	0.3	0.4	0.1	2,532.1	2,206.5	1,358.6
Brazil*	260,878.6	295,028.6	34,150.0	21.3	26.4	5.1	12,255.2	11,179.6	6,692.7
Colombia*	64,236.2	72,705.2	8,469.0	33.1	41.7	8.6	1,941.3	1,742.2	980.0
Costa Rica*	9,883.8	10,176.8	293.0	1.7	1.8	0.1	5,701.0	5,560.1	3,032.2
Dominican Republic*	18,944.2	21,408.1	2,463.9	10.2	12.8	2.6	1,858.3	1,668.4	934.3
Ecuador*	24,337.6	27,678.7	3,341.1	14.0	17.8	3.8	1,733.0	1,551.0	878.7
El Salvador*	11,997.9	12,081.4	83.6	4.9	5.0	0.1	2,428.1	2,412.3	1,246.6
Grenada	211.9	240.2	28.3	0.1	0.1	0.0	3,156.5	2,716.4	1,329.4
Guatemala*	20,356.1	24,834.1	4,478.0	19.2	27.0	7.8	1,061.6	920.2	573.0

Jamaica	4,396.9	4,960.1	563.2	1.7	2.3	0.6	2,569.6	2,176.8	992.5
Mexico*	131,171.0	159,933.4	28,762.3	202.9	303.4	100.5	646.4	527.2	286.3
Panama*	8,742.1	9,825.3	1,083.2	2.5	3.1	0.6	3,494.7	3,145.7	1,742.0
Paraguay*	6,924.8	7,893.2	968.4	6.3	8.0	1.7	1,097.6	989.7	581.2
Peru*	28,199.0	29,710.0	1,511.0	20.1	22.2	2.0	1,402.4	1,341.0	738.0
Saint Lucia	137.4	155.5	18.1	0.1	0.1	0.0	1,427.7	1,280.8	719.6
Saint Vincent and the Grenadines	101.1	114.8	13.7	0.1	0.1	0.0	1,405.9	1,259.1	711.7
Suriname	536.1	927.6	391.5	0.3	0.6	0.4	2,076.2	1,497.4	1,083.7
Venezuela**	46,480.6	51,253.7	4,773.2	26.1	35.6	9.5	1,783.5	1,439.6	500.3
Eastern Mediterranean Region	693,738.5	760,950.0	67,211.4	704.1	864.6	160.5	985.3	880.1	418.6
Egypt	272,832.9	277,024.6	4,191.7	195.8	201.9	6.1	1,393.4	1,371.9	683.3
Iran	191,020.2	217,747.1	26,727.0	170.9	218.2	47.3	1,118.0	997.9	564.5
Iraq*	74,172.4	82,479.9	8,307.5	138.3	173.2	34.9	536.3	476.2	238.1
Jordan*	13,762.8	27,629.1	13,866.3	17.4	48.5	31.0	789.1	569.8	446.6
Lebanon	14,414.5	16,114.7	1,700.1	12.5	15.5	3.0	1,154.8	1,041.5	568.8
Libya*	7,621.1	8,665.6	1,044.6	14.5	18.5	4.0	525.2	468.7	262.6
Morocco*	51,920.7	58,894.6	6,973.9	75.1	97.8	22.7	691.4	602.2	307.1
State of Palestine*	18,476.6	18,578.1	101.4	22.3	22.5	0.2	828.9	824.8	430.7
Syrian Arab Republic	28,108.8	31,074.0	2,965.2	37.1	46.1	8.9	756.9	674.5	332.0
Tunisia	21,408.5	22,742.3	1,333.8	20.2	22.5	2.3	1,061.4	1,012.7	583.5
Europe Region	293,792.4	453,649.0	159,856.6	193.2	327.4	134.2	1,520.5	1,385.5	1,191.1
Albania*	2,533.0	2,886.0	353.0	1.9	2.3	0.4	1,363.1	1,261.0	820.5
Belarus	6,032.5	12,788.3	6,755.8	2.2	5.6	3.4	2,716.4	2,276.2	1,988.5
Bosnia and Herzegovina	1,922.7	2,813.7	890.9	0.6	1.1	0.5	2,993.7	2,513.0	1,866.3
Bulgaria*	6,878.9	7,842.7	963.8	3.4	4.2	0.8	2,015.9	1,859.6	1,197.1
Croatia	2,489.0	2,823.1	334.1	2.1	2.6	0.5	1,188.0	1,105.3	727.8
Kazakhstan	22,322.1	30,209.7	7,887.6	17.0	27.6	10.5	1,311.2	1,095.9	748.1
Kosovo	571.4	655.2	83.8	1.0	1.2	0.2	576.2	536.5	365.1
Montenegro	468.1	575.1	106.9	0.4	0.6	0.1	1,108.5	1,012.6	734.5
Romania	22,702.1	25,599.7	2,897.5	10.6	12.9	2.2	2,132.2	1,985.7	1,290.7

Russian Federation	110,074.7	231,738.2	121,663.4	62.5	155.5	93.0	1,760.9	1,489.9	1,307.9
Serbia	5,454.7	6,540.4	1,085.7	4.9	6.3	1.5	1,117.9	1,032.9	747.5
North Macedonia*	1,167.1	2,647.4	1,480.3	0.7	2.0	1.3	1,633.7	1,342.1	1,176.5
Turkey	106,117.4	120,715.0	14,597.6	78.5	96.5	18.0	1,351.8	1,251.1	811.7
Turkmenistan*	5,058.6	5,814.6	755.9	7.3	9.1	1.8	693.6	642.2	429.3
South East Asia Region	111,752.7	127,422.1	15,669.4	45.6	56.6	11.0	2,450.5	2,252.6	1,429.4
Maldives	815.5	932.9	117.5	0.6	0.8	0.2	1,361.8	1,196.6	649.5
Thailand*	110,937.2	126,489.2	15,551.9	45.0	55.8	10.8	2,464.9	2,267.3	1,442.4
Western Pacific Region	1,012,203.8	1,991,708.3	979,504.5	1,466.0	4,704.7	3,238.8	690.5	423.3	302.4
China	857,135.9	1,814,703.0	957,567.1	1,336.7	4,530.5	3,193.8	641.2	400.6	299.8
Fiji*	1,828.8	2,068.7	240.0	1.4	1.9	0.5	1,336.5	1,114.8	492.4
Malaysia	66,383.3	75,948.8	9,565.5	11.2	14.9	3.7	5,934.0	5,085.3	2,552.3
Micronesia*	148.7	164.0	15.4	0.3	0.3	0.1	572.1	524.2	289.6
Philippines	85,880.0	97,900.6	12,020.6	115.4	155.7	40.3	744.4	628.7	298.0
Samoa	234.8	257.9	23.1	0.2	0.3	0.1	1,150.1	989.6	408.7
Tonga	184.4	206.6	22.2	0.2	0.2	0.1	1,072.9	903.2	390.5
Tuvalu	16.4	18.4	2.0	0.0	0.0	0.0	740.6	633.5	289.9
Vanuatu	391.6	440.2	48.7	0.7	0.9	0.2	571.6	487.4	223.1

*Countries using rotavirus vaccine as part of their national immunization program, as of July 2020. [7,10]

Cost-effectiveness acceptability curves, ROTARIX compared to no vaccination (age-restricted schedules).

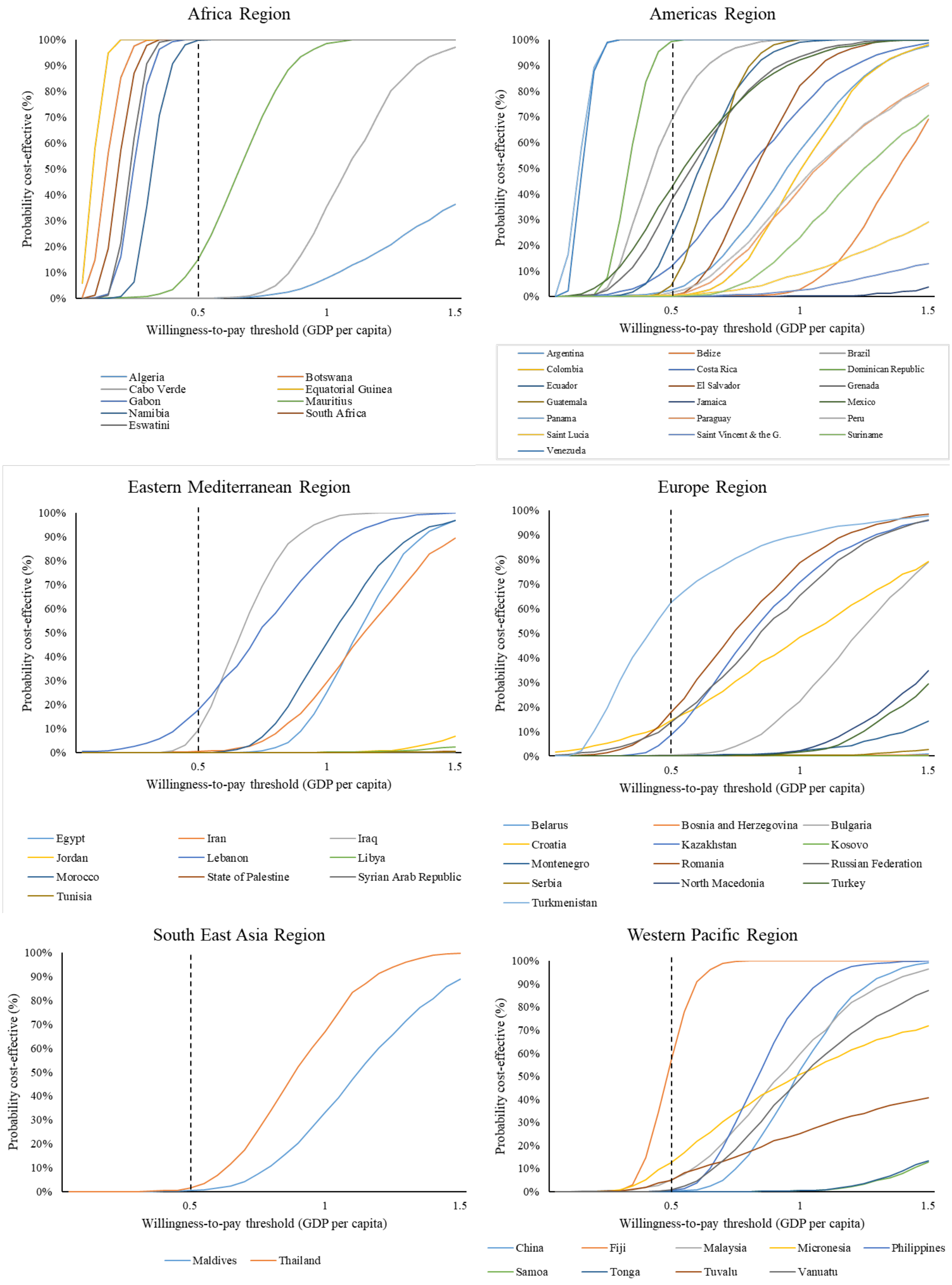

Cost-effectiveness acceptability curves, ROTAVAC compared to no vaccination (age-restricted schedules).

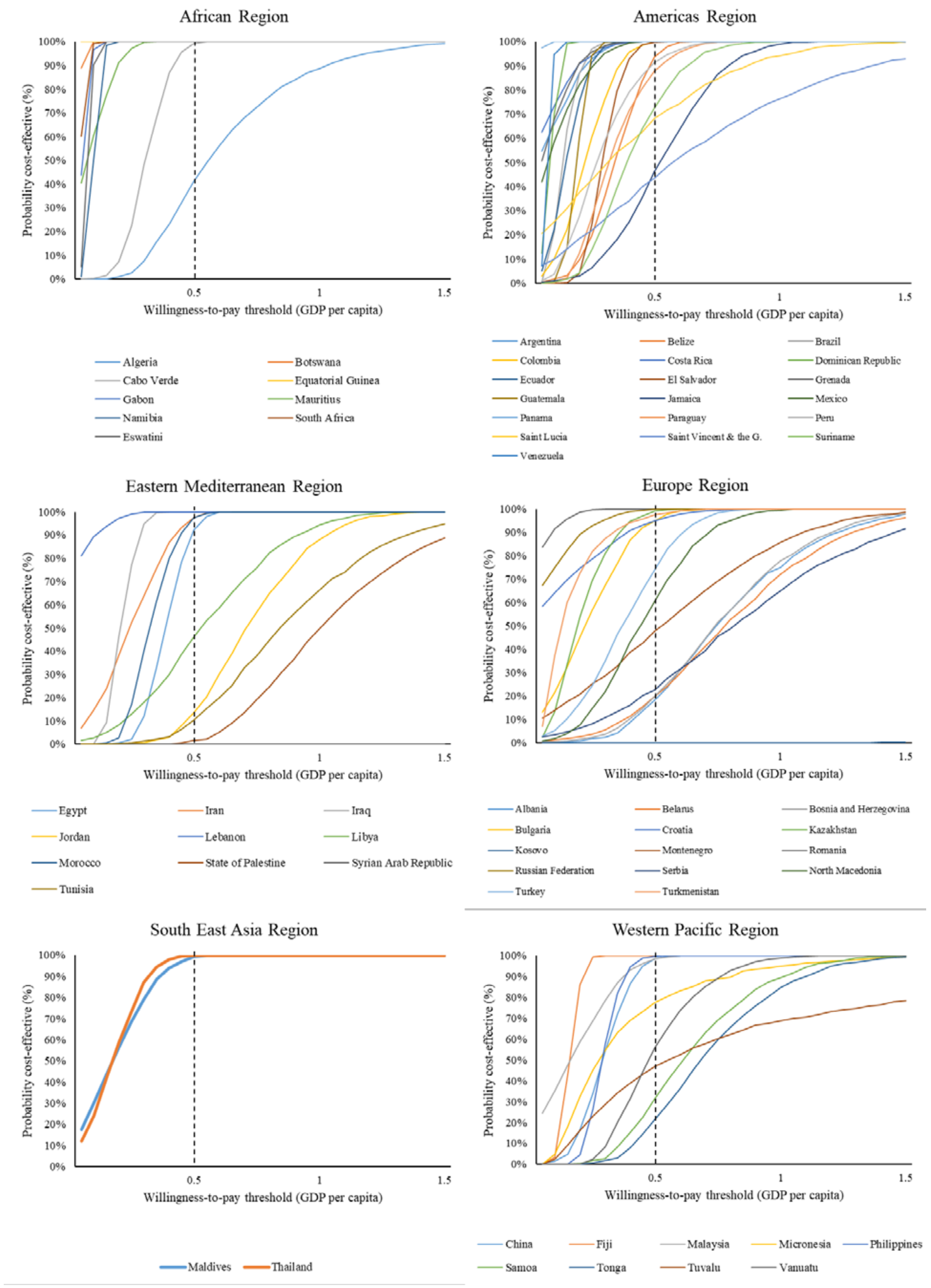

Table S14: 10-year undiscounted net cost. 10 cohorts vaccinated, 2020 – 2029.

Results for age-restricted schedules.

Country	Vaccine	Number of doses procured	Number of Fully Immunized Child	Vaccination costs Undiscounted	Total averted healthcare costs government perspective Undiscounted	Net cost Undiscounted	Net cost per Fully Immunized Child
Albania*	ROTARIX	527,581	250,601	$6,948,908	$730,157	$6,218,750	$25
Albania*	ROTAVAC	886,348	181,286	$2,612,290	$749,149	$1,863,141	$10
Albania*	ROTASIIL	758,320	181,286	$2,229,461	$749,149	$1,480,313	$8
Algeria	ROTARIX	6,008,635	2,854,101	$79,141,228	$5,763,712	$73,377,516	$26
Algeria	ROTAVAC	11,119,876	2,854,101	$32,773,053	$6,248,273	$26,524,781	$9
Algeria	ROTASIIL	9,513,671	2,854,101	$27,970,194	$6,248,273	$21,721,921	$8
Argentina*	ROTARIX	12,245,616	5,816,668	$161,290,068	$52,195,805	$109,094,263	$19
Argentina*	ROTAVAC	20,272,643	3,976,600	$59,748,547	$53,492,666	$6,255,881	$2
Argentina*	ROTASIIL	17,344,372	3,976,600	$50,992,454	$53,492,666	-$2,500,212	-$1
Belarus	ROTARIX	919,729	436,871	$12,113,976	$2,195,795	$9,918,181	$23
Belarus	ROTAVAC	1,702,095	436,871	$5,016,500	$2,207,962	$2,808,539	$6
Belarus	ROTASIIL	1,456,237	436,871	$4,281,337	$2,207,962	$2,073,375	$5
Belize	ROTARIX	134,376	63,829	$1,769,898	$249,825	$1,520,073	$24
Belize	ROTAVAC	225,602	46,057	$664,906	$256,211	$408,695	$9
Belize	ROTASIIL	193,015	46,057	$567,464	$256,211	$311,254	$7
Bosnia and Herzegovina	ROTARIX	294,812	140,036	$3,883,046	$577,763	$3,305,283	$24
Bosnia and Herzegovina	ROTAVAC	492,694	99,302	$1,452,091	$582,352	$869,739	$9
Bosnia and Herzegovina	ROTASIIL	421,527	99,302	$1,239,288	$582,352	$656,936	$7
Botswana*	ROTARIX	951,329	451,881	$12,530,187	$2,957,393	$9,572,793	$21
Botswana*	ROTAVAC	1,686,838	395,103	$4,971,533	$3,217,536	$1,753,997	$4
Botswana*	ROTASIIL	1,443,184	395,103	$4,242,960	$3,217,536	$1,025,423	$3
Brazil*	ROTARIX	42,321,855	20,102,881	$557,431,726	$31,408,891	$526,022,835	$26
Brazil*	ROTAVAC	70,233,293	13,873,874	$206,995,073	$32,195,465	$174,799,608	$13
Brazil*	ROTASIIL	60,088,484	13,873,874	$176,660,143	$32,195,465	$144,464,678	$10
Bulgaria*	ROTARIX	1,006,881	478,268	$13,261,878	$3,323,892	$9,937,986	$21
Bulgaria*	ROTAVAC	1,790,570	422,202	$5,277,257	$3,349,490	$1,927,767	$5
Bulgaria*	ROTASIIL	1,531,932	422,202	$4,503,880	$3,349,490	$1,154,390	$3
Cabo Verde	ROTARIX	175,376	83,304	$2,309,919	$228,053	$2,081,866	$25
Cabo Verde	ROTAVAC	293,845	59,653	$866,034	$233,940	$632,093	$11
Cabo Verde	ROTASIIL	251,400	59,653	$739,117	$233,940	$505,177	$8
China	ROTARIX	143,269,648	68,053,083	$1,887,040,357	$269,777,468	$1,617,262,889	$24
China	ROTAVAC	265,141,882	68,053,083	$781,439,411	$274,776,551	$506,662,860	$7
China	ROTASIIL	226,843,610	68,053,083	$666,920,213	$274,776,551	$392,143,662	$6
Colombia*	ROTARIX	11,512,474	5,468,425	$151,633,675	$25,235,096	$126,398,579	$23
Colombia*	ROTAVAC	19,218,393	3,861,312	$56,641,409	$25,861,894	$30,779,515	$8
Colombia*	ROTASIIL	16,442,403	3,861,312	$48,340,665	$25,861,894	$22,478,771	$6
Costa Rica*	ROTARIX	1,239,335	588,684	$16,323,587	$6,168,779	$10,154,809	$17
Costa Rica*	ROTAVAC	2,162,473	487,736	$6,373,349	$6,228,127	$145,222	$0
Costa Rica*	ROTASIIL	1,850,116	487,736	$5,439,340	$6,228,127	-$788,786	-$2
Croatia	ROTARIX	594,157	282,225	$7,825,796	$2,369,699	$5,456,097	$19
Croatia	ROTAVAC	984,669	193,746	$2,902,067	$2,388,360	$513,707	$3
Croatia	ROTASIIL	842,439	193,746	$2,476,772	$2,388,360	$88,412	$0

Country	Vaccine	Number of doses procured	Number of Fully Immunized Child	Vaccination costs Undiscounted	Total averted healthcare costs government perspective Undiscounted	Net cost Undiscounted	Net cost per Fully Immunized Child
Dominican Republic*	ROTARIX	3,451,630	1,639,524	$45,462,275	$6,114,011	$39,348,264	$24
Dominican Republic*	ROTAVAC	5,721,019	1,126,137	$16,861,273	$6,266,578	$10,594,695	$9
Dominican Republic*	ROTASIIL	4,894,650	1,126,137	$14,390,270	$6,266,578	$8,123,692	$7
Ecuador*	ROTARIX	5,159,473	2,450,749	$67,956,703	$9,444,150	$58,512,553	$24
Ecuador*	ROTAVAC	8,641,253	1,752,266	$25,467,933	$9,687,594	$15,780,339	$9
Ecuador*	ROTASIIL	7,393,072	1,752,266	$21,735,631	$9,687,594	$12,048,038	$7
Egypt	ROTARIX	48,540,400	23,056,690	$639,337,744	$33,203,970	$606,133,774	$26
Egypt	ROTAVAC	87,425,756	21,204,452	$257,665,560	$34,287,232	$223,378,328	$11
Egypt	ROTASIIL	74,797,592	21,204,452	$219,904,919	$34,287,232	$185,617,687	$9
El Salvador*	ROTARIX	1,864,419	885,599	$24,556,731	$680,777	$23,875,954	$27
El Salvador*	ROTAVAC	3,262,148	740,656	$9,614,365	$701,664	$8,912,701	$12
El Salvador*	ROTASIIL	2,790,949	740,656	$8,205,389	$701,664	$7,503,725	$10
Equatorial Guinea	ROTARIX	347,246	153,304	$4,573,662	$1,303,248	$3,270,414	$21
Equatorial Guinea	ROTAVAC	556,860	98,898	$1,641,205	$1,395,662	$245,543	$2
Equatorial Guinea	ROTASIIL	476,424	98,898	$1,400,688	$1,395,662	$5,026	$0
Fiji*	ROTARIX	314,496	149,385	$4,142,300	$389,209	$3,753,090	$25
Fiji*	ROTAVAC	579,254	147,255	$1,707,206	$399,414	$1,307,792	$9
Fiji*	ROTASIIL	495,584	147,255	$1,457,016	$399,414	$1,057,602	$7
Gabon	ROTARIX	894,884	425,070	$11,786,746	$2,068,419	$9,718,327	$23
Gabon	ROTAVAC	1,600,584	382,310	$4,717,321	$2,236,673	$2,480,648	$6
Gabon	ROTASIIL	1,369,389	382,310	$4,026,002	$2,236,673	$1,789,329	$5
Grenada	ROTARIX	29,222	13,881	$384,894	$120,222	$264,672	$19
Grenada	ROTAVAC	50,961	11,479	$150,196	$121,296	$28,900	$3
Grenada	ROTASIIL	43,600	11,479	$128,185	$121,296	$6,889	$1
Guatemala*	ROTARIX	6,659,331	3,163,182	$87,711,708	$4,240,629	$83,471,079	$26
Guatemala*	ROTAVAC	10,497,477	1,756,693	$30,938,689	$4,523,616	$26,415,073	$15
Guatemala*	ROTASIIL	8,981,175	1,756,693	$26,404,654	$4,523,616	$21,881,038	$12
Iran	ROTARIX	24,189,440	11,489,984	$318,605,157	$52,630,014	$265,975,143	$23
Iran	ROTAVAC	40,638,782	8,311,895	$119,772,651	$53,992,321	$65,780,330	$8
Iran	ROTASIIL	34,768,736	8,311,895	$102,220,083	$53,992,321	$48,227,762	$6
Iraq*	ROTARIX	19,564,112	9,292,953	$257,683,807	$8,484,531	$249,199,276	$27
Iraq*	ROTAVAC	32,108,949	6,137,985	$94,633,101	$9,100,908	$85,532,193	$14
Iraq*	ROTASIIL	27,470,990	6,137,985	$80,764,711	$9,100,908	$71,663,802	$12
Jamaica	ROTARIX	779,643	370,330	$10,268,872	$1,485,378	$8,783,495	$24
Jamaica	ROTAVAC	1,301,316	261,353	$3,835,304	$1,532,736	$2,302,569	$9
Jamaica	ROTASIIL	1,113,348	261,353	$3,273,244	$1,532,736	$1,740,508	$7
Jordan*	ROTARIX	1,978,216	939,652	$26,055,573	$2,257,392	$23,798,181	$25
Jordan*	ROTAVAC	3,660,984	939,652	$10,789,834	$2,305,712	$8,484,122	$9
Jordan*	ROTASIIL	3,132,175	939,652	$9,208,594	$2,305,712	$6,902,882	$7
Kazakhstan	ROTARIX	4,985,694	2,368,204	$65,667,816	$8,190,842	$57,476,974	$24
Kazakhstan	ROTAVAC	8,209,767	1,585,112	$24,196,235	$8,730,997	$15,465,238	$10
Kazakhstan	ROTASIIL	7,023,912	1,585,112	$20,650,300	$8,730,997	$11,919,303	$8
Kosovo	ROTARIX	281,541	133,732	$3,708,247	$77,352	$3,630,895	$27
Kosovo	ROTAVAC	472,995	96,742	$1,394,035	$84,445	$1,309,589	$14
Kosovo	ROTASIIL	404,674	96,742	$1,189,740	$84,445	$1,105,295	$11
Lebanon	ROTARIX	1,766,089	838,892	$23,261,597	$9,932,407	$13,329,190	$16

Country	Vaccine	Number of doses procured	Number of Fully Immunized Child	Vaccination costs Undiscounted	Total averted healthcare costs government perspective Undiscounted	Net cost Undiscounted	Net cost per Fully Immunized Child
Lebanon	ROTAVAC	2,860,342	524,679	$8,430,143	$10,005,113	-$1,574,971	-$3
Lebanon	ROTASIIL	2,447,181	524,679	$7,194,713	$10,005,113	-$2,810,400	-$5
Libya*	ROTARIX	2,054,231	975,760	$27,056,795	$3,915,103	$23,141,691	$24
Libya*	ROTAVAC	3,441,799	698,665	$10,143,842	$4,253,738	$5,890,104	$8
Libya*	ROTASIIL	2,944,650	698,665	$8,657,272	$4,253,738	$4,403,533	$6
Malaysia	ROTARIX	9,391,145	4,460,794	$123,693,122	$30,313,397	$93,379,725	$21
Malaysia	ROTAVAC	17,115,083	4,257,026	$50,442,428	$30,601,131	$19,841,296	$5
Malaysia	ROTASIIL	14,642,904	4,257,026	$43,050,138	$30,601,131	$12,449,007	$3
Maldives	ROTARIX	110,154	52,323	$1,450,860	$346,759	$1,104,101	$21
Maldives	ROTAVAC	185,060	37,851	$545,419	$349,602	$195,817	$5
Maldives	ROTASIIL	158,329	37,851	$465,489	$349,602	$115,887	$3
Mauritius*	ROTARIX	223,469	106,148	$2,943,361	$946,029	$1,997,332	$19
Mauritius*	ROTAVAC	410,209	103,566	$1,208,989	$965,853	$243,136	$2
Mauritius*	ROTASIIL	350,957	103,566	$1,031,813	$965,853	$65,960	$1
Mexico*	ROTARIX	31,662,977	15,039,914	$417,040,988	$86,297,574	$330,743,414	$22
Mexico*	ROTAVAC	52,292,669	10,185,526	$154,119,568	$88,189,821	$65,929,747	$6
Mexico*	ROTASIIL	44,739,283	10,185,526	$131,533,492	$88,189,821	$43,343,672	$4
Micronesia*	ROTARIX	45,211	21,290	$595,489	$33,776	$561,713	$26
Micronesia*	ROTAVAC	71,515	12,116	$210,772	$36,290	$174,483	$14
Micronesia*	ROTASIIL	61,185	12,116	$179,884	$36,290	$143,594	$12
Montenegro	ROTARIX	111,499	52,962	$1,468,587	$244,706	$1,223,881	$23
Montenegro	ROTAVAC	199,353	47,577	$587,542	$246,607	$340,935	$7
Montenegro	ROTASIIL	170,557	47,577	$501,438	$246,607	$254,831	$5
Morocco*	ROTARIX	11,428,154	5,428,373	$150,523,077	$9,947,068	$140,576,008	$26
Morocco*	ROTAVAC	20,458,361	4,896,192	$60,295,905	$10,771,409	$49,524,496	$10
Morocco*	ROTASIIL	17,503,265	4,896,192	$51,459,598	$10,771,409	$40,688,189	$8
Namibia*	ROTARIX	1,324,912	624,082	$17,450,751	$1,374,600	$16,076,152	$26
Namibia*	ROTAVAC	2,419,746	604,537	$7,131,595	$1,486,590	$5,645,006	$9
Namibia*	ROTASIIL	2,070,227	604,537	$6,086,467	$1,486,590	$4,599,877	$8
State of Palestine*	ROTARIX	2,974,180	1,411,744	$39,173,667	$2,257,620	$36,916,047	$26
State of Palestine*	ROTAVAC	5,376,191	1,314,196	$15,844,979	$2,334,574	$13,510,405	$10
State of Palestine*	ROTASIIL	4,599,630	1,314,196	$13,522,913	$2,334,574	$11,188,338	$9
Panama*	ROTARIX	1,388,971	659,761	$18,294,481	$5,845,929	$12,448,552	$19
Panama*	ROTAVAC	2,281,848	437,501	$6,725,176	$5,987,610	$737,566	$2
Panama*	ROTASIIL	1,952,248	437,501	$5,739,608	$5,987,610	-$248,002	-$1
Paraguay*	ROTARIX	2,315,971	1,100,086	$30,504,227	$1,465,538	$29,038,689	$26
Paraguay*	ROTAVAC	3,856,809	769,571	$11,366,980	$1,596,204	$9,770,775	$13
Paraguay*	ROTASIIL	3,299,714	769,571	$9,701,160	$1,596,204	$8,104,955	$11
Peru*	ROTARIX	9,917,609	4,710,864	$130,627,301	$10,720,156	$119,907,145	$25
Peru*	ROTAVAC	15,902,848	2,823,465	$46,869,669	$11,540,080	$35,329,590	$13
Peru*	ROTASIIL	13,605,770	2,823,465	$40,000,964	$11,540,080	$28,460,885	$10
Philippines	ROTARIX	26,461,724	12,569,319	$348,533,982	$10,521,430	$338,012,552	$27
Philippines	ROTAVAC	48,494,735	12,202,308	$142,926,107	$11,532,191	$131,393,916	$11
Philippines	ROTASIIL	41,489,940	12,202,308	$121,980,423	$11,532,191	$110,448,232	$9
Romania	ROTARIX	3,017,201	1,433,171	$39,740,311	$12,877,841	$26,862,470	$19
Romania	ROTAVAC	3,722,768	**	$10,971,929	$12,877,894	-$1,905,965	*

Country	Vaccine	Number of doses procured	Number of Fully Immunized Child	Vaccination costs Undiscounted	Total averted healthcare costs government perspective Undiscounted	Net cost Undiscounted	Net cost per Fully Immunized Child
Romania	ROTASIIL	3,185,035	**	$9,364,003	$12,877,894	-$3,513,891	*
Russian Federation	ROTARIX	14,802,437	7,031,157	$194,966,592	$83,113,711	$111,852,881	$16
Russian Federation	ROTAVAC	27,394,120	7,031,157	$80,737,319	$83,786,362	-$3,049,043	$0
Russian Federation	ROTASIIL	23,437,191	7,031,157	$68,905,342	$83,786,362	-$14,881,020	-$2
Saint Lucia	ROTARIX	35,317	16,776	$465,176	$90,128	$375,047	$22
Saint Lucia	ROTAVAC	58,697	11,645	$172,996	$92,397	$80,599	$7
Saint Lucia	ROTASIIL	50,219	11,645	$147,643	$92,397	$55,247	$5
Saint Vincent and the Grenadines	ROTARIX	26,418	12,549	$347,959	$52,463	$295,495	$24
Saint Vincent and the Grenadines	ROTAVAC	44,145	8,894	$130,106	$53,807	$76,299	$9
Saint Vincent and the Grenadines	ROTASIIL	37,768	8,894	$111,039	$53,807	$57,232	$6
Samoa	ROTARIX	44,863	20,067	$590,905	$50,508	$540,397	$27
Samoa	ROTAVAC	72,880	13,498	$214,796	$51,543	$163,253	$12
Samoa	ROTASIIL	62,353	13,498	$183,318	$51,543	$131,775	$10
Serbia	ROTARIX	1,302,859	618,858	$17,160,284	$2,182,250	$14,978,034	$24
Serbia	ROTAVAC	2,352,084	573,388	$6,932,179	$2,200,351	$4,731,828	$8
Serbia	ROTASIIL	2,012,338	573,388	$5,916,275	$2,200,351	$3,715,924	$6
South Africa*	ROTARIX	16,782,057	7,971,477	$221,040,671	$32,645,325	$188,395,346	$24
South Africa*	ROTAVAC	30,028,134	7,178,709	$88,500,417	$35,403,987	$53,096,430	$7
South Africa*	ROTASIIL	25,690,736	7,178,709	$75,530,765	$35,403,987	$40,126,778	$6
Suriname	ROTARIX	106,167	50,429	$1,398,351	$136,240	$1,262,111	$25
Suriname	ROTAVAC	191,256	46,408	$563,679	$140,498	$423,181	$9
Suriname	ROTASIIL	163,630	46,408	$481,072	$140,498	$340,574	$7
Eswatini*	ROTARIX	560,625	262,083	$7,384,134	$512,441	$6,871,693	$26
Eswatini*	ROTAVAC	1,020,728	253,367	$3,008,340	$554,249	$2,454,092	$10
Eswatini*	ROTASIIL	873,289	253,367	$2,567,471	$554,249	$2,013,222	$8
Syrian Arab Republic	ROTARIX	5,192,456	2,420,439	$68,391,132	$994,721	$67,396,411	$28
Syrian Arab Republic	ROTAVAC	8,369,744	1,511,870	$24,667,729	$1,018,105	$23,649,624	$16
Syrian Arab Republic	ROTASIIL	7,160,781	1,511,870	$21,052,697	$1,018,105	$20,034,591	$13
North Macedonia*	ROTARIX	172,951	82,152	$2,277,980	$415,093	$1,862,886	$23
North Macedonia*	ROTAVAC	320,072	82,152	$943,331	$418,840	$524,491	$6
North Macedonia*	ROTASIIL	273,839	82,152	$805,087	$418,840	$386,247	$5
Thailand	ROTARIX	11,762,988	5,587,419	$154,933,253	$30,241,218	$124,692,035	$22
Thailand	ROTAVAC	19,656,028	3,960,303	$57,931,228	$30,485,372	$27,445,855	$7
Thailand	ROTASIIL	16,816,824	3,960,303	$49,441,462	$30,485,372	$18,956,089	$5
Tonga	ROTARIX	39,167	18,605	$515,883	$44,458	$471,425	$25
Tonga	ROTAVAC	70,756	17,273	$208,535	$45,593	$162,941	$9
Tonga	ROTASIIL	60,535	17,273	$177,974	$45,593	$132,381	$8
Tunisia	ROTARIX	3,374,763	1,603,013	$44,449,851	$4,840,096	$39,609,755	$25
Tunisia	ROTAVAC	5,778,449	1,243,381	$17,030,535	$5,018,789	$12,011,745	$10
Tunisia	ROTASIIL	4,943,784	1,243,381	$14,534,726	$5,018,789	$9,515,937	$8
Turkey	ROTARIX	22,281,964	10,583,933	$293,481,315	$48,933,073	$244,548,242	$23
Turkey	ROTAVAC	37,333,742	7,579,116	$110,031,872	$50,195,818	$59,836,055	$8
Turkey	ROTASIIL	31,941,091	7,579,116	$93,906,807	$50,195,818	$43,710,989	$6
Turkmenistan*	ROTARIX	2,154,035	1,023,166	$28,371,329	$2,038,002	$26,333,327	$26
Turkmenistan*	ROTAVAC	3,856,092	922,858	$11,364,869	$2,221,016	$9,143,853	$10
Turkmenistan*	ROTASIIL	3,299,101	922,858	$9,699,358	$2,221,016	$7,478,342	$8

Country	Vaccine	Number of doses procured	Number of Fully Immunized Child	Vaccination costs Undiscounted	Total averted healthcare costs government perspective Undiscounted	Net cost Undiscounted	Net cost per Fully Immunized Child
Tuvalu	ROTARIX	5,025	2,387	$66,187	$6,334	$59,853	$25
Tuvalu	ROTAVAC	8,947	2,115	$26,369	$6,906	$19,463	$9
Tuvalu	ROTASIIL	7,655	2,115	$22,504	$6,906	$15,599	$7
Vanuatu	ROTARIX	155,458	73,843	$2,388,028	$123,984	$2,264,044	$31
Vanuatu	ROTAVAC	278,198	66,528	$1,313,737	$135,435	$1,178,302	$18
Vanuatu	ROTASIIL	238,014	66,528	$1,193,579	$135,435	$1,058,143	$16
Venezuela*	ROTARIX	7,616,897	3,525,625	$117,005,062	$1,447,763	$115,557,299	$33
Venezuela*	ROTAVAC	11,887,393	1,917,240	$56,135,867	$1,477,072	$54,658,795	$29
Venezuela*	ROTASIIL	10,170,325	1,917,240	$51,001,504	$1,477,072	$49,524,432	$26

*Countries using rotavirus vaccine as part of their national immunization program, as of July 2020. [7,10]

** Romania is using a particular three-dose standard schedule for DTP, giving doses at 2, 4 and 11 months. As a result, very few infants would receive the third dose before the upper age limit of 32 weeks implying a very low number of fully immunized child.

Table S15: 10-year undiscounted net cost. 10 cohorts vaccinated, 2020 – 2029.

Results for age-unrestricted schedules.

Country	Vaccine	Number of doses procured	Number of Fully Immunized Child	Vaccination costs Undiscounted	Total averted healthcare costs government perspective Undiscounted	Net cost Undiscounted	Net cost per Fully Immunized Child
Albania*	ROTARIX	608,027	288,502	$8,008,478	$831,910	$7,176,568	$25
Albania*	ROTAVAC	1,125,270	285,741	$3,316,451	$862,266	$2,454,184	$9
Albania*	ROTASIIL	962,731	285,741	$2,830,428	$862,266	$1,968,162	$7
Algeria	ROTARIX	17,501,850	8,178,477	$230,521,240	$16,570,775	$213,950,465	$26
Algeria	ROTAVAC	31,965,380	7,771,930	$94,209,966	$18,195,830	$76,014,136	$10
Algeria	ROTASIIL	27,348,158	7,771,930	$80,403,585	$18,195,830	$62,207,756	$8
Argentina*	ROTARIX	13,918,927	6,512,437	$183,329,673	$58,940,350	$124,389,323	$19
Argentina*	ROTAVAC	25,400,423	6,267,916	$74,861,396	$61,055,391	$13,806,005	$2
Argentina*	ROTASIIL	21,731,473	6,267,916	$63,890,530	$61,055,391	$2,835,139	$0
Belarus	ROTARIX	1,987,496	943,110	$26,177,812	$4,653,680	$21,524,132	$23
Belarus	ROTAVAC	3,678,201	938,939	$10,840,577	$4,685,816	$6,154,760	$7
Belarus	ROTASIIL	3,146,905	938,939	$9,251,900	$4,685,816	$4,566,084	$5
Belize	ROTARIX	164,414	77,776	$2,165,541	$301,268	$1,864,273	$24
Belize	ROTAVAC	303,489	76,546	$894,459	$312,050	$582,410	$8
Belize	ROTASIIL	259,652	76,546	$763,377	$312,050	$451,327	$6
Bosnia and Herzegovina	ROTARIX	434,332	195,162	$5,720,693	$845,447	$4,875,246	$25
Bosnia and Herzegovina	ROTAVAC	765,774	168,534	$2,256,926	$855,333	$1,401,594	$8
Bosnia and Herzegovina	ROTASIIL	655,162	168,534	$1,926,176	$855,333	$1,070,843	$6
Botswana*	ROTARIX	1,087,995	511,947	$14,330,254	$3,379,984	$10,950,270	$21
Botswana*	ROTAVAC	1,998,011	493,239	$5,888,638	$3,738,236	$2,150,402	$4
Botswana*	ROTASIIL	1,709,409	493,239	$5,025,663	$3,738,236	$1,287,428	$3
Brazil*	ROTARIX	48,214,423	22,611,289	$635,044,217	$35,519,541	$599,524,676	$27
Brazil*	ROTAVAC	88,191,970	21,867,982	$259,923,784	$36,799,151	$223,124,633	$10
Brazil*	ROTASIIL	75,453,130	21,867,982	$221,832,202	$36,799,151	$185,033,051	$8
Bulgaria*	ROTARIX	1,154,216	544,409	$15,202,467	$3,789,550	$11,412,917	$21
Bulgaria*	ROTAVAC	2,124,384	527,068	$6,261,090	$3,827,548	$2,433,542	$5
Bulgaria*	ROTASIIL	1,817,528	527,068	$5,343,534	$3,827,548	$1,515,986	$3
Cabo Verde	ROTARIX	201,605	95,418	$2,655,396	$259,403	$2,395,993	$25
Cabo Verde	ROTAVAC	372,152	94,025	$1,096,826	$268,863	$827,963	$9
Cabo Verde	ROTASIIL	318,397	94,025	$936,087	$268,863	$667,224	$7
China	ROTARIX	309,599,621	146,911,780	$4,077,814,005	$571,047,682	$3,506,766,322	$24
China	ROTAVAC	572,965,548	146,262,129	$1,688,672,713	$584,108,820	$1,104,563,893	$8
China	ROTASIIL	490,203,858	146,262,129	$1,441,199,343	$584,108,820	$857,090,523	$6
Colombia*	ROTARIX	13,175,331	6,251,175	$173,535,585	$28,560,910	$144,974,675	$23
Colombia*	ROTAVAC	24,383,407	6,159,809	$71,863,997	$29,602,757	$42,261,240	$7
Colombia*	ROTASIIL	20,861,360	6,159,809	$61,332,397	$29,602,757	$31,729,640	$5
Costa Rica*	ROTARIX	1,276,400	604,690	$16,811,786	$6,351,625	$10,460,161	$17
Costa Rica*	ROTAVAC	2,355,937	601,319	$6,943,536	$6,428,325	$515,211	$1
Costa Rica*	ROTASIIL	2,015,635	601,319	$5,925,968	$6,428,325	-$502,358	-$1
Croatia	ROTARIX	676,015	316,621	$8,903,959	$2,687,700	$6,216,258	$20
Croatia	ROTAVAC	1,234,874	305,382	$3,639,482	$2,719,628	$919,854	$3
Croatia	ROTASIIL	1,056,503	305,382	$3,106,120	$2,719,628	$386,491	$1

Country	Vaccine	Number of doses procured	Number of Fully Immunized Child	Vaccination costs Undiscounted	Total averted healthcare costs government perspective Undiscounted	Net cost Undiscounted	Net cost per Fully Immunized Child
Dominican Republic*	ROTARIX	3,927,691	1,839,827	$51,732,595	$6,908,734	$44,823,861	$24
Dominican Republic*	ROTAVAC	7,175,816	1,775,015	$21,148,925	$7,157,252	$13,991,673	$8
Dominican Republic*	ROTASIIL	6,139,310	1,775,015	$18,049,570	$7,157,252	$10,892,318	$6
Ecuador*	ROTARIX	5,928,888	2,805,001	$78,090,866	$10,740,115	$67,350,752	$24
Ecuador*	ROTAVAC	10,940,386	2,761,908	$32,244,052	$11,131,550	$21,112,502	$8
Ecuador*	ROTASIIL	9,360,108	2,761,908	$27,518,717	$11,131,550	$16,387,167	$6
Egypt	ROTARIX	49,335,214	23,373,036	$649,806,435	$33,713,337	$616,093,097	$26
Egypt	ROTAVAC	91,063,797	23,250,493	$268,387,776	$34,891,096	$233,496,680	$10
Egypt	ROTASIIL	77,910,138	23,250,493	$229,055,804	$34,891,096	$194,164,708	$8
El Salvador*	ROTARIX	1,876,517	888,620	$24,716,075	$685,510	$24,030,565	$27
El Salvador*	ROTAVAC	3,462,154	883,036	$10,203,834	$710,950	$9,492,885	$11
El Salvador*	ROTASIIL	2,962,065	883,036	$8,708,472	$710,950	$7,997,523	$9
Equatorial Guinea	ROTARIX	380,527	158,406	$5,012,017	$1,430,070	$3,581,947	$23
Equatorial Guinea	ROTAVAC	618,995	113,284	$1,824,333	$1,542,330	$282,003	$2
Equatorial Guinea	ROTASIIL	529,585	113,284	$1,556,978	$1,542,330	$14,649	$0
Fiji*	ROTARIX	360,881	170,864	$4,753,259	$440,222	$4,313,036	$25
Fiji*	ROTAVAC	667,859	168,542	$1,968,346	$452,550	$1,515,796	$9
Fiji*	ROTASIIL	571,390	168,542	$1,679,887	$452,550	$1,227,337	$7
Gabon	ROTARIX	1,023,603	471,313	$13,482,126	$2,367,091	$11,115,035	$24
Gabon	ROTAVAC	1,844,557	437,447	$5,436,371	$2,581,573	$2,854,798	$7
Gabon	ROTASIIL	1,578,121	437,447	$4,639,676	$2,581,573	$2,058,103	$5
Grenada	ROTARIX	33,361	15,765	$439,411	$136,278	$303,132	$19
Grenada	ROTAVAC	61,423	15,495	$181,030	$137,817	$43,213	$3
Grenada	ROTASIIL	52,551	15,495	$154,500	$137,817	$16,683	$1
Guatemala*	ROTARIX	8,174,644	3,788,872	$107,670,285	$5,172,473	$102,497,812	$27
Guatemala*	ROTAVAC	14,799,602	3,513,625	$43,618,128	$5,787,179	$37,830,949	$11
Guatemala*	ROTASIIL	12,661,882	3,513,625	$37,225,933	$5,787,179	$31,438,754	$9
Iran	ROTARIX	27,877,936	13,227,750	$367,187,266	$59,980,724	$307,206,543	$23
Iran	ROTAVAC	51,594,261	13,101,139	$152,061,185	$62,167,287	$89,893,898	$7
Iran	ROTASIIL	44,141,757	13,101,139	$129,776,764	$62,167,287	$67,609,477	$5
Iraq*	ROTARIX	22,057,003	10,233,323	$290,518,297	$9,429,537	$281,088,760	$27
Iraq*	ROTAVAC	39,912,686	9,674,726	$117,632,664	$10,364,960	$107,267,704	$11
Iraq*	ROTASIIL	34,147,520	9,674,726	$100,393,710	$10,364,960	$90,028,750	$9
Jamaica	ROTARIX	890,122	420,325	$11,724,023	$1,675,453	$10,048,570	$24
Jamaica	ROTAVAC	1,638,949	412,315	$4,830,393	$1,749,464	$3,080,929	$7
Jamaica	ROTASIIL	1,402,212	412,315	$4,122,504	$1,749,464	$2,373,039	$6
Jordan*	ROTARIX	4,252,951	2,007,800	$56,016,678	$4,529,294	$51,487,384	$26
Jordan*	ROTAVAC	7,830,079	1,978,317	$23,077,199	$4,637,188	$18,440,011	$9
Jordan*	ROTASIIL	6,699,067	1,978,317	$19,695,257	$4,637,188	$15,058,070	$8
Kazakhstan	ROTARIX	6,899,226	3,268,399	$90,871,430	$11,084,051	$79,787,379	$24
Kazakhstan	ROTAVAC	12,745,286	2,573,438	$37,563,545	$11,917,431	$25,646,114	$10
Kazakhstan	ROTASIIL	10,904,301	2,573,438	$32,058,643	$11,917,431	$20,141,212	$8
Kosovo	ROTARIX	324,468	153,958	$4,273,653	$88,691	$4,184,962	$27
Kosovo	ROTAVAC	600,469	152,484	$1,769,732	$99,896	$1,669,836	$11
Kosovo	ROTASIIL	513,734	152,484	$1,510,379	$99,896	$1,410,484	$9
Lebanon	ROTARIX	1,966,433	900,380	$25,900,383	$11,103,512	$14,796,871	$16

Country	Vaccine	Number of doses procured	Number of Fully Immunized Child	Vaccination costs Undiscounted	Total averted healthcare costs government perspective Undiscounted	Net cost Undiscounted	Net cost per Fully Immunized Child
Lebanon	ROTAVAC	3,511,349	827,004	$10,348,823	$11,231,123	-$882,301	-$1
Lebanon	ROTASIIL	3,004,154	827,004	$8,832,213	$11,231,123	-$2,398,911	-$3
Libya*	ROTARIX	2,361,412	1,117,604	$31,102,745	$4,451,121	$26,651,624	$24
Libya*	ROTAVAC	4,358,970	1,101,230	$12,846,975	$4,972,332	$7,874,643	$7
Libya*	ROTASIIL	3,729,341	1,101,230	$10,964,263	$4,972,332	$5,991,931	$5
Malaysia	ROTARIX	10,823,130	5,132,287	$142,554,147	$34,681,001	$107,873,146	$21
Malaysia	ROTAVAC	20,030,019	5,112,739	$59,033,474	$35,032,157	$24,001,317	$5
Malaysia	ROTASIIL	17,136,794	5,112,739	$50,382,175	$35,032,157	$15,350,018	$3
Maldives	ROTARIX	126,950	60,236	$1,672,084	$396,694	$1,275,390	$21
Maldives	ROTAVAC	234,941	59,660	$692,431	$401,420	$291,011	$5
Maldives	ROTASIIL	201,005	59,660	$590,956	$401,420	$189,536	$3
Mauritius*	ROTARIX	255,774	120,791	$3,368,861	$1,082,884	$2,285,976	$19
Mauritius*	ROTAVAC	472,130	118,538	$1,391,486	$1,109,078	$282,408	$2
Mauritius*	ROTASIIL	403,934	118,538	$1,187,565	$1,109,078	$78,487	$1
Mexico*	ROTARIX	39,586,395	18,429,793	$521,402,311	$105,200,161	$416,202,150	$23
Mexico*	ROTAVAC	72,852,475	16,757,275	$214,714,457	$108,940,666	$105,773,790	$6
Mexico*	ROTASIIL	62,329,340	16,757,275	$183,248,258	$108,940,666	$74,307,592	$4
Micronesia*	ROTARIX	49,575	22,110	$652,970	$37,263	$615,707	$28
Micronesia*	ROTAVAC	86,230	19,097	$254,140	$41,404	$212,736	$11
Micronesia*	ROTASIIL	73,774	19,097	$216,896	$41,404	$175,492	$9
Montenegro	ROTARIX	137,342	63,768	$1,808,967	$300,601	$1,508,366	$24
Montenegro	ROTAVAC	248,704	60,619	$732,994	$303,292	$429,702	$7
Montenegro	ROTASIIL	212,780	60,619	$625,574	$303,292	$322,282	$5
Morocco*	ROTARIX	13,170,697	6,245,515	$173,474,541	$11,282,043	$162,192,498	$26
Morocco*	ROTAVAC	24,374,790	6,112,295	$71,838,600	$12,363,606	$59,474,994	$10
Morocco*	ROTASIIL	20,853,987	6,112,295	$61,310,722	$12,363,606	$48,947,116	$8
Namibia*	ROTARIX	1,331,896	624,124	$17,542,733	$1,383,403	$16,159,330	$26
Namibia*	ROTAVAC	2,431,653	607,031	$7,166,689	$1,496,464	$5,670,225	$9
Namibia*	ROTASIIL	2,080,414	607,031	$6,116,418	$1,496,464	$4,619,954	$8
State of Palestine*	ROTARIX	2,987,501	1,411,786	$39,349,117	$2,270,000	$37,079,117	$26
State of Palestine*	ROTAVAC	5,500,463	1,397,231	$16,211,240	$2,352,002	$13,859,238	$10
State of Palestine*	ROTASIIL	4,705,952	1,397,231	$13,835,498	$2,352,002	$11,483,497	$8
Panama*	ROTARIX	1,567,404	727,899	$20,644,671	$6,570,128	$14,074,543	$19
Panama*	ROTAVAC	2,838,985	689,591	$8,367,200	$6,802,789	$1,564,410	$2
Panama*	ROTASIIL	2,428,910	689,591	$7,140,994	$6,802,789	$338,205	$0
Paraguay*	ROTARIX	2,647,098	1,245,590	$34,865,595	$1,670,246	$33,195,349	$27
Paraguay*	ROTAVAC	4,858,117	1,212,994	$14,318,084	$1,879,104	$12,438,980	$10
Paraguay*	ROTASIIL	4,156,389	1,212,994	$12,219,783	$1,879,104	$10,340,679	$9
Peru*	ROTARIX	10,433,047	4,869,403	$137,416,266	$11,294,339	$126,121,926	$26
Peru*	ROTAVAC	18,980,317	4,646,024	$55,939,738	$12,708,196	$43,231,542	$9
Peru*	ROTASIIL	16,238,715	4,646,024	$47,741,823	$12,708,196	$35,033,627	$8
Philippines	ROTARIX	30,249,283	14,267,677	$398,420,864	$11,992,426	$386,428,439	$27
Philippines	ROTAVAC	55,766,257	13,966,419	$164,357,101	$13,214,306	$151,142,795	$11
Philippines	ROTASIIL	47,711,131	13,966,419	$140,270,725	$13,214,306	$127,056,419	$9
Romania	ROTARIX	3,403,245	1,579,720	$44,824,987	$14,521,369	$30,303,618	$19
Romania	ROTAVAC	6,151,990	785,702	$18,131,454	$14,903,409	$3,228,045	$4

Country	Vaccine	Number of doses procured	Number of Fully Immunized Child	Vaccination costs Undiscounted	Total averted healthcare costs government perspective Undiscounted	Net cost Undiscounted	Net cost per Fully Immunized Child
Romania	ROTASIIL	5,263,370	785,702	$15,474,307	$14,903,409	$570,897	$1
Russian Federation	ROTARIX	31,987,675	15,172,219	$421,317,667	$174,964,319	$246,353,348	$16
Russian Federation	ROTAVAC	59,200,418	15,033,481	$174,478,433	$176,562,802	-$2,084,369	$0
Russian Federation	ROTASIIL	50,649,247	15,033,481	$148,908,786	$176,562,802	-$27,654,016	-$2
Saint Lucia	ROTARIX	40,292	18,923	$530,691	$102,013	$428,678	$23
Saint Lucia	ROTAVAC	73,807	18,355	$217,528	$105,698	$111,830	$6
Saint Lucia	ROTASIIL	63,146	18,355	$185,649	$105,698	$79,951	$4
Saint Vincent and the Grenadines	ROTARIX	30,292	14,301	$398,988	$59,577	$339,411	$24
Saint Vincent and the Grenadines	ROTAVAC	55,776	14,019	$164,386	$61,744	$102,641	$7
Saint Vincent and the Grenadines	ROTASIIL	47,719	14,019	$140,295	$61,744	$78,551	$6
Samoa	ROTARIX	49,134	20,734	$647,163	$55,461	$591,702	$29
Samoa	ROTAVAC	81,032	15,460	$238,821	$56,778	$182,043	$12
Samoa	ROTASIIL	69,327	15,460	$203,822	$56,778	$147,044	$10
Serbia	ROTARIX	1,571,300	742,088	$20,695,981	$2,616,439	$18,079,543	$24
Serbia	ROTAVAC	2,892,977	727,929	$8,526,327	$2,642,103	$5,884,223	$8
Serbia	ROTASIIL	2,475,103	727,929	$7,276,802	$2,642,103	$4,634,698	$6
South Africa*	ROTARIX	18,841,291	8,724,059	$248,163,358	$35,730,011	$212,433,348	$24
South Africa*	ROTAVAC	34,098,527	8,217,265	$100,496,883	$38,841,738	$61,655,145	$8
South Africa*	ROTASIIL	29,173,184	8,217,265	$85,769,161	$38,841,738	$46,927,423	$6
Suriname	ROTARIX	203,560	96,691	$2,681,146	$235,645	$2,445,501	$25
Suriname	ROTAVAC	376,719	96,241	$1,110,284	$244,402	$865,881	$9
Suriname	ROTASIIL	322,304	96,241	$947,573	$244,402	$703,170	$7
Eswatini*	ROTARIX	560,654	262,083	$7,384,508	$512,471	$6,872,038	$26
Eswatini*	ROTAVAC	1,021,104	253,627	$3,009,448	$554,360	$2,455,088	$10
Eswatini*	ROTASIIL	873,611	253,627	$2,568,416	$554,360	$2,014,056	$8
Syrian Arab Republic	ROTARIX	5,707,206	2,489,993	$75,171,043	$1,098,944	$74,072,098	$30
Syrian Arab Republic	ROTAVAC	9,711,219	2,038,979	$28,621,390	$1,130,819	$27,490,570	$13
Syrian Arab Republic	ROTASIIL	8,308,487	2,038,979	$24,426,952	$1,130,819	$23,296,133	$11
North Macedonia*	ROTARIX	400,446	186,689	$5,274,373	$941,532	$4,332,842	$23
North Macedonia*	ROTAVAC	729,484	177,487	$2,149,971	$951,096	$1,198,875	$7
North Macedonia*	ROTASIIL	624,114	177,487	$1,834,895	$951,096	$883,799	$5
Thailand	ROTARIX	13,488,095	6,367,496	$177,655,068	$34,480,342	$143,174,726	$22
Thailand	ROTAVAC	24,835,107	6,242,203	$73,195,269	$34,892,062	$38,303,207	$6
Thailand	ROTASIIL	21,247,814	6,242,203	$62,468,573	$34,892,062	$27,576,510	$4
Tonga	ROTARIX	44,291	20,661	$583,362	$49,808	$533,554	$26
Tonga	ROTAVAC	80,755	19,771	$238,005	$51,216	$186,788	$9
Tonga	ROTASIIL	69,090	19,771	$203,125	$51,216	$151,909	$8
Tunisia	ROTARIX	3,599,470	1,705,363	$47,409,523	$5,141,384	$42,268,139	$25
Tunisia	ROTAVAC	6,644,393	1,692,266	$19,582,688	$5,384,680	$14,198,008	$8
Tunisia	ROTASIIL	5,684,648	1,692,266	$16,712,864	$5,384,680	$11,328,184	$7
Turkey	ROTARIX	25,614,665	12,123,127	$337,377,159	$55,662,961	$281,714,198	$23
Turkey	ROTAVAC	47,284,702	11,946,146	$139,359,838	$57,692,057	$81,667,781	$7
Turkey	ROTASIIL	40,454,690	11,946,146	$118,936,787	$57,692,057	$61,244,730	$5
Turkmenistan*	ROTARIX	2,482,504	1,177,187	$32,697,680	$2,342,507	$30,355,173	$26
Turkmenistan*	ROTAVAC	4,594,711	1,152,077	$13,541,763	$2,592,171	$10,949,591	$10
Turkmenistan*	ROTASIIL	3,931,031	1,152,077	$11,557,230	$2,592,171	$8,965,059	$8

Country	Vaccine	Number of doses procured	Number of Fully Immunized Child	Vaccination costs Undiscounted	Total averted healthcare costs government perspective Undiscounted	Net cost Undiscounted	Net cost per Fully Immunized Child
Tuvalu	ROTARIX	5,621	2,592	$74,031	$7,106	$66,924	$26
Tuvalu	ROTAVAC	10,132	2,421	$29,862	$7,820	$22,041	$9
Tuvalu	ROTASIIL	8,668	2,421	$25,485	$7,820	$17,665	$7
Vanuatu	ROTARIX	174,549	80,830	$2,681,289	$139,385	$2,541,905	$31
Vanuatu	ROTAVAC	315,921	76,152	$1,492,844	$153,493	$1,339,350	$18
Vanuatu	ROTASIIL	270,288	76,152	$1,355,653	$153,493	$1,202,160	$16
Venezuela*	ROTARIX	8,359,408	3,661,427	$128,410,950	$1,592,695	$126,818,255	$35
Venezuela*	ROTAVAC	14,280,027	3,022,058	$67,454,703	$1,641,133	$65,813,570	$22
Venezuela*	ROTASIIL	12,217,357	3,022,058	$61,271,604	$1,641,133	$59,630,470	$20

*Countries in bold are using rotavirus vaccine as part of their national immunization program, as of July 2020. [7,10]

Table S16: Estimated rotavirus deaths averted by vaccination in 17 countries not using rotavirus vaccination with a 90% probability to have at least one of the rotavirus vaccine be cost-effective at 0.5 GDP p.c. threshold.

Results assuming a 3-dose age restricted schedule for 10 cohorts vaccinated over the period 2020-2029

	Estimated rotavirus deaths averted by vaccination
Belize	6
Cabo Verde	16
China	3,647
Croatia	1
Egypt	6,509
Equatorial Guinea	120
Gabon	174
Grenada	1
Iran	1,035
Kazakhstan	159
Lebanon	44
Malaysia	130
Maldives	1
Philippines	4,539
Romania	43
Russian Federation	123
Turkey	211
	**16,759**
